# Mechanistic Insights into the *In Situ* Restructuring of Coordinated Copper in Postmetalated MOFs for Photocatalysis

**DOI:** 10.1021/jacs.5c18407

**Published:** 2025-12-15

**Authors:** Zahraa Abou Khalil, Karen Hannouche, Akashdeep Nath, Nisrine Assaad, Leen Farhat, Georges Mouchaham, Dong Fan, Oleg Lebedev, Anthony Beauvois, Ali Youssef, Valerie Briois, Guillaume Clet, Guillaume Maurin, Christian Serre, Marco Daturi, Mohamad Hmadeh, Mohamad El-Roz

**Affiliations:** † 69240 Université de Caen Normandie, ENSICAEN, UNICAEN, CNRS, Laboratoire Catalyse et Spectrochimie, Caen 14000, France; ‡ Department of Chemistry, 11238 American University of Beirut, Beirut 1107 2020, Lebanon; § Institut des Matériaux Poreux de Paris, Ecole Normale Supérieure, ESPCI Paris, CNRS, 26909 PSL University, Paris 75005, France; ∥ ICGM, CNRS, ENSCM, 47912 Univ. Montpellier, Montpellier 34090, France; ⊥ Université de Caen Normandie, ENSICAEN, UNICAEN, CNRS, Laboratoire CRISMAT, Caen 14050, France; # 55536 Synchrotron SOLEIL, L’Orme des Merisiers, Départementale 128, Saint-Aubin 91190, France

## Abstract

Metal–organic frameworks (MOFs) offer a powerful platform for the rational design of photocatalysts, where systematic structural tuning can be employed for efficient solar-to-chemical energy conversion. Here, we establish a direct structure–activity relationship in a series of copper–metalated mixed-linker UiO-66 derivatives, UiO-66­(COOH)*_x_*-Cu (0 ≤ *x* ≤ 2), incorporating increasing densities of free carboxylate groups in their MOF backbone. These functionalities determine both Cu coordination and *in situ* restructuring under the photocatalytic dehydrogenation of formic acid (FAc). *Operando* Fourier-transform infrared (FTIR) and X-ray absorption spectroscopy (XAS) reveal that the −COOH content dictates the evolution of the surface of the framework and Cu speciation during photocatalysis. FTIR demonstrates that intraframework anhydride formation correlates linearly with photocatalytic efficiency, while XAS evidences a light-induced restructuring of coordinated Cu­(II)/Cu­(I) species into a catalytically active Cu­(I)/Cu(0) binary system in the presence of FAc. Time-resolved spectroscopy further indicates a photoinduced charge transfer from the Zr–oxo clusters to the Cu centers, enhancing the overall reactivity. Density functional theory (DFT) calculations corroborate these findings, showing that the number and spatial arrangement of carboxylates influence the Cu coordination stability and its restructuring dynamics. These insights reveal how linker functionalization governs metal speciation and dynamics in MOFs, offering design principles for next-generation light-driven catalytic systems.

## Graphical abstract

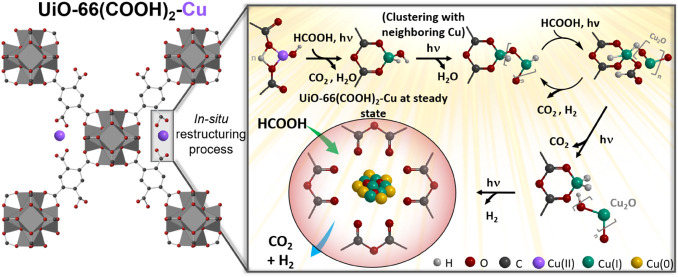

## Introduction

Photocatalysis offers a promising alternative to fossil-fuel-based processes by directly converting solar energy into chemical energy using a wide range of efficient catalysts. This sustainable approach gained immense interest as it addresses global energy and environmental challenges, particularly in applications such as solar-driven water splitting for hydrogen production, CO_2_ reduction, and environmental remediation.^1^ Benchmark inorganic photocatalysts, such as titanium dioxide (TiO_2_), zinc oxide (ZnO), and other semiconducting metal oxides, have been extensively studied due to their chemical stability, abundance, and strong oxidative power. However, their practical applications are often hindered by their limited structural tunability and wide bandgaps, which restricts their activity to the ultraviolet (UV) region.^2^ In contrast, organometallic complexes present an attractive alternative due to their well-defined molecular structures, tunable electronic properties, and ability to absorb light across the visible spectrum. Moreover, the coordination environment in metal complexes significantly impacts their photocatalytic activity and stability, with factors like electron-donating ligands influencing their optical and electrochemical behaviors.^3,4^ The structural flexibility of these complexes makes them promising catalytic candidates; however, they still encounter challenges such as stability, toxicity, and recyclability, limiting their practical use. Thus, various approaches have been employed to optimize the performance of both types of photocatalysts including support immobilization,^5^ encapsulation,^5^ doping,^6^ and heterojunction formation.^7^

Metal–organic frameworks (MOFs), a highly versatile class of hybrid crystalline porous solids,^8^ have already been considered for a large array of applications,^9^ while receiving significant interest as photocatalysts due to their ability to bridge the characteristics of solid semiconductors and molecular catalysts.^10^ However, semiconductor-like behavior is not inherent to all MOFs; rather, only specific subclasses, particularly those incorporating Ti, Zr, Fe, and Ce, exhibit the requisite electronic structures for efficient photocatalysis.^11^ In some cases, it is possible to synthesize them under green ambient conditions.^12,13^ These MOFs demonstrate key advantages, including high surface area, tunable bandgaps, redox versatility, and exceptional chemical stability, positioning them as promising candidates for different photocatalytic applications.^14–16^ A critical aspect of their photocatalytic potential lies in the flexibility and tunable nature of their frameworks, which can be precisely engineered to optimize their properties. To enhance their performance, various structural modification strategies have been employed to tune their electronic and optical properties.^17^ Through the rational selection of metal nodes and organic linkers, functional MOFs with photoactive sites can be designed, further expanding their applicability to diverse photocatalytic processes. One appealing approach to tune the MOFs’ properties is to mix different types and ratios of inorganic and/or organic units, yielding multivariate frameworks denoted as MTV-MOFs.^18,19^ Particularly, mixed-linker MTV-MOFs can outperform their monolinker counterparts in terms of stability, accessibility, and/or light harvesting ability, with the possibility of introducing free functional groups.^20,21^ However, only a handful of studies have closely examined the effect of organic composition in mixed-linker MOFs on their photocatalytic efficiency, highlighting the need for structural optimization and mechanistic elucidation. Within the same scope, other useful strategies include defect engineering (through which structural vacancies are introduced as potential redox and/or anchoring sites),^17^ crystal engineering (assessing the effect of MOFs size and morphology on their performance), and post-synthetic modification (introducing additional active sites to the MOF’s backbone).^22^ More specifically, introducing copper (Cu) as a photoactive center is one of the most promising approaches that could promote charge separation, extend visible-light absorption, and facilitate redox transformations. However, achieving precise control over the size, shape, and surface chemistry of Cu species remains a significant challenge, as these factors are crucial for optimizing catalytic efficiency, stability, and selectivity.^23,24^ Building on the potential of Cu in photocatalysis, several studies have explored Cu-based catalysts for dehydrogenation reactions. For instance, Abdelli et al.^25^ investigated a copper iron spinel (CuFe_2_O_4_) photocatalyst for FAc dehydrogenation under visible light, where the catalyst exhibited high activity due to the synergistic effect between copper and the spinel support, although the selectivity was limited to ∼70%. In contrast, the Cu_2_O/Fe_3_O_4_ composite catalyst showed significantly improved performance, achieving a high FAc conversion rate (9.89 mmol·g^–1^·h^–1^) and over 99% selectivity to H_2_.^26^ This enhancement was attributed to the efficient separation of the photogenerated charge carriers via a Z-scheme mechanism and the *in situ* restructuring of Cu_2_O into Cu^(0)^/Cu_2_O, highlighting the importance of copper in optimizing photocatalytic reactions. However, one of the main drawbacks of these materials is their rapid aging, as freshly synthesized Cu_2_O is deactivated under ambient conditions in about 2 weeks, requiring the preparation of fresh catalyst. Copper can also be introduced into MOFs via different techniques; in fact, some of us have previously prepared a Cu^(0)^@Zr-MOF material for the efficient photoreduction of CO_2_ through a ship-in-a-bottle ambient temperature green strategy.^27^ Another successful example was also recently reported by some of us, in which additional cationic copper sites were post-synthetically anchored on the free −COOH functionalities of a robust Zr-MOF.^28^ The resulting structure, termed UiO-66­(COOH)_2_-Cu, was evaluated for the photocatalytic FAc dehydrogenation under continuous vapor flow and visible light. Interestingly, high performance (5 mmol·g_cat_^–1^·h^–1^), selectivity (100% dehydrogenation), and stability were only attained with UiO-66­(COOH)_2_-Cu, demonstrating a comparable or even better activity than the heterojunction counterparts. The use of *operando* FTIR spectroscopy then showed that this enhanced performance is due to the progressive formation of an anhydride bridge involving the condensation of two free carboxylate groups under solar-simulated irradiation and in the presence of FAc. This phenomenon was only present in the fully functionalized MOF having free −COOH groups, unlike typical UiO-66-Cu, where no free carboxylate groups are present. While *ex situ* techniques, such as high-resolution transmission electron microscopy (HRTEM) and X-ray photoelectron spectroscopy (XPS), inferred that the nanoclustering of Cu^(I)^/Cu^(0)^ active sites within the framework occurred during the first cycle, there was no direct information on the evolution of the Cu oxidation state and its coordination environment. A key role of the free functionalities in Cu coordination and its *in situ* restructuring during the reaction can therefore be proposed. However, this phenomenon was not fully revealed, and the importance of copper-MOF synergy was not addressed. Furthermore, the contribution of the UiO-66 framework to the photocatalytic process was not evaluated.

To advance our understanding of how free carboxylate groups can promote the dynamic restructuring of Cu species within the UiO-66­(COOH)_2_ framework and subsequently enhance its photodehydrogenation efficiency, a MTV approach was used to design a series of materials with systematically varied ratios of 1,4-benzenedicarboxylic acid (BDC) and 1,2,4,5-benzenetetracarboxylic acid (BTCA), followed by Cu^(II)^ incorporation through post-synthetic modification. Different *operando* techniques were used to investigate the real-time performance of these samples and their mechanism of action. This in-depth investigation demonstrated a linear correlation between MOFs' photocatalytic activity and the concentration of anhydride groups, as evidenced by *in situ* FTIR, with the oxidation state and coordination environment around Cu evolving under the reaction’s conditions, as probed by *operando* X-ray absorption spectroscopy (XAS) measurements. This approach allowed us, for the first time, to correlate the dynamic behavior of active Cu species with the photocatalytic performance and stability for the dehydrogenation of FAc. The results confirmed the *in situ* restructuring of Cu in tandem with H_2_ evolution in the presence of FAc under solar-simulated light. Time-resolved techniques also allowed the investigation of the role of the UiO-66 framework as well as the charge carrier dynamics underlying the mechanism. Moreover, Density Functional Theory (DFT) calculations were used to highlight the impact of free carboxylate groups and their spatial distribution on Cu-coordination stability and subsequently on its possible restructuring process.

Therefore, this work provides a comprehensive mechanistic approach integrating experimental and computational insights that underscore the importance of MOF structure, host–guest synergy, *in situ* transformations, and charge carrier dynamics in driving the photocatalytic performance and offer a guiding principle for the design of next-generation light-driven catalytic systems.

## Methodology Part

### Materials

The chemical reagents and solvents used in this work were commercially obtained and utilized without the need for further purification. Formic acid (FAc) (99%) was purchased from Carlo Erba. 1,4-benzenedicarboxylic acid (BDC), 1,2,4,5-benzenetetracarboxylic acid (BTCA), zirconyl chloride octahydrate (ZrOCl_2_·8H_2_O), copper­(II) nitrate trihydrate (Cu­(NO_3_)_2_·3H_2_O), methanol (99.93% gradient grade), *N*,*N*-dimethylformamide (DMF, ACS grade), dichloromethane (DCM, ACS grade), and acetic acid (≥99.8%) were procured from Sigma-Aldrich. Commercial SiO_2_ (Ultrasil 5500 GR) was used as received from Evonik-Degussa. Basic zirconium carbonate Zr­(CO_3_)_2_·*x*H_2_O was purchased from Strem Chemicals and used without further purification.

#### Synthesis of Cu_2_O/Cu^(0)^ Nanocomposite

Cu­(NO_3_)_2_·3H_2_O (181.2 mg, 25 mmol) was dissolved in 30 mL of ethanol in a Teflon flask, to which 1.5 mL of acetic acid was slowly added. The mixture was sonicated for 5 min and then transferred into a Teflon-lined stainless-steel autoclave, filled to 80% of its capacity, and heated at 180 °C for 2 h. The resulting brick-red solid was collected, thoroughly washed several times with deionized water and ethanol, and dried at 60 °C for 12 h.

#### Synthesis of ZrO_2_

Zirconium dioxide (ZrO_2_) was prepared by calcining zirconium carbonate at 900 °C for 4 h in air. The resulting solid was allowed to cool to room temperature and then used in the photocatalytic tests.

#### Synthesis of UiO-66­(COOH)_*x*_ Samples

UiO-66­(COOH)_*x*_ were synthesized according to a previously reported procedure,^28^ with some modifications applied, using FAc as a modulator, and incorporating different ratios of 1,4-benzenedicarboxylic acid (BDC) and 1,2,4,5-benzenetetracarboxylic acid (BTCA) indicated as (a:b) in Figure 1A. More specifically, an equimolar mixture of Zr salt and organic linker(s) was dissolved in 350 equiv of DMF and 200 equiv of FAc. Then, the mixture was heated at 120 °C for 12–21 h. The heating time was tailored to obtain highly crystalline structures, with mixtures containing higher amounts of BTCA requiring a shorter heating time. Once the mixtures were cooled, MOF powders were collected by centrifugation, washed three times with DMF followed by three washings with dichloromethane (DCM), and then dried under vacuum at 120 °C overnight.

**Figure 1:**
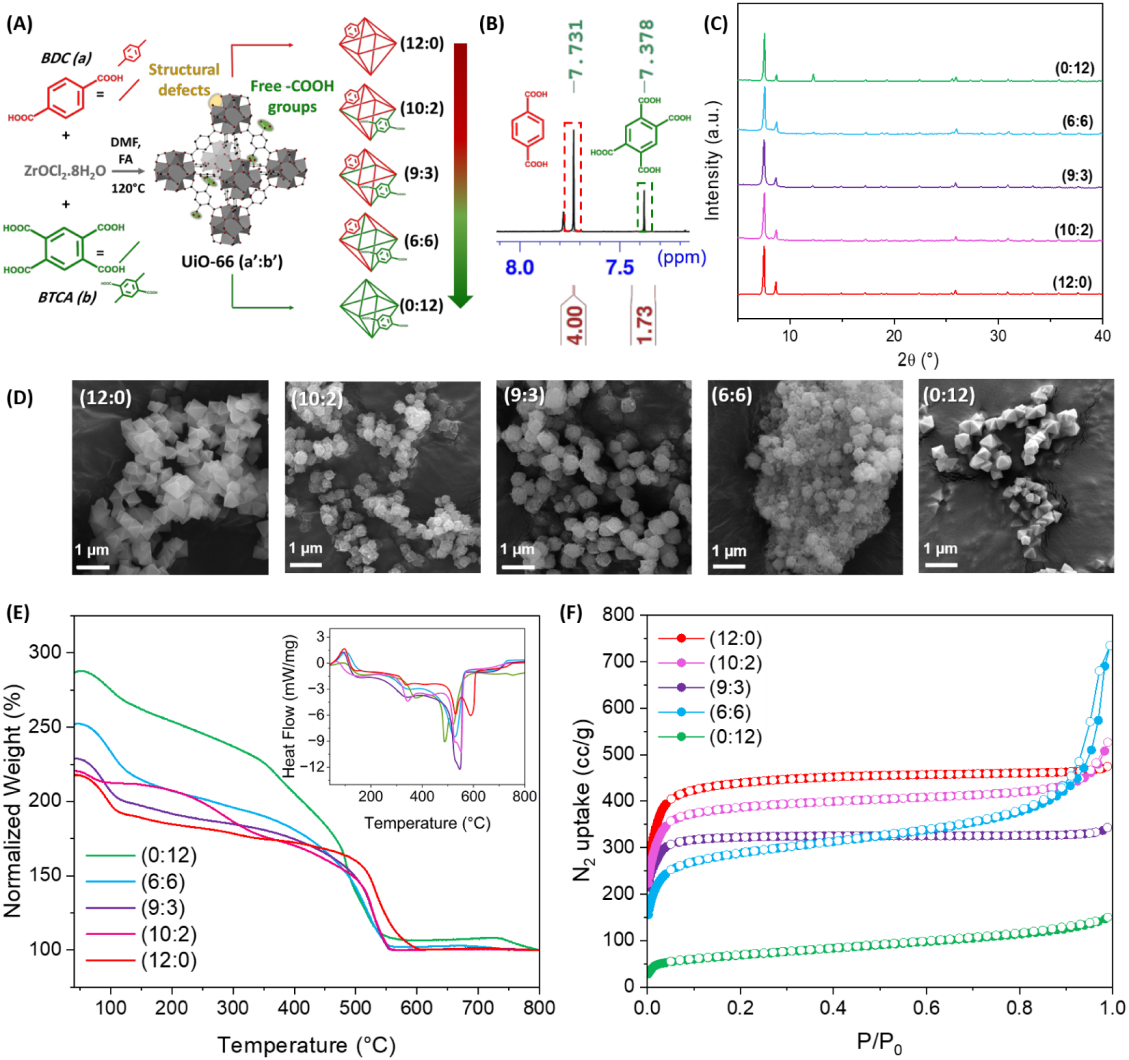
(A) Schematic illustration of the synthesized series of UiO-66­(COOH)_*x*_. (B) ^1^H NMR spectrum of the (6:6) sample, showing the characteristic peaks used for calculating the experimental linker ratios. (C) PXRD patterns, (D) SEM images, (E) normalized TGA curves and (inset) DSC profiles, and (F) N_2_ sorption isotherms of the as-synthesized UiO-66­(COOH)_*x*_, with different (a’:b’) ratios.

#### Post-synthetic Metalation Procedure

The as-synthesized UiO-66­(COOH)_*x*_ samples were post-synthetically metalated with Cu­(II), similarly to a previously reported procedure.^29^ In brief, Cu­(NO_3_)_2_·3H_2_O was dissolved in 20 mL of DMF in a 100 mL glass reagent bottle. The mass of the copper salt ranged from 50 to 150 mg depending on copper uptake by each MOF structure, such as all metalated MOFs were tuned to have 6–7.5 wt % of copper which is equivalent to a Cu/Zr atomic ratio of ∼0.2. Subsequently, 75 mg of the corresponding MOF powder was added to the mixture. Then, the mixture was sonicated, capped, and kept under stirring (300 rpm) at 100 °C for 21 h. The initial blue mixture, which turned dark brown, was centrifuged to collect the Cu-metalated MOFs which were washed sequentially with DMF and methanol over a period of 3 days to remove unreacted precursors and impurities. Finally, the material was vacuum-dried at 120 °C overnight. It should be noted that we prepared several metalated samples for each derivative and only samples with a Cu content ranging between 6 and 7.5 wt % were selected for comparison.

#### Characterization

Powder X-ray diffraction (PXRD) patterns were recorded for the freshly synthesized and metalated MOFs using a Bruker D8 Advance diffractometer with Cu Kα radiation (λ = 1.5418 Å) with 2θ ranging between 5° and 50° at an increment of 0.02°, a voltage of 40 kV, and a current of 40 mA. The Scherrer equation (*D* = *K*λ/βcosθ) was employed to calculate the crystallite size of the various MOFs synthesized where *D* corresponds to the crystallite size; K, the Scherrer constant; λ, the X-ray wavelength used in the diffraction experiment; β, full width at half-maximum (fwhm) of the diffraction peak (in radians); and θ, Bragg angle.

For scanning electron microscopy (SEM), MOF powders were dispersed on carbon tape adhered to an aluminum stub and subsequently sputtered with a 10 nm gold layer. SEM images were then taken using a Mira 3 Tescan microscope to visualize the morphology of each MOF.

Thermogravimetric analysis (TGA) and Differential Scanning Calorimetry (DSC) were performed using a TGA (SETSYS Evolution) apparatus. For each MOF, approximately 10–15 mg of powder was accurately weighed, placed in an alumina crucible, and then heated from 30 to 800 °C at a rate of 10 °C/min under air.

N_2_ sorption measurements were conducted using a Micromeritics 3Flex analyzer at 77 K. Prior to analysis, each MOF sample was degassed under nitrogen at 120 °C overnight. The surface area and pore volume of the MOF samples were determined from the adsorption–desorption isotherms.

In order to quantify the actual ratio of incorporated linkers in UiO-66­(COOH)_*x*_ derivatives, approximately 5 mg of each sample was dissolved in 1 M NaHCO_3_ solution in D_2_O via vigorous sonication, and the proton nuclear magnetic resonance (^1^H NMR) spectrum was recorded using a 500 MHz Avance III HD Bruker spectrophotometer.

The actual amount of incorporated Cu­(II) was quantified by atomic absorption spectrophotometry (AAS), whereby approximately 2 mg of each metalated MOF was accurately weighed and added to 400 μL of aqua regia under vigorous sonication for complete destruction of the framework. The amount of Cu in the solution, diluted with water, was determined using a Thermo Fisher Scientific Atomic Absorption spectrophotometer in flame mode.

Diffuse reflectance UV–visible (DR-UV–vis) measurements were conducted for optical characterization of the samples using a Cary 4000 UV–vis spectrophotometer equipped with a HARRICK Praying Mantis diffuse reflectance accessory. Spectra were recorded in the wavelength range of 200–800 nm, with an average measurement time of 0.2 s and a scan rate of 300 nm/min.

*In situ* ATR-IR analyses were performed at room temperature using an ATR cell connected to a Nicolet iS50 spectrometer equipped with a Golden Gate heating controller. Each spectrum was acquired from 4000 to 650 cm^–1^ with a resolution of 4 cm^–1^, averaging 128 scans per measurement. OMNIC software was utilized for the spectral processing and data visualization.

High-resolution TEM analyses were performed using a JEM ARM200F microscope equipped with an aberration-corrected probe and image optics. The instrument operated at 200 kV and included a cold FEG source, a CENTURIO EDX system, and a GIF Quantum spectrometer.

Solid-state photoluminescence (PL) emission spectra were recorded using an FS1000 spectrometer (Edinburgh, UK) equipped with an N-J03 as the sample holder with a front-face geometry, which also used a xenon lamp to excite the sample, which was placed between the sample holder and the emission monochromator. The time-resolved (TR) decay was collected using the same spectrometer equipped with a femtosecond laser as the excitation source (Satsuma Display AMPLITUDE) and a high-speed hybrid photodetector.

### Photocatalytic Experiments

#### Operando FTIR

For the *operando* FTIR experiments, we employed a “Sandwich” IR cell reactor (see Scheme S1) to evaluate the performance of UiO-66­(COOH)_*x*_-Cu derivatives in FAc reforming under solar-simulated irradiation at room temperature (25 °C). The catalyst, prepared as a self-supported pellet by weighing around 20 ± 1 mg of the MOF powder, was initially activated under argon at room temperature while being exposed to solar-simulated light using a 150 W Xe lamp (Hamamatsu LC8, irradiance = 71 mW/cm^2^ at 8 cm) with a high-pass filter with a cutoff of >390 nm. The reaction was subsequently conducted in the presence of 2400 ppm of FAc at a total flow rate of 25 cm^3^/min in argon. Before introducing the gas flow to the cell, we ensured that the relative concentrations of the effluent gas were stabilized; adsorption of FAc on the catalyst surface was performed in the dark prior to turning on the lamp. Following this initial setup, the composition of the gas exiting the IR reactor was analyzed simultaneously using mass spectrometry with a Pfeiffer Omnistar GSD 301 quadrupole mass spectrometer and IR spectroscopy with a ThermoNicolet NEXUS 670 FTIR spectrometer, which was equipped with an MCT detector and a spectral resolution of 4 cm^–1^, while 64 scans were accumulated. The concentration of FAc in the gas phase was calculated based on the surface area of the IR band at 1109–1101 cm^–1^ and the corresponding mass spectrometry signals for formic acid (*m*/*z* = 45 and 46). The selectivity for CO_2_ and CO was determined from the IR band areas at 2395–2282 and 2140–2020 cm^–1^, respectively. The amounts of H_2_ were measured using its mass spectrometry signal at *m*/*z* = 2, after adjusting for contributions from water. FAc conversion (expressed either as a percentage or in millimoles per gram of photocatalyst or per irradiated surface area) and selectivity were calculated at steady state based on calibration curves for the reaction various products. It is important to note that approximately 1.6 cm^2^ (around 80% of the total surface area of 2 cm^2^) of the pellet’s surface was irradiated, while a toroidal portion was shadowed by the metallic holder.

#### Operando Raman Spectroscopy

Raman spectra were recorded with a Horiba Labram HR Evolution Raman microscope with laser excitation at 532 nm. The sample with the highest activity (about 30 mg) was placed in a Linkam CCR-1000 heating cell with a quartz window. The cell was placed under a total flow rate of 25 cm^3^·min^–1^ of argon in the presence of 2400 ppm FAc. Spectra were recorded serially for 30–60 s each at ca. 1.3 mW on the sample. In order to fully illuminate the sample and to avoid perturbation of the Raman spectra in the presence of light, the irradiation of the cell was carried out while the cell was removed from the microscope. Then, right after irradiation, the cell (kept under gas flow) was placed under a microscope for Raman spectral acquisition. Gas products were continuously monitored with a mass spectrometer at the exhaust of the cell.

#### X-ray Absorption Spectroscopy (XANES and XAFS)

The *operando* quick-XAS experiments at Cu (8979 eV) and Zr (17 998 eV) K-edges were performed at the ROCK beamline (SOLEIL synchrotron).^30^ The monochromatic beam was obtained using Si (111) quick-EXAFS channel-cut monochromators aligned at 12.3° and 6.1° and oscillation amplitudes of 3.9° and 0.6° for Cu and Zr K-edges, respectively. The monochromators were calibrated by setting the first inflection point of a Cu and a Zr metallic foil to 8979 and 17 998 eV, respectively. The harmonic rejection at Cu and Zr K-edges was ensured using, respectively, B_4_C- and Pd-coated mirrors that were installed before and after the monochromator at a grazing incidence of 2.8 mrad with respect to the pink and monochromatic beam. The oscillation frequency of the monochromators was set to 2 Hz, allowing one XAS spectrum to be recorded in 250 ms. Measurements were performed in transmission mode using three ionization chambers (Ohyo Koken) filled with N_2_ and a mixture of N_2_/Ar (50/50) for Cu and Zr K-edges, respectively. The two first ionization chambers were used to measure the spectra of the sample, and a copper and zirconium foil was placed on the third to be used as a reference for further energy calibration and alignment. The sample was prepared, and the photocatalytic reaction was performed following the same procedure as for *operando* FTIR, except for the cell which was a reactor developed at the ROCK beamline and suitable for *operando* quick-XAS under UV–visible irradiation.^31^ The outcoming gases from the cell were analyzed by using a Pfeiffer mass spectrometer.

Quick-XAS data were extracted, aligned in energy, and normalized using the Python graphical interfaces (“extract_gui” and “normal_gui”) developed at the ROCK beamline.^32^ The XAS data were analyzed using the FASTOSH software.^33,34^ The EXAFS signals were extracted using the Autobk algorithm (Rbkg = 1, k-weight = 3). The corresponding Fourier transforms were calculated over the ranges 3–12 and 3–14 Å^–1^ (apodization window = Kaiser, d*k* = 1) for Cu and Zr K-edges, respectively. Cu K-edge *operando* quick-XAS datasets were analyzed using a chemometrics approach combining principal component analysis (PCA) followed by multivariate curve resolution with alternating least-squares (MCR-ALS). This methodology has been widely used and described to analyze quick-XAS datasets in many applications.^35–38^ The PCA and MCR-ALS analysis were performed using the MCR-ALS GUI 2.0 toolbox available for Matlab software.^39^ EXAFS fitting was performed using the Artemis interface^40^ to IFEFFIT using least-squares refinements in the range of 1–3.8 Å. The theoretical backscattering paths were calculated using FEFF6 from CuO, Cu_2_O, and Cu.

#### Computational Methods

The geometry optimization of all MOF structure models discussed above was achieved using periodic DFT calculations carried out using the Vienna *Ab Initio* Simulation Package (VASP)^41^ with the Perdew–Burke–Ernzerhof (PBE)^42^ functional incorporating Grimme’s DFT-D3 corrections to account for van der Waals interactions.^43^ The convergence criteria for electronic energy and atomic forces were set to 10^–5^ eV and 0.01 eV Å^–1^, respectively. A plane-wave cutoff energy of 520 eV was used consistently throughout the calculations. The Brillouin zone was sampled using a 1 × 1 × 1 k-point mesh, generated via the Monkhorst–Pack scheme.^44^ The free energies of the reactions were calculated using the computational approach model proposed by Nørskov and colleagues.^45^ For each elementary step, the Gibbs free energy change (Δ*G*) was calculated as follows:


$$\Delta G=\Delta E_{DFT}+\Delta E_{ZPE}-T\Delta S$$

where Δ*E*_DFT_ is the electronic energy from DFT, Δ*E*_ZPE_ is the correction for the zero-point energy, and Δ*S* is the change in entropy at *T* = 298.15 K.

## Results and Discussion

### Characterization of UiO-66­(COOH)_*x*_ Derivatives

UiO-66­(COOH)_*x*_ derivatives were synthesized using different molar ratios of BDC and BTCA, denoted as (a:b) and (a’:b’) for starting and experimental ratios of (BDC:BTCA), respectively, with a’ + b’ = 12 corresponding to the theoretical number of linkers connecting each Zr_6_O_4_(OH)_4_ cluster as illustrated in Figure 1A. (a’:b’) were determined from the ^1^H NMR spectra of the samples, as illustrated in Figures 1B and S1. Zooming into the 7–8.5 ppm region, the singlet at around δ = 7.4 ppm is attributed to the two equivalent hydrogen atoms of the BTCA benzene ring, while the singlet at δ = 7.7 ppm corresponds to the four equivalent benzene ring hydrogens of BDC. The amount of each linker in each sample was determined by considering the integration of both peaks, and the approximated percentage of BTCA (estimated error around 10%) is included in Table 1. Lastly, the singlet depicted at δ = 8.3 ppm in all samples is attributed to residual FAc molecules trapped inside the pores. PXRD patterns of the five different samples (Figure 1C) exhibit distinct and well-defined peaks, consistent with the pattern of pristine UiO-66, validating the crystallinity and phase purity of the samples. SEM microimages of the (12:0) and (0:12) samples show uniform morphologies (Figure 1D), demonstrating a perfect, smooth, octahedral shape. However, samples with (10:2), (9:3), and (6:6) ratios illustrated a rough surface and nonsharp edges of their octahedra. The average particle size of each sample estimated from its corresponding SEM images is listed in Table 1. While (12:0) and (0:12) have the highest average particle sizes, samples with (10:2), (9:3), and (6:6) exhibit smaller crystal sizes ranging from 335 to 360 nm, which may be attributed to the linkers’ competition to bind the Zr-nodes that hinders crystal growth and/or due to differences in ligand relative solubilities.^46^ This is in line with the trend of crystallite size estimated from Scherrer’s equation (*D* = *K*λ/βcosθ) applied on the corresponding PXRD patterns of these cubic samples (Table 1). Thermal stability, assessed using TGA-DSC analysis, was subsequently employed to estimate the defect concentration in each MOF structure. The DSC profiles of all synthesized MOFs (inset of Figure 1E) show an endothermic peak between 50 and 150 °C, attributed to the loss of trapped solvent molecules. The complete destruction of the crystalline frameworks is marked by a sharp exothermic loss of organic linkers above 400 °C. A closer comparison of this main decomposition between samples demonstrates that (12:0), synthesized from pure BDC, is the most thermally stable, as expected, while the pure BTCA form (0:12) is the least stable, undergoing a major decomposition starting from lower temperatures, as reported before by some of us.^47^ The stability of UiO-66­(COOH)_*x*_, incorporating both linkers, falls in between (Figure 1E), with the MOF structures becoming less thermally stable when the actual amount of BTCA in the structure increases. A similar trend was in fact reported for partially and fully functionalized UiO-66 systems, in which additional functional groups are shown to reduce the thermal stability of the framework because of the steric hindrance they induce, thus weakening the metal-linker interactions.^48^ Defect numbers, defined as the number of missing linkers per Zr-cluster in each MOF sample, were then estimated from the corresponding normalized thermograms based on a previously reported method,^49^ and the calculation details are included in the Supporting Information file, with the resultant defect number for each MOF prepared being included in Table 1. It should be noted here that all UiO-66­(COOH)_*x*_ were synthesized using the same amount of FAc modulator, and the close defect numbers obtained permit the exclusion of any significant impact of this factor on the photocatalytic behavior of the corresponding samples. BET surface areas and pore volumes of the five prepared MOFs were compared based on their N_2_ sorption isotherms shown in Figure 1F. These are in line with the microporous nature of the synthesized MOFs except for the (6:6) sample having a type IV behavior, with a H4 hysteresis suggesting the coexistence of micro- and mesopores in this MOF. This is further validated by the corresponding pore size distributions (Figure S2) that demonstrate the presence of micropores in all UiO-66­(COOH)_*x*_ samples, with a size ranging between 1.6 and 1.9 nm. Additionally, (6:6) sample is characterized by the presence of mesopores ≥3 nm, similarly to previously reported hierarchically porous UiO-66 systems.^50^ A similar observation was reported for a mixed-linker version of HKUST-1, incorporating 50% of BTCA, as it displayed a mesoporous character unlike the microporous monolinker framework.^51^ This can be possibly attributed to the small particle size and defective nature of the (6:6) sample as missing cluster defects might have been generated upon the strong competition between BDC and BTCA incorporated in equal amounts. This is further supported by the hypothesis that FAc occupies the resulting voids. While TGA analysis shows similar values for missing linker defects across all UiO-66­(COOH)_*x*_ derivatives, it does not account for missing cluster defects. Therefore, the ^1^H NMR-derived FAc/linker ratio serves as a complementary method for identifying such defects, providing a more complete picture of the defect structure. In fact, the FAc/linker ratio, determined by ^1^H NMR (Figure S1), is the highest for the (6:6) sample, reinforcing this interpretation. The BET surface area, tabulated in Table 1, decreases from 1810 to 239 m^2^/g when increasing the BTCA content from 0 to 12. The increasing number of free COOH groups hinders access to the framework internal void. Moreover, the pure BDC sample (12:0) has a total pore volume of 0.74 cm^3^/g which is close but lower than that of (10:2) sample having excess BDC (0.81 cm^3^/g), possibly because of the slight difference in defect numbers. As the BTCA content increases further, the pore volume decreases, reaching 0.23 cm^3^/g for the pure BTCA-sample (0:12). The (6:6) structure is an exception, demonstrating the highest total pore volume of 1.1 cm^3^/g due to its mesoporous character and high defect content having both missing linker and missing cluster defects, as demonstrated by TGA and ^1^H NMR analyses. Once synthesized, all UiO-66­(COOH)_*x*_ were postmetalated with Cu, as described in the experimental section, yielding structures termed as UiO-66­(COOH)_*x*_-Cu. The amount of Cu within the samples was quantified using AAS, and the values are presented in Table 1. For this study, only samples with similar Cu content (6–7.5 wt %) were selected to ensure comparable conditions. The incorporation of Cu in the MOF structure was also confirmed by UV–vis analysis (Figure S3) whereby the UV–vis absorption spectra showed the ligand-to-metal charge transfer (LMCT) transitions for Cu^(II)^-linker in the 200–400 nm range.^52^ PXRD patterns recorded after metalation (Figure S4) demonstrated that the MOFs maintained their structural integrity and validated the incorporation of cationic Cu into the crystalline MOFs. Cu K-edge XAS analysis revealed that the incorporated Cu is predominantly in the +2-oxidation state with only minor contributions from Cu^(I)^ present as small clusters with neighboring Cu^(II)^ centers (details provided in a later section). Consistently, EXAFS fitting showed that in the (0:12)-Cu, the pristine Cu^(II)^ centers are coordinated to oxygen atoms in a distorted octahedral environment (see Supporting Information Table S4 for details). N_2_ sorption was also conducted on the metalated samples, and a decrease in all MOFs N_2_ sorption uptake and pore volumes is noticed, as shown in Figure S5. Therefore, the structural and physical properties of the samples and the incorporation of similar Cu content are in line with the experimental target and permit investigating the effect of the BDC:BTCA ratio on the photocatalytic performance, without excluding the possible effect of the specific surface of the corresponding samples.

**Table 1:** Textural Properties of UiO-66­(COOH)_*x*_ and their Corresponding Copper Content^a^

Sample label	BTCA linkers’ % from ^1^H NMR	BET surface area (m^2^/g) ±10%	Pore volume (cm^3^/g) ±5%	Defect number	Crystallite size (nm)	Particle size (nm)	Cu content (wt %)
(0:12)	100	239	0.23	2.7	58	410	6.3
(6:6)	50	1106	1.10	2.7	38	339	7.5
(9:3)	20	1353	0.52	2.6	37	357	6.4
(10:2)	10	1575	0.81	2.6	41	336	7.3
(12:0)	0	1810	0.74	2.5	56	446	6.2

a Cu content in the corresponding UiO-66­(COOH)_*x*_-Cu samples.

### Operando FTIR Study

After the successful synthesis of the UiO-66­(COOH)_*x*_-Cu series and the selection of MOF samples with comparable copper content (6 to 7.5 wt %), all these materials were evaluated for FAc dehydrogenation under solar-simulated light using an *operando* FTIR reactor (Scheme S1) in standardized conditions. These combined measurements allow monitoring the evolution of the photocatalyst surface as well as simultaneous determination of the FAc conversion and the selectivity of the reaction. The evolution of H_2_ production, recorded over 800 min for the five samples, demonstrates that no deactivation phenomenon is observed during the reaction (Figure S6). As a result, the activity at steady state conditions was determined and the results are reported in Figure 2A. For instance, FAc dehydrogenation reached a maximum conversion rate of 5 mmol·g^–1^·h^–1^ over (0:12)-Cu sample and then decreased gradually with the decrease of the concentration of BTCA in the sample to reach 4.4, 2.8, 1.2, and 0.6 mmol·g^–1^·h^–1^ for (6:6)-Cu, (9:3)-Cu, (10:2)-Cu, and (12:0)-Cu samples, respectively. The corresponding TOF values obtained based on the total amount of Cu as active sites along with the apparent quantum yield percentage (Φ_H2_) under simulated solar light were calculated based on the procedure and equation present in the Supporting Information file, for the series of samples (Table S1). Among the series, (0:12)-Cu exhibited the highest TOF value of 1.41 s^–1^ (Table 2) and apparent quantum efficiency of 11.5%, which is relatively high for vapor-/solid-phase heterogeneous catalysts. The (6:6)-Cu sample followed closely, recording a TOF of 1.03 s^–1^ and an efficiency of 10.1%, confirming that this composition also provides highly effective catalytic sites. In contrast, (9:3)-Cu showed a significantly lower TOF (0.7 s^–1^) and quantum efficiency (6.4%), suggesting less efficient utilization of its active sites. The (10:2)-Cu and (12:0)-Cu led to a marked decrease in both TOF and apparent activity, with quantum efficiencies dropping below 3%. These results highlight the importance of Cu coordination to the free carboxylates and the formation of active sites, which impact the activity and the efficiency of the system. It should also be noted that Cu-free samples showed no activity under similar reaction conditions. The FTIR surface analysis of the samples revealed no significant changes, neither during the activation under irradiation with Ar at room temperature nor after FAc adsorption in dark. However, during the FAc dehydrogenation reaction under solar-simulated light, a temporal evolution of new surface vibrational bands with various intensities, within the 1900–1700 cm^–1^ range, was observed for all samples as illustrated in Figures 2B and S7. The new bands, already identified in our previous work,^28^ are characteristic of anhydride formation. A small amount of anhydride groups was also detected in the (12:0)-Cu sample, even though it was synthesized without BTCA linkers. However, the presence of free carboxylate groups originating from the missing cluster defects cannot be ruled out. These defect-associated COOH groups can, therefore, undergo condensation simultaneously with Cu restructuring under photocatalytic conditions, leading to the formation of bridging anhydride moieties, albeit in smaller quantities compared with BTCA functionalized samples (Figure S8). Therefore, the weak anhydride signal likely originates from these defect sites that persist within the framework. This limited anhydride formation can still facilitate some degree of Cu species restructuring, which in turn accounts for this limited photocatalytic activity. The formation of the anhydride should impact the coordination of Cu within the UiO-66­(COOH)_*x*_-Cu frameworks, accompanied by a possible variation of their oxidation states to compensate the counter charge. This point is further discussed in the following section. In order to estimate the relative amount of anhydride formed throughout the reaction, we focused on the well-defined band centered at 1855 cm^–1^ (Figures 2C and S9). This band showed a significant increase in intensity, directly correlating with the increase in BTCA concentration in the sample, highlighting its impact on the anhydride formation (Figures 2D and S10). This process involves the dehydration of the bis-carboxylate groups of the framework, indicating changes in the Cu coordination. Notably, a linear correlation was identified between the specific conversion rate (in mmol·g^–1^·h^–1^) of the samples and the quantity of anhydride formed, as shown in Figure 2D. However, when referring to the surface after Cu incorporation, our analysis revealed an exponential correlation between the conversion rate (in mmol·m^–2^_cat_·h^–1^) and the anhydride content in the samples, indicating that the anhydride formation is not the only factor affecting the activity. This suggests that the variations in the photocatalytic performance are primarily due to differences in Cu speciation and restructuring behavior rather than variations in accessible surface area alone (Figure 2D). Furthermore, the number of -COOH groups to which Cu is coordinated is not the sole determining factor; their quantity and spatial distribution also play a crucial role, as they can influence both the formation rate and stability of anhydrides. These effects will be further elucidated in the modeling section. On the other hand, the variation of the relative rate of anhydride formation against the specific surface of the samples did not reveal any specific relationship, where the (9:3)-Cu sample exhibited the highest rate (Figures 2D (inset) and S10). Consequently, while the anhydride formation plays a crucial role behind the sample activity, it potentially alters the Cu coordination within the MOF structure. Cu species also undergo some changes during the reaction as confirmed using *operando* Raman spectroscopy (Figure S11). For instance, for the pristine (0:12)-Cu sample, and prior to activation, bands can be observed at 1185, 1438, and 1606 cm^–1^ due to the carboxylate ligand, while bands at ca. 295, 340, and 600 cm^–1^ are mainly assigned to copper species. These latter bands are consistent with the formation of Cu–O_*x*_ clusters (with 0.5 ≤ *x* ≤ 1).^53,54^ During the reaction, the mass spectrometer evidenced the formation of CO_2_ and H_2_ upon irradiation. On the *operando* Raman spectra, the ligand bands remained visible. By contrast, the overall intensity of the spectra decreased, and the bands due to Cu–O_*x*_ species vanished. In addition, a periodic perturbation of the spectrum was observed which might be due to the plasmonic behavior of metallic Cu centers. This would suggest that Cu–O_*x*_ species are undergoing further reduction and that metallic Cu^(0)^ clusters are possibly formed. After the reaction, and withdrawal of the catalyst from the cell at room temperature, the spectrum of the recovered catalyst showed again the presence of Cu–O_*x*_, but peaks at 213, 422, and 630 cm^–1^ were also observed. These bands are generally attributed to Cu_2_O,^54,55^ i.e., Cu^(I)^. It should be recalled that during the reaction under *operando* conditions, only the upper part of the catalyst was subjected to light irradiation. Therefore, upon removal of the recovered powder, the initial Cu–O_*x*_ is logically reobserved. However, the additional presence of Cu_2_O could be due to changes in the copper oxidation state during the photocatalytic reaction. This change in the nature of the Cu species can be another factor governing the activity of the catalysts. Therefore, further investigation on the evolution of the Cu coordination and oxidation state during the reaction is necessary.

**Figure 2:**
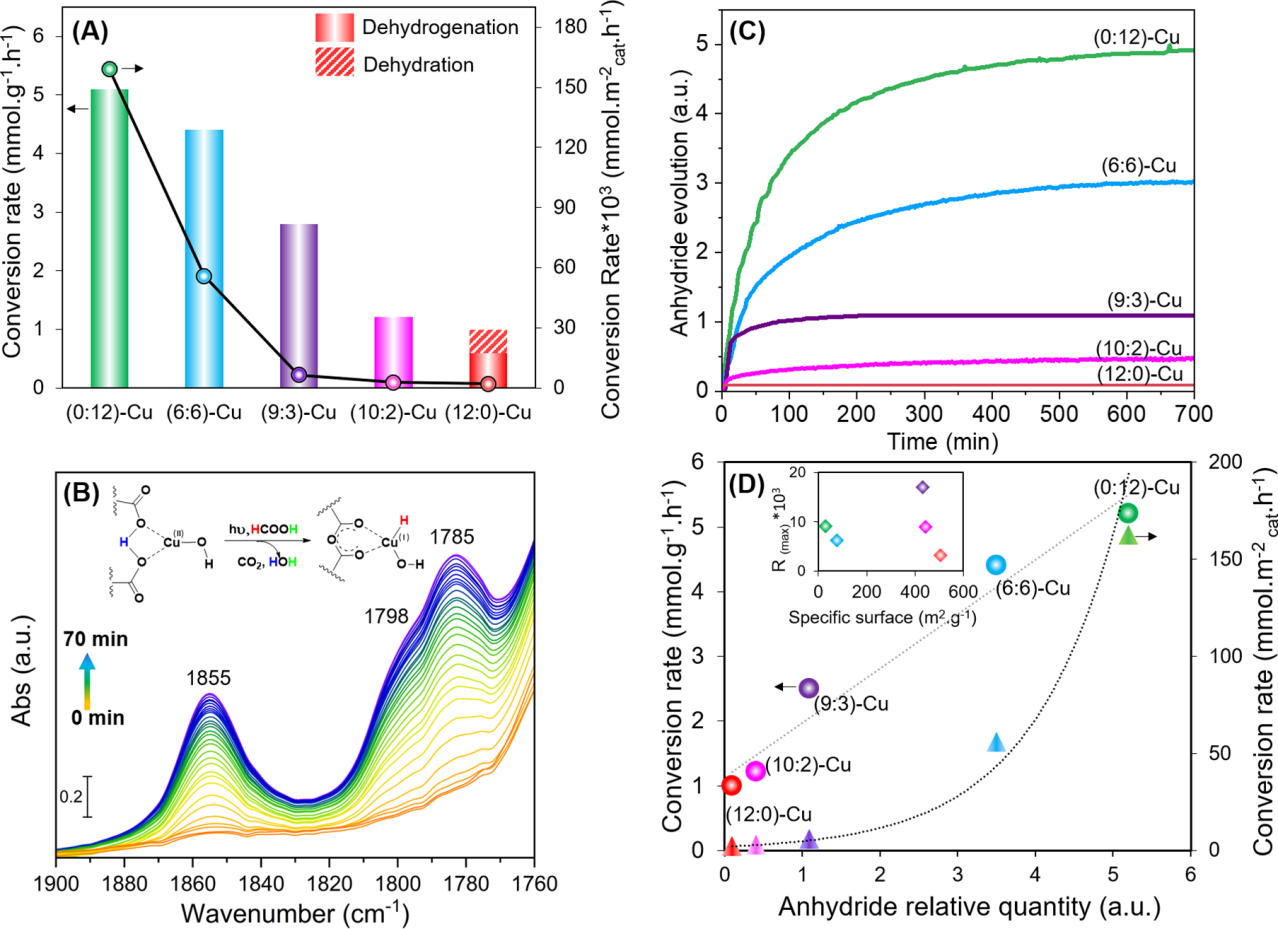
(A) Conversion rates (in mmol·g^–1^·h^–1^ and mmol·m^–2^_cat_·h^–1^) of various samples during simulated-solar-driven photocatalytic reforming of FAc at steady state. (B) Evolution, versus reaction time, of the IR surface spectra of the (0:12)-Cu sample during the FAc dehydrogenation in the 1900–1700 cm^–1^ region corresponding to the anhydride vibrational region. (C) Evolution, versus reaction time, of the anhydride band area (centered at 1855 cm^–1^) on the various sample surfaces . (D) Variation of the anhydride band area at steady state as a function of the conversion rates (in mmol·g^–1^·h^–1^ and mmol·m^–2^_cat_·h^–1^), inset: variation of the anhydride formation rate versus the specific surface area of the various samples after Cu incorporation.

**Table 2:** Results of the FAc Dehydrogenation with Various Photocatalysts^a^

Sample	H_2_ evolution rate (mmol. g^–1^·h^–1^)	H_2_ evolution rate (mmol·h^–1^·m^–2^)^b^	TOF (s^–1^)^c^	Φ_H2(visible‑light)_ ^d^ (%)
(0:12)-Cu	5.0	630	1.41	11.51
(6:6)-Cu	4.4	550	1.03	10.1
(9:3)-Cu	2.8	350	0.77	6.4
(10:2)-Cu	1.2	150	0.29	2.78
(12:0)-Cu	0.6	80	0.17	1.38

a Reaction conditions: [FAc] = 2400 ppm (0.24%) in Ar; total flow = 25 cm^3^·min^–1^; *T* = 25 °C; Xe-lamp = 150 W with visible light pass filter (λ > 390 nm); irradiance = 71 mW·cm^–2^; *m*_cat_ = 20 mg (self-supported pellet with a surface area of 1.6 cm^2^).

b H_2_ evolution rate in mmol per h per m^2^ of irradiated surface.

c Apparent TOF calculatedusing the number of Cu atoms as active sites.

d Apparent quantum yield.

### *Ex Situ* Characterization of the UiO-66­(COOH)_*x*_-Cu Samples after FAc Dehydrogenation Reaction

To further investigate the structural and electronic changes of the catalyst before and after reaction, we conducted post-reaction analyses using UV–vis, PXRD, ATR-IR, and HRTEM. UV–vis spectra of the samples were measured pre- and post-reaction (Figure 3A). Notably, a broad absorption band attributed to Cu oxide appears in the visible region (420–485 nm), along with a band at 580–590 nm associated with the plasmonic band of Cu^(0)^, indicating a possible reduction of Cu oxidation state.^56^ Certain samples, such as (9:3)-Cu and (10:2)-Cu, also exhibit a band in the 685–800 nm region, likely due to the formation Cu­(OH)_2_ ^57^ or Cu­(OOCH)_*x*_ formed upon the reaction with the residual FAc. For instance, Cu­(OH)_2_ formation may occur via two possible ways: (i) reaction of uncoordinated Cu^(II)^ species or (ii) the reaction of the outer Cu_2_O surface layer with atmospheric humidity, wherein the bulk Cu_2_O remains nonaffected. This moisture-induced instability is further supported by the observed color change from dark brown to blue upon atmospheric exposure of some samples (especially prepared with low BTCA concentration), indicating alterations in either the oxidation state or the coordination environment of Cu species that occur outside the oxygen- and/or moisture-free conditions of the catalytic reaction. This indicates the importance of the *in situ* generation and stabilization of active Cu sites under controlled reaction conditions within the UiO-66­(COOH)_*x*_ framework that acts as a stabilizer for the restructured Cu species. PXRD analysis was conducted to further assess the structural stability of the samples before and after the reaction (Figure 3B). The results reveal that all samples, except the (12:0)-Cu, maintain their crystallinity after the reaction, displaying the characteristic diffraction peaks of the UiO-66 framework. This also highlights that the sensitivity of the samples upon exposure to air or residual FAc in the pores did not affect the local structure of the MOF. Indeed, the (12:0)-Cu sample without BTCA linker exhibits clear signs of post-reaction structural transformation. Specifically, this sample shows a broad hump in the PXRD pattern, consistent with the partial amorphization of the framework under photocatalytic conditions. Furthermore, a notable diffraction peak at around 2θ ≈ 43° is observed after reaction. This peak is attributed to the Cu^(0)^ nanoparticles formed during the reaction and is characteristic of the (111) plane of Cu^(0)^.^58^ In the absence of anhydride bridges, the *in situ* restructured Cu species are insufficiently stabilized within the framework, making them prone to sintering and agglomeration. This structural instability likely accounts for the presence of a Cu^(0)^ peak in this sample. The presence of Cu^(0)^ was not observed in other samples, where the anhydride-bridged MOF scaffold effectively maintained and permitted good dispersion of the restructured Cu species. Notably, when a similar photocatalytic experiment was conducted by some of us using a Cu_2_O/Cu^(0)^ catalyst containing the same active binary Cu system, the presence of metallic Cu was also confirmed by TEM and XRD analyses, suggesting analogous sintering and agglomeration effects due to the absence of a stabilizing framework.^26^ Thus, these observations underscore one of the critical roles of the bridged anhydrides: they stabilize the framework structure during the reaction and prevent uncontrolled Cu aggregation into metallic nanoparticles, thus maintaining both crystallinity and catalytic performance. Conversely, the instability of the (12:0)-Cu sample, leading to the presence of Cu^(0)^ under our reaction conditions, may also play a key role in determining the selectivity of the reaction as witnessed from Figure 2A. The (12:0)-Cu sample indeed exhibits a 60% dehydrogenation selectivity while all the other samples are highly selective (100% selectivity toward H_2_). In the (12:0)-Cu sample, the Cu cations are expected to occupy the vicinity of the defect sites of Zr clusters (Figure S8). Hence, the loss of coordination between the Zr-oxo clusters and the BDC linkers might become more easy, causing instability. Thus, more defect sites are generated, increasing the Lewis acidity and probably favoring the dehydration pathway. This was also highlighted in previous reports when using Nb_2_O_5_/Cu_2_O and TiO_2_/Cu_2_O photocatalysts, where the dehydration pathway was favored due to the increased Lewis acidity.^26^ To further validate this hypothesis, reference reactions were carried out on Cu_2_O:SiO_2_ and ZrO_2_:SiO_2_. In addition, a Cu_2_O:ZrO_2_:SiO_2_ sample having a similar ratio of Cu to Zr (ratio = 0.2), to mimic the Cu:Zr ratio used in the (12:0)-Cu framework, was also investigated and the results are illustrated in Figure S13. For instance, Cu_2_O exhibits 100% selectivity toward the dehydrogenation pathway, although with a limited conversion rate of only 0.4 mmol·g^–1^·h^–1^, consistent with previous literature reports.^26^ While ZrO_2_:SiO_2_ displayed no detectable activity within the sensitivity limits of our instrumentation, the combination of Cu_2_O and ZrO_2_ (Cu_2_O:ZrO_2_) exhibited a notable activity of 2 mmol·g^–1^·h^–1^. This composite demonstrated a significant decrease in dehydrogenation selectivity, reaching 66%, indicating that the presence of ZrO_2_ promoted the dehydration pathway to some extent. The activity of the Cu_2_O:ZrO_2_ sample was not equal to the sum of the activity of the bare Cu_2_O and ZrO_2_. Thus, these findings underscore a potential synergistic effect between Cu_2_O and ZrO_2_ and a possible charge transfer, which enhances the photocatalytic performance. This shift in the reaction pathway highlights the role of the ZrO_2_ component, where the higher Lewis acidity of non-coordinated Zr clusters in the case of (12:0)-Cu promotes dehydration over dehydrogenation. Therefore, a possible synergistic behavior occurs between the Zr-oxo clusters and restructured Cu in the UiO-66­(COOH)_2_ framework enhancing its photocatalytic activity toward dehydrogenation. Thus, beyond the protective function of the anhydride bridges that stabilize the copper species, prevent sintering, and preserve the coordination environment of the Zr nodes, thereby neutralizing their acidity and suppressing the dehydration pathway, the UiO-66­(COOH)_2_ framework may also play a key role in facilitating charge transfer from the Zr-oxo clusters to the restructured Cu sites, enhancing the photocatalytic activity.

**Figure 3:**
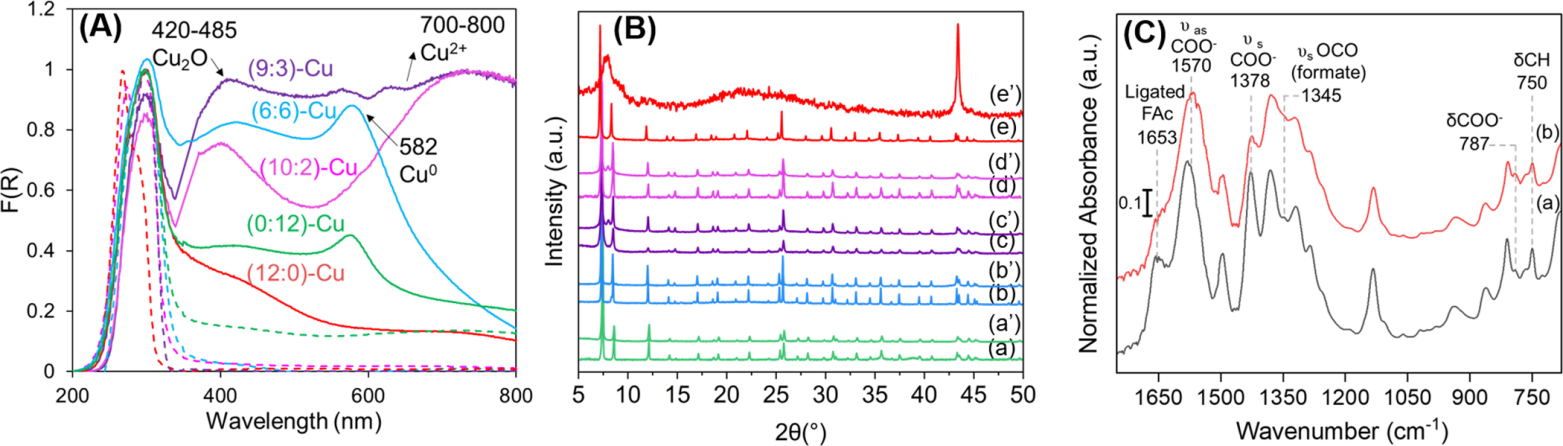
(A) UV–vis absorption spectra of the various samples before (dashed line) and after (full line) reaction. (B) The PXRD patterns of various samples before (a, b, c, d, e) and after (a’, b’, c’, d’, e’) reaction, for (0:12)-Cu, (6:6)-Cu, (9:3)-Cu, (10:2)-Cu, and (12:0)-Cu, respectively. (C) ATR-IR spectra of the (0:12)-Cu sample before (a) and after (b) reaction.

Further insights into surface modifications of the catalysts were obtained through ATR-IR analysis, which enabled a more detailed examination of structural bands within the UiO-66 framework that were saturated in the FTIR transmission mode. A representative sample (0:12)-Cu was selected for the ATR analysis. Similar results were also obtained with the (6:6)-Cu sample as shown in Figure S14. The ATR spectra (Figure 3C) of the (0:12)-Cu sample exhibit the characteristic bands of the UiO-66 framework including two prominent peaks at 1570 and 1378 cm^–1^, corresponding to the asymmetric and symmetric stretching vibrations of the carboxylate groups of the terephthalate linker, observed both before and after the reaction.^59,60^ Additional spectral features reflect modifications due to reaction intermediates on the catalyst surface. For instance, a new peak at 1345 cm^–1^ appeared after the reaction, attributed to the asymmetric stretching of monodentate formate bound to Cu, a key intermediate in HCOOH dehydrogenation to CO_2_ and H_2_.^26,61^ The intensity of the 787 cm^–1^ peak, associated with the bending vibration of the coordinated −COO^–^ groups, also increased after the reaction. This aligns with FTIR data showing the formation of an anhydride bridge during photocatalysis, likely due to the condensation of the free carboxylate groups during the reaction. Furthermore, an increase in the peak intensity at 750 cm^–1^, corresponding to C–H bending vibrations, suggests an increase in available sites for HCOOH adsorption on the MOF surface, enhancing the catalytic efficiency.^61^

TEM analysis has also provided valuable insights into coordination modifications occurring during the reaction, particularly through the formation of copper oxide clusters within (0:12)-Cu and (6:6)-Cu used as representative samples. High-angle annular dark-field scanning TEM (HAADF-STEM) images, displayed in Figure 4A,B, reveal bright contrast areas corresponding to these copper clusters. For instance, the (0:12)-Cu sample showed a higher amount of monodispersed Cu_2_O particles which can be indexed to a cubic Cu_2_O phase (space group *Pn3̅m*, lattice parameter *a* = 0.426 nm; ICSD 172174), with a larger particle size (2–3 nm) in agreement with our previous work,^28^ while the (6:6)-Cu sample showed polydispersed Cu_2_O particles with two distinct sizes indicating variations in particle growth and stability depending on the initial sample composition. Particularly, the selected-area electron diffraction (SAED) pattern from individual (6:6)-Cu clusters post-reaction shows distinct diffraction rings (bottom right inset Figure 4C), consistent with the cubic Cu_2_O structure (*Pn*3̅*m* (224), *a* = 0.426 nm; ICSD 172174). The corresponding HRTEM image (Figure 4C) confirms the presence of very small Cu_2_O nanoparticles (around 1 nm size) within the MOF structure. Only a few large Cu_2_O nanoparticles (Figure 4D) are observed on the surface of the MOF crystal in the (6:6)-Cu sample. EDX-STEM elemental maps (Figure S15) clearly show a homogeneous distribution of Cu within the MOF crystal. All these features suggest the presence of Cu-oxo species, formed via Cu^(II)^ reduction during the reaction and stabilized by strong interactions with anhydrate groups in the framework. Notably, no Cu^(0)^ nanoparticles were observed, in contrast to the UV–visible absorption data. This discrepancy may be attributed to the high dispersion of Cu^(0)^ species, as supported by PXRD results, which confirmed the presence of large metallic Cu crystals only in the (12:0)-Cu sample, where the absence of anhydride bridges promotes Cu sintering. The absence of detectable Cu^(0)^ on the other samples may be attributed to its air sensitivity or possible amorphous (noncrystalline) nature. To minimize the risk of beam-induced reduction of Cu_2_O to Cu^(0)^ during imaging, all measurements were carried out under a low electron beam voltage. This precaution was crucial to ensure that the observed Cu species accurately reflect the truthful post-reaction state of the sample, rather than artifacts introduced by electron-beam exposure.

**Figure 4:**
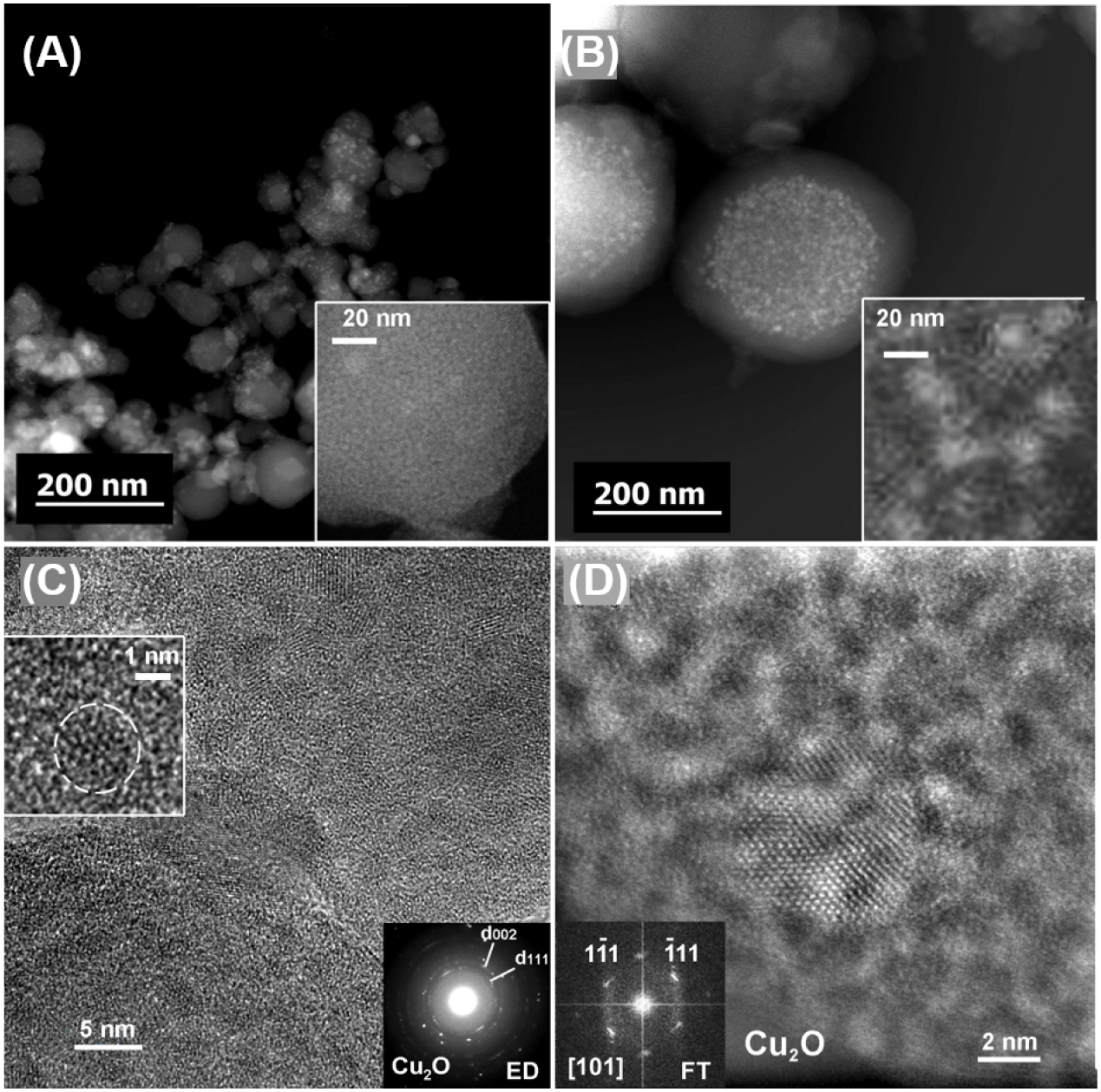
(A) Low magnification HAADF-STEM image overview of (6:6)-Cu and (B) (0:12)-Cu samples after reaction. Magnified images of selected areas are given as insets. (C) Bright-field HRTEM image of the (6:6)-Cu sample together with the corresponding ring ED pattern. Two distinguished rings (d002 and d111) of Cu_2_O structure are depicted. Inset: magnified image shows Cu_2_O small nanoparticle (around 1 nm) marked by a white circle. (D) [101] HAADF-STEM image of a large (around 8 nm) Cu_2_O nanoparticle and the corresponding FT pattern in the (6:6) sample.

### Investigation of the Cu Dynamic Behavior during Photocatalytic Dehydrogenation of FAc by Operando XAS

To build on these insights and gain a more detailed understanding of the evolution of Cu coordination and oxidation state within the UiO-66­(COOH)_*x*_-Cu frameworks during the reaction, we performed *operando* XAS, under similar conditions used above for *operando* FTIR studies. The catalyst was prepared as a self-supported pellet, embedded in the reactor developed at the ROCK beamline at SOLEIL-synchrotron.^31^ For time restrictions, a set of experiments was carried out over the (0:12)-Cu, (6:6)-Cu, and (9:3)-Cu selected samples. Both Zr and Cu K-edge XANES and EXAFS analyses were performed to investigate the modification of both metals during the various steps of the reaction. For the Cu-free UiO-66­(COOH)_2_ sample (pristine of the (0:12)-Cu sample), the results reveal, as expected, no significant changes in the Zr oxidation state or coordination environment during both activation and reaction conditions. The Zr K-edge XANES and TF-XAFS spectra show similar spectral features after the activation and reaction conditions (Figure S16). However, the disappearance of a shoulder in the first peak around 2 Å and a decrease in the amplitude of the second peak at ∼3.2 Å in the TF-XAFS are observed after activation and reaction. This change may be attributed to the photothermal behavior of the sample, caused by the temperature increase upon lamp illumination. This aligns with the gas-phase analysis, which shows only water desorption during activation and no activity during the reaction.^62^ So, the activity mainly depends on the Cu centers and their coordination with the COOH groups and not on the concentration of free COOH groups present. After that, we proceeded to study the Zr–K edge of the sample (0:12)-Cu, post-metalated with Cu. During photoactivation under Ar (applying similar conditions used in the *operando* FTIR study), slight flattening of the white line of XANES spectra can be observed and accompanied by the removal of weakly coordinated water molecules (Figure S18) as indicated by the reaction gas-phase analysis (Figure S17). Furthermore, the effect of short-range-level coordination of Zr is found to be much more prominent (Figure S18 inset). Although the distortion of the first shell scattering (SS) Zr–O_SS_ was not noticeable, a significant change occurred in the Zr–Zr_SS_, where the pristine peak at 3.26 Å contracted to 3 Å.^63^ Under the reaction conditions, Zr-XANES did not reveal any significant structural change, and EXAFS shows intensity alteration of both Zr–O_SS_ and Zr–Zr_SS_ without any structural distortion. These findings suggest that this modification is more related to water desorption, while the Zr clusters are apparently nonimpacted by the anhydride formation observed by *operando* FTIR. It also confirms that only the free carboxylates (non-coordinated to Zr nodes) are involved in the anhydride formation and restructuring phenomena.

Regarding the Cu K-edge XANES, the spectrum of pristine (0:12)-Cu exhibits a weak quadrupole-allowed (1s → 3d) pre-edge feature at ∼8977.5 eV, followed by a peak at ∼8985.5 eV corresponding to the dipole-allowed (1s → 4p) transition, both characteristic of Cu­(II) (Figure 5A).^64–66^ The corresponding Fourier transform of the EXAFS signals reveals two peaks at ∼1.57 and ∼2.57 Å (not corrected from the phase shift), assigned to Cu–O and Cu–Cu coordination in Cu­(II), respectively (Figure S18).^64,66,67^ In addition, a comparison with reference Cu_2_O spectra suggests the presence of a minor fraction of Cu^(I)^, likely generated during the postsynthesis activation by drying in the vacuum oven (solvent removal) step, alongside the dominant Cu^(II)^ species in the Cu-metalated MOF (Figure S19). This was further confirmed by their corresponding first derivatives; the observed absorption edge position confirms that copper is present predominantly in the +II oxidation state (Figure S20). As shown in this figure, the XANES first-derivative spectrum displays a maximum at 8983.8 eV, confirming the oxidation state of Cu. For comparison, the first derivative maximum of CuO appears at 8985.3 eV. It should be noted that the absorption edge position is influenced not only by the oxidation state but also by the local coordination environment of the absorbing atom. For instance, Gaur and coworkers reported Cu K-edge positions ranging from 8983.6 to 8984.9 eV for a series of six Cu^(II)^ complexes, depending on their structural geometry.^66^ During both photoactivation and FAc adsorption in the dark, a slight shift of the Cu-XANES pre-edge to lower energy and a decrease in intensity were observed and could be due to the desorption of coordinated water molecules (Figure 5A). The split pre-edge peak observed after FAc adsorption can be rationalized by examining the first derivative of the Cu K-edge XANES spectra for (0:12)-Cu in its pristine state, after activation under λ ≥ 390 nm and following FAc adsorption on its surface (Figure S21). In the pristine material, the pre-edge feature at ∼8985.5 eV appears split, most likely due to water desorption, which perturbs the local symmetry of the Cu^(II)^ centers. This dehydration event strengthens the interaction between Cu^(II)^ ions and the framework's free −COO^–^ groups. Upon FAc adsorption, analogous Cu^(II)^–carboxylate interactions are further reinforced, thereby preserving the overall coordination geometry of the Cu centers even after FAc binding to the MOF surface. However, during the reaction, we observed the arousal of a new peak at 8981.72 eV, a flattening of the white line, accompanied by H_2_ production (Figure 5B inset), and a progressive decrease in the Cu^(II)^ characteristic peaks at 8977.7 and 8985.2 eV (Figure 5B). These findings confirm the restructuring of coordinated Cu^(II)^ and their reduction to Cu^(I)^/Cu^(0)^ species during the photocatalytic dehydrogenation of FAc. Furthermore, Cu-EXAFS signals of Cu^(II)^ present initially decrease as the Cu–Cu_SS_ located at 2.2 Å increases, confirming the clustering of metallic copper (Figure 5C). Also, the first shell Cu–O_SS_ is shifted toward a shorter distance from 1.57 to 1.43 Å, which is assigned for the first shell Cu–O_SS_ of oxo-Cu^(I)^, demonstrating the coexistence of metallic Cu clusters and Cu_2_O inside the MOF framework.

**Figure 5:**
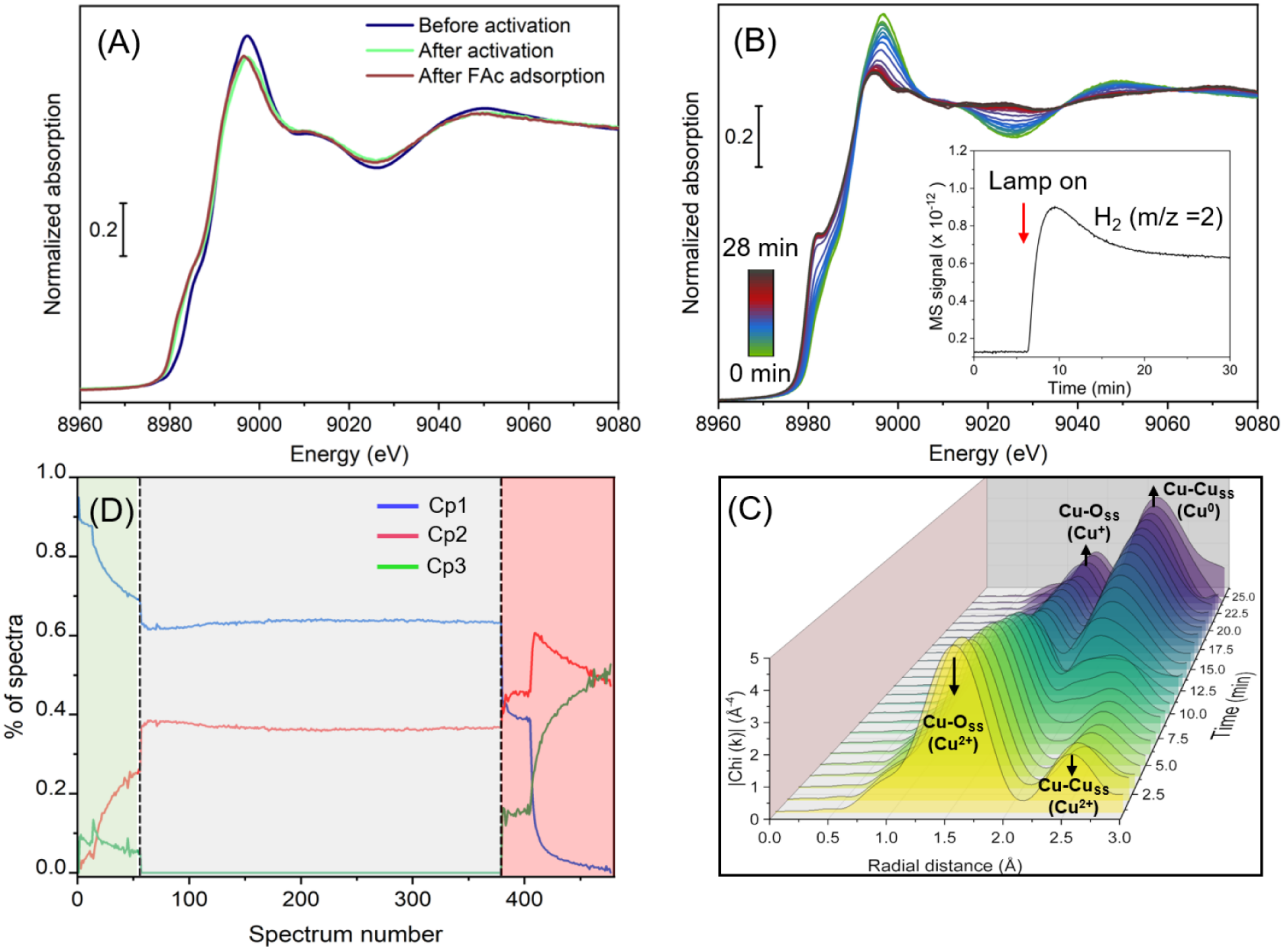
(A) The normalized Cu K-edge XANES spectra of (0:12)-Cu at different catalytic stages at steady state, (B) evolution of the normalized Cu K-edge XANES (inset: corresponding MS for H_2_ evolution) during the reaction catalyzed by (0:12)-Cu, (C) the Cu K-edge EXAFS spectra of the (0:12)-Cu sample during the photocatalytic reaction, and (D) concentration profiles of the (0:12)-Cu sample as a function of time, obtained from the MCR-ALS analysis. In (D), the green, gray, and pink parts correspond to the activation of MOF (≥390 nm, dry Ar), FAc adsorption (under dry Ar), and the photocatalytic reaction (≥390 nm, FAc), respectively.

To gain deeper insight into the structural evolution and oxidation state distribution of the Cu species during the photocatalytic process, time-resolved Cu K-edge quick-EXAFS datasets were further analyzed using principal component analysis (PCA) and multivariate curve resolution with alternating least-squares (MCR-ALS) for which a complete description of the analysis is given in the Supporting Information file (Figures S22-S24). We emphasize, however, that MCR-ALS should be regarded as a semiquantitative approach, since the extracted concentration profiles can be influenced by several factors, including the choice of reference, the number of components selected, and other input parameters. Nevertheless, these analyses could provide a coherent picture of the Cu restructuring process. The MCR-ALS minimization was performed considering three components. The obtained component spectra of (0:12)-Cu are given in Figure S25B, resulting from the quick-XAS data at the Cu K-edge for (0:12)-Cu (Figure S25A). The first component (Cp1) corresponds to the Cu species present in the spectrum of the pristine MOF, i.e., Cu in the +II oxidation state. The fit of the corresponding EXAFS signal (Figure S29 and Table S4) confirms the oxidation state with a Cu–O distance of 1.95Å as in the CuO reference (Figure S27 and Table S2). The EXAFS fitting of this component further revealed a distorted octahedral geometry (Table S4). This observation is in line with the Raman results discussed above. Component 2 (Cp2) is identified as a Cu^(I)^ species, as revealed by the position of the first derivative of the XANES (8980 eV) (see Figure S26) as well as the EXAFS fit results (Figure S30 and Table S5) which exhibit a Cu–O distance at 1.86 Å in line with the Cu_2_O species. The third component (Cp3) exhibits an edge position at 8979 eV (Figure S26), relevant to Cu^(0)^. The EXAFS fitting of Cp3 revealed a *fcc* geometry (Figure S31 and Table S6). Particularly, the coordination number was found to be lower (4.4 and 2.1 in the first and second coordination shell, respectively) than the typical metallic Cu (12 and 6 in the first and second coordination shell, respectively) as illustrated in Figure S28 and Table S3, suggesting nanosized metallic Cu cluster. The smaller size of metallic Cu could be responsible for higher photocatalytic activity of this sample. The concentration profile of the three components for (0:12)-Cu is given in Figure 5D. During the activation process (irradiation with ≥390 nm under dry Ar), Cp1 starts to decrease to the benefit of Cp2 (Cu­(I) ∼25%). This is likely due to residual FAc remaining within the cell, which promotes the partial reduction of Cu^(II)^ under irradiation. During the FAc adsorption process, the percentage of Cu^(I)^ remained constant throughout the process. Then, during the photocatalytic conversion of FAc into H_2_, a mixture of 50% Cu^(0)^ and 50% Cu^(I)^ is reached at steady state. These results are in line with what was observed in the *operando* FTIR study and highlight the important role of Cu coordination and the Cu oxidation state for both restructuring and photocatalytic activity. Subsequently, a comparison between the three samples, i.e., (0:12)-Cu, (6:6)-Cu, and (9:3)-Cu, was carried out to understand the origin of their distinct performances. Results showed similar Cu-XANES and Cu-EXAFS behaviors of the pristine materials prior to activation, suggesting a similar local and long-range coordination system of Cu (Figure S32). Conversely, the XAS spectra during the photocatalytic reaction revealed a more pronounced formation of metallic Cu from (0:12)-Cu to (9:3)-Cu, as shown by the flattening of the white line in XANES and intensifying the Cu–Cu_SS_ first shell in EXAFS spectra. This aligns with the HR-TEM analysis which shows a smaller particle size of Cu in (6:6)-Cu as compared to the (0:12)-Cu sample. However, as the data are relative (optimization of the condition to get a good signal), the lower activity of these samples can be as well related to the fewer coordinated Cu which restructured due to the decrease in BTCA concentration in the UiO-66­(COOH)_*x*_-Cu structures.

### Investigation of the Charge Carrier Dynamics

To better understand the charge dynamics of the process and to demonstrate the important role of the UiO-66-(COOH)_2_ framework in the photocatalytic activity, steady-state photoluminescence was carried out on the most active sample, i.e., (0:12)-Cu before and after the reaction, and the results are illustrated in Figure 6A,B. Upon excitation from 340 to 650 nm, a prominent emission band appears around 430 nm that is likely due to a ligand to Zr-oxo-cluster charge transfer.^68,69^ After the reaction, the PL intensity at 430 nm decreased compared to the prereaction state, indicating significant PL quenching. Furthermore, a new emission band, centered at 580 nm and attributed to the band edge emission of Cu_2_O, appeared indicating the formation of new Cu species after the restructuring process.^70^ The PL measurements carried out on Cu_2_O reference (Figure 6C) aligned with the observed emission band of the restructured (0:12)-Cu sample, confirming the transformation of coordinated Cu^(II)^ into reduced Cu clusters, in agreement with previous data.

**Figure 6:**
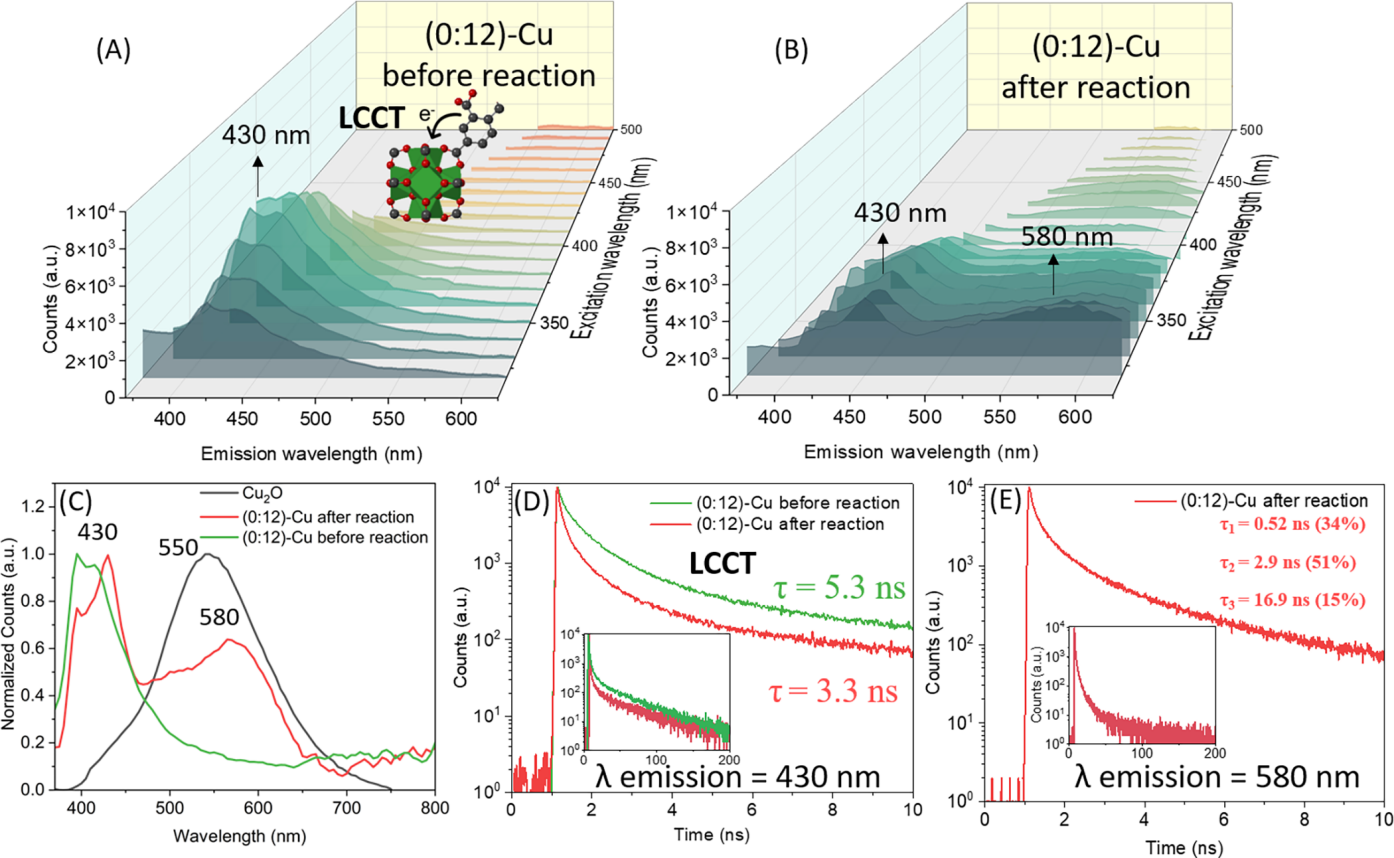
3D plots of the PL emission maps of (0:12)-Cu before (A) and after (B) reaction. (C) Normalized PL intensities of the corresponding samples after excitation at 340 nm. Cu_2_O is added as reference. (D) and (E) report the decay transient of the photoluminescence of the corresponding samples upon 343 nm excitation at emission wavelengths of 430 and 580 nm, corresponding to the LCCT and band edge emission of Cu_2_O transient, respectively. Inset: zoomed-in view of the 200 ns time scale.

Time-resolved photoluminescence (TR-PL) spectroscopy was then employed to investigate the dynamic behavior of the excited state and gain further insights into the charge transfer processes during the reaction. The decay at 430 nm was monitored using a 343 nm femtosecond pulsed laser, and its lifetime was found to decrease from 5.3 to 3.3 ns after the reaction (Figure 6D and Table S7). This reduction in lifetime could be attributed to charge transfer processes related to *in situ* restructuring and/or to the formation of surface anhydride species that could alter the electronic states of the MOF. In addition, the transient decay of the emission at 580 nm was also monitored as shown in Figure 6E. Different lifetime decays were observed, with a dominant component of a lifetime of 2.9 ns. Comparison between the Cu_2_O/Cu^(0)^ sample and pure Cu_2_O showed that both exhibited a similar emission band around 550 nm (Figure S33). TR-PL analysis further revealed that the lifetime decay decreased in the presence of Cu^(0)^, reaching 3.4 ns, highlighting the importance of Cu^(0)^ in charge carrier dynamics. Notably, for (0:12)-Cu, the lifetime was further reduced to 2.9 ns, which is four times shorter than that of pure Cu_2_O (Table S8). These results are probably due to charge transfer from Cu_2_O to Cu^(0)^ during the *in situ* restructuring process. Overall, the findings underscore the dual function of UiO-66­(COOH)_2_-Cu not only in stabilizing the Cu_2_O/Cu^(0)^ clusters during the reaction but also promoting the charge transfer from the Zr-oxo-clusters to the restructured Cu species and enhancing the *in situ* restructuring process.

Based on all these findings, a charge transfer mechanism, following the restructuring at steady state, can thus be proposed, as illustrated in Scheme 1. Under solar-simulated irradiation, an electron is excited from the HOCO of UiO-66­(COOH)_2_ to its LUCO, leaving behind a hole in HOCO (I). Following the initial excitation, the electron in the LUCO of UiO-66­(COOH)_2_ is transferred to the conduction band (CB) of Cu_2_O (II). This photogenerated hole on the HOCO can then be neutralized through two competing pathways: (1) by electron transfer from the valence band (VB) of Cu_2_O to the HOCO of UiO-66­(COOH)_2_, thus promoting better charge separation and enhancing charge carrier mobility (III-a); or (2) by directly oxidizing FAc to produce protons and formate (III-b). Although the second pathway is less favored in this system, its occurrence cannot be completely excluded as it may lead to the formation of monodentate formate species that act as intermediates in the dehydrogenation pathway. However, this direct hole-driven oxidation pathway is highly favored in the ZrO_2_:Cu_2_O system, leading predominantly to the formation of bidentate formates and subsequently producing CO and H_2_O. Then, the holes generated in the Cu_2_O VB react with FAc to produce protons and formates (IV). Finally, the electrons in the CB of Cu_2_O are transferred to metallic Cu sites (V), facilitating the evolution of H_2_ and CO_2_ (VI).

**Scheme 1:**
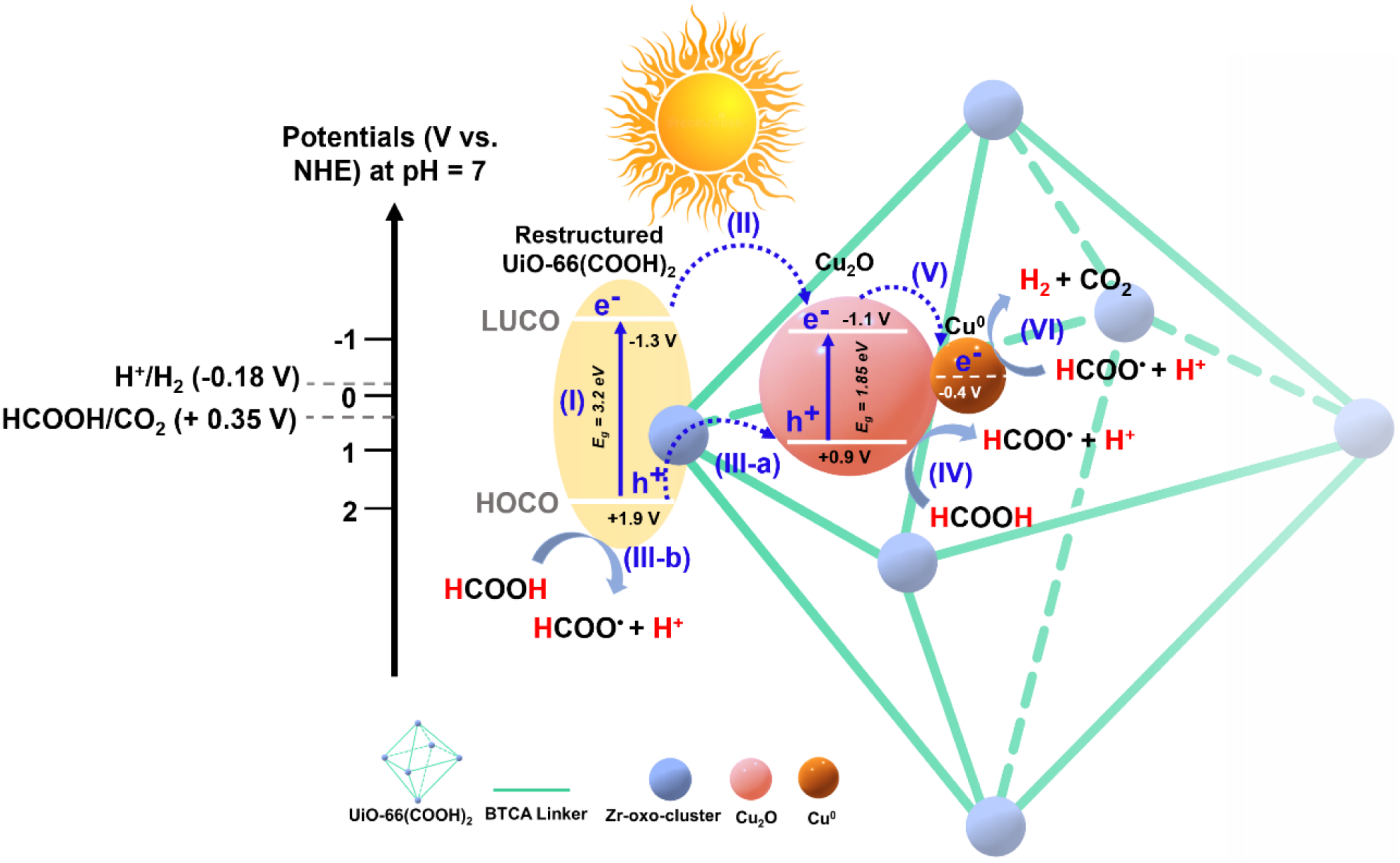
Proposed Mechanism of the Photoinduced Charge Transfer and the FAc Dehydrogenation over the Restructured UiO-66­(COOH)_2_-Cu at Steady State during Photocatalytic Dehydrogenation of FAc.

### DFT Calculations

DFT calculations were performed to gain deeper insights into how the spatial distribution and concentration of free carboxylate groups in the MOFs affect Cu coordination, as well as the different rates of anhydride formation among various samples. The (10:2) sample incorporating 10 BDC and 2 BTCA linkers per unit-cell (Table 1) was first modeled. The sample was chosen to investigate the effect of the free carboxylate spatial position inside the UiO-66 cage on the Cu–OOCH interaction strength. Three possible structural arrangements of the 2 BTCA linkers (shown in Figure 7A) were initially considered starting with the cubic unit-cell of UiO-66 (Zr) and were further subjected to DFT-geometry optimization. Their corresponding calculated relative energies reported in Figure 7B show that the incorporation of 2 BTCA linkers facing each other is not energetically favorable. Among the three configurations, the most stable MOF structure model exhibits an arrangement of the BTCA linkers that minimizes steric repulsion between adjacent carboxylate groups. This was further confirmed by analyzing the distance between the two free carboxylate groups in such structure models, with the most stable configuration being obtained when this distance is 7.9 Å (Figure S34). These results suggest that the optimal configuration is driven by space constraints. As a further step, we explored the incorporation of 1 Cu^(II)^ atom into the three possible pristine DFT-optimized (10:2) samples. To accurately simulate the Cu^(II)^-loaded MOFs, we considered the original −COOH functional groups to be deprotonated to form −COO^–^, allowing for proper Cu^(II)^ coordination. Figure 7C presents the corresponding Cu^(II)^-loaded configurations, showing that the BTCA/BDC configurations in the (10:2) sample significantly impact the structural and coordination environment of the Cu^(II)^. These DFT results evidence that the configuration in which Cu^(II)^ interacts with neighboring carboxylic groups is characterized by a lower total energy, indicating stronger coordination forces.

**Figure 7:**
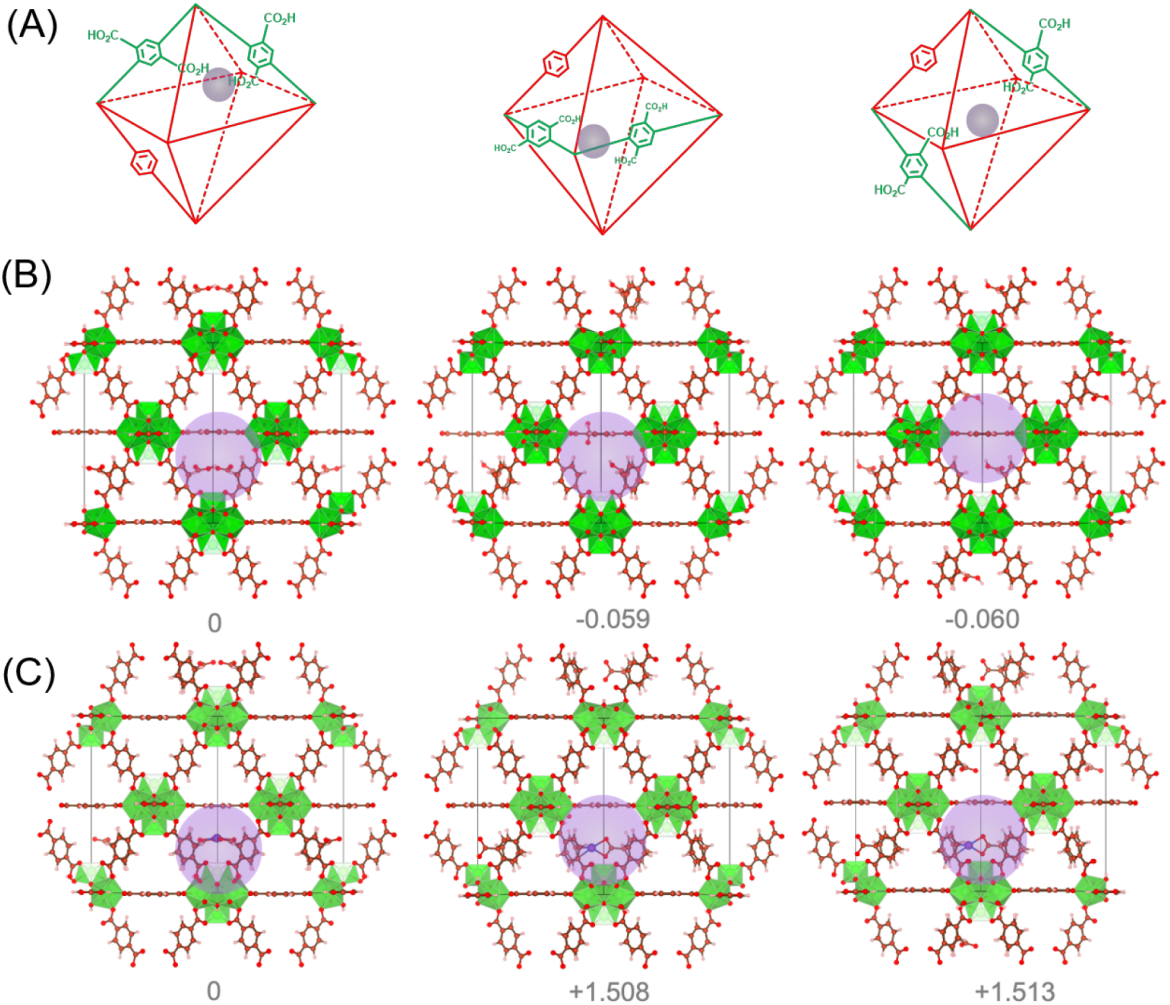
(A) Schematic representation of the possible configurations of the (10:2) sample (three typical BTC/BTCA combinations are considered) with the violet sphere highlighting the possible Cu coordination position, (B) the DFT-optimized configurations of the (10:2) sample, and (C) their DFT-optimized unit-cells loaded with one Cu­(II) (when Cu­(II) is incorporated (violet circle), the two neighboring free −COOH groups are equivalent to one −COO^–^ group). The relative energy values of the different configurations are also shown below with the energy unit eV/unit-cell. Color codes: C, brown; Cu, violet; O, red; H, white; Zr, green.

We further explored the (9:3) sample containing 9 BDC and 3 BTCA linkers per unit-cell. The DFT-optimized configurations, considering four possible structural arrangements of the BTCA linkers, are illustrated in Figure 8A. Figure 8B shows that the degree of stability of the pristine MOF framework depends on the BTCA linker distribution, but overall, the energy differences between all configurations are rather small (within 0.165 eV/unit-cell). When Cu^(II)^ is introduced (Figure 8C), the total energies of the four configurations are still rather similar (energy difference within 0.332 eV per MOF unit-cell), suggesting that the different spatial distributions of BTCA linkers in this scenario are not expected to drastically affect the relative stability of the overall (9:3)-Cu. Therefore, the experimentally prepared (9:3) sample likely exhibits a random spatial distribution of BTCA linkers, regardless of whether Cu^(II)^ is introduced. To further validate the influence of linker spatial distribution, we extended our periodic DFT survey to include additional representative linker arrangements at a BDC:BTCA = 8:4 composition. Several distinct spatial distributions of the BTCA linkers were generated and fully geometry-optimized using the same computational protocol described above. As shown in Figure S35, the results reveal a clear energetic preference landscape: one arrangement (configuration 3) is substantially higher in total energy relative to the other arrangements, while the remaining configurations lie within a very narrow energy window. Analysis of the optimized geometries indicates that the elevated energy of configuration 3 arises from severe steric crowding when multiple free −COOH groups are positioned adjacent to the same octahedral vertex (top-corner) of the UiO-66 cage. In that geometry, interlinker repulsion and local distortion of the Zr_6_ node are amplified, resulting in an unfavorable energetic penalty. These observations support the view that (i) the overall number of free carboxylates and (ii) their local spatial arrangement together determine the thermodynamic propensity for anhydride formation and the stabilization of restructured Cu motifs.

**Figure 8:**
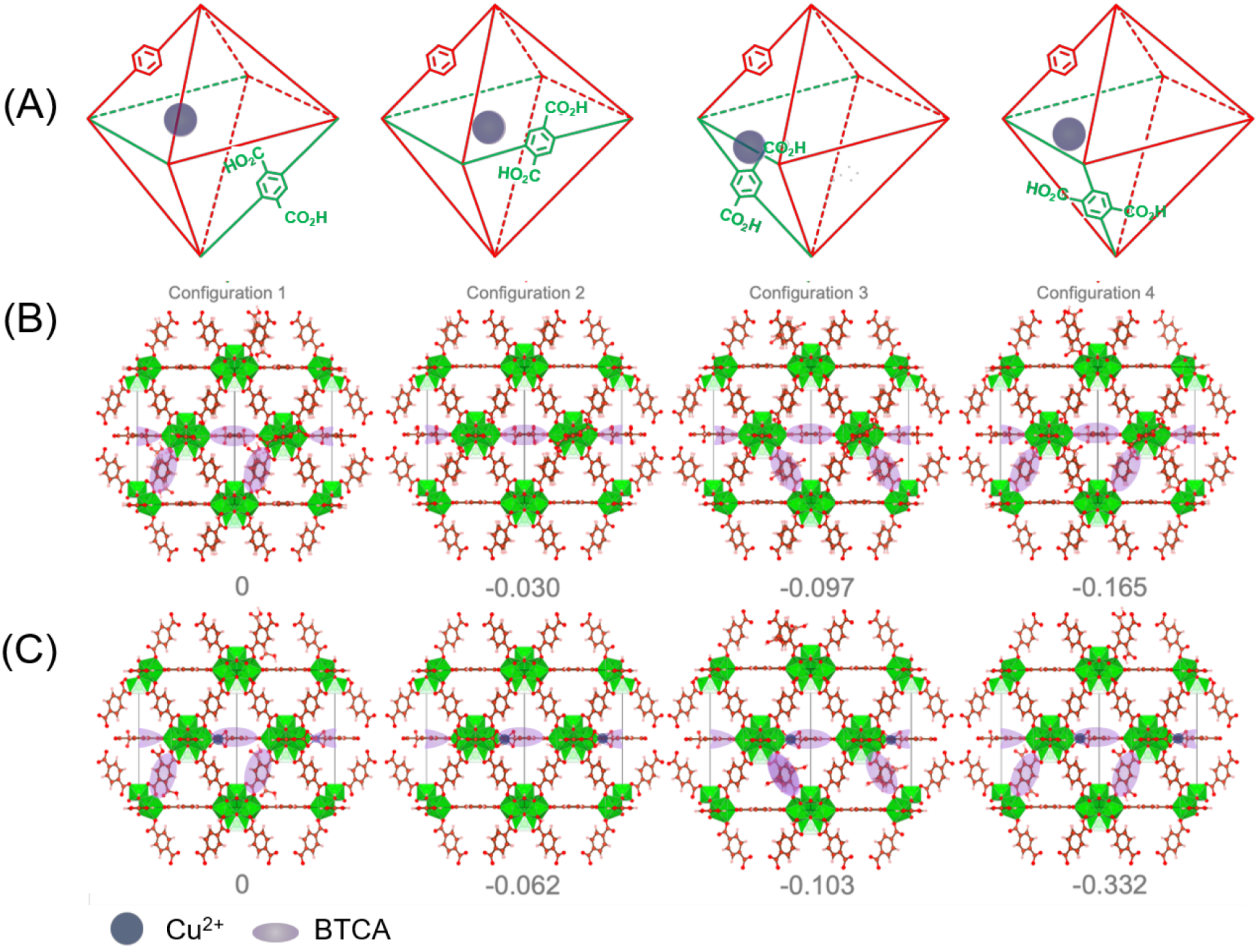
(A) Schematic illustration of the possible configurations of the (9:3) sample (four typical BDC/BTCA combinations considered) with the violet circle representing the possible Cu coordination site, (B) their corresponding DFT-optimized configurations, and (C) their DFT-optimized unit-cells loaded with one Cu­(II) (when Cu­(II) is incorporated (violet circle), the two neighboring free −COOH groups are equivalent to one −COO^–^ group). The relative energy value of the different configurations is also shown below with the energy unit eV/unit-cell. Color codes: C, brown; Cu, violet; O, red; H, white; Zr, green.

It has also been demonstrated previously that the photocatalytic activity is proportional to the amount of anhydride species formed during the reaction. A greater quantity of anhydride reflects a higher degree of Cu restructuring, which, in turn, is influenced by the coordination environment of the Cu species and correlates with enhanced photocatalytic performance. Moreover, the spatial orientation of the free −COOH groups and the overall framework geometry play crucial roles not only in stabilizing the restructured Cu species but also in modulating the rate at which restructuring occurs. This is particularly evident in the (9:3)-Cu sample, as illustrated in Figure 2C. Although this sample does not have the highest concentration of free −COOH groups, their favorable spatial distribution promotes a higher rate of anhydride formation, suggesting a more favorable kinetic and thermodynamic environment for Cu restructuring. In comparison, the (0:12)-Cu sample, which recorded the highest anhydride amount, ultimately exhibited the highest photocatalytic activity but had a lower kinetic rate of formation of anhydride compared to the (9:3)-Cu (Figure 2D, inset), highlighting the importance of both quantity and kinetics of restructuring in determining photocatalytic performance.

Furthermore, to gain atomistic insight into the structural evolution and cluster formation while respecting the methodological limitations, we did not attempt a full treatment of the Cu^(II)^ → Cu^(I)^ → Cu^(0)^ redox energetics since periodic plane-wave DFT requires compensating background charges that introduce artificial long-range effects. Instead, we carried out DFT calculations of Cu species/aggregates embedded within the MOF using neutral supercell models and a series of cluster-size variants. Specifically, the adsorption/embedding energies for Cu entities of different atom counts, under different loading scenarios, and with the experimentally relevant −COOH ligand modification present on the framework were computed. These calculations probe the relative thermodynamic preference for an isolated single Cu atom versus small clusters inside the MOF pores and how ligand modification stabilizes particular motifs. Our results showed that Cu1 and Cu3 clusters are thermodynamically most favorable (Figure S36). This stability is due to the fact that configurations containing one or three Cu atoms are energetically favored. In the case of the Cu3 motif, each Cu atom coordinates to the oxygen of a −COOH group, forming a triangular arrangement stabilized through three Cu–O bonds.

We then explored the dehydrogenation reaction HCOOH → CO_2_ + H_2_ using DFT reaction-pathway calculations and free-energy analysis on representative structural models (see Figure 9). Minimum-energy pathways were mapped, and key intermediates/transition states were located for the canonical dehydrogenation route. As shown in Figure 9, the first step proceeds via coordination of an HCOOH molecule to a Cu site, with an adsorption energy of approximately −0.7 eV, indicating that Cu sites readily accumulate HCOOH and thereby promote subsequent dehydrogenation. The adsorbed HCOOH (HCOOH*) undergoes O–H bond cleavage to form formate (HCOO*) and a surface hydrogen (H*); these intermediates subsequently convert to CO_2_ and H_2_. The Gibbs free-energy changes, computed for this elementary dehydrogenation step, are in line with prior theoretical reports and fall within the expected ranges for FAc dehydrogenation.^28,71–73^ We computed full Gibbs free-energy profiles for the dehydrogenation and competing dehydration routes on a representative UiO-66­(COOH)_2_-Cu structural model. The detailed free-energy landscapes and intermediate configurations are provided in Figures S37 and S38. These calculations confirm that upon adsorption, the dehydrogenation route proceeds via O–H cleavage to form HCOO* + H*, followed by hydrogen recombination and CO_2_ desorption. These energetic preferences provide a thermodynamic and kinetic rationale for the high selectivity of UiO-66­(COOH)_2_-Cu toward dehydrogenation (H_2_/CO_2_) observed experimentally in BTCA-rich samples.

**Figure 9:**
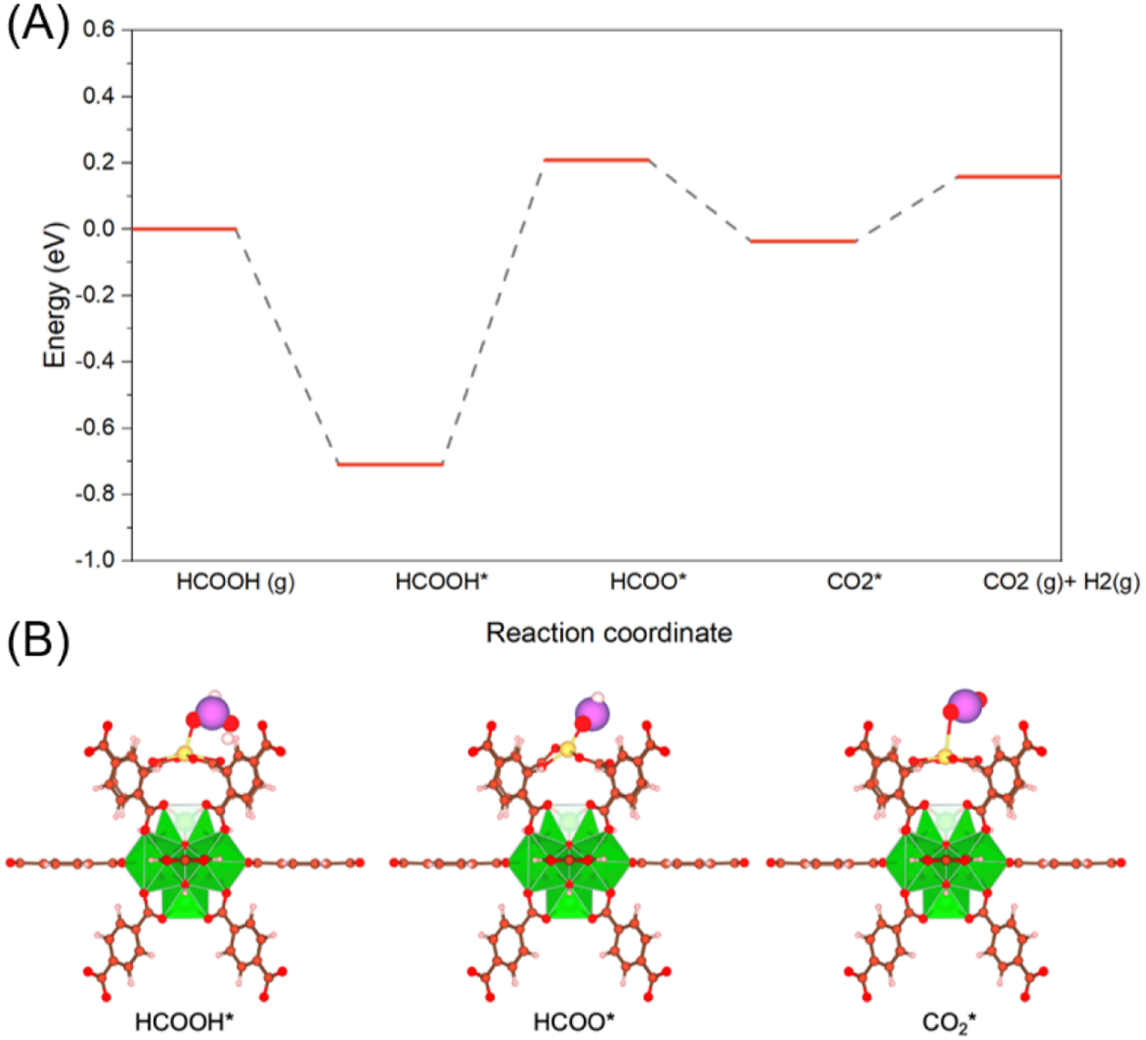
(A) The minimum-energy pathway derived from DFT for the dehydrogenation of FAc by UiO-66­(COOH)_2_-Cu is shown along with the corresponding (B) illustrative snapshots of the different intermediate species. The total free energy of the UiO-66­(COOH)_2_-Cu structure with a gas-phase HCOOH molecule was set to zero in the Gibbs free energy profile.

Based on all of these findings, the mechanism of the photocatalytic dehydrogenation of FAc is illustrated in Scheme 2. In the presence of FAc and under light irradiation, coordinated Cu^(II)^ species undergo a reduction to Cu^(I)^/Cu^(0)^ clusters simultaneously with the formation of anhydride groups, balancing the charge neutrality of the final structure. It should be noted that the anhydride formation in UiO-66-(COOH)_2_ requires a temperature higher than 110 °C,^47^ while in our reaction conditions, it occurred photochemically at around 25 °C. Therefore, a catalytic role of Cu and/or photothermal phenomena (local heating) is probably at the origin of the anhydride formation. This work underscores the importance of the unique structure of UiO-66­(COOH)_2_, where the anhydride bridges stabilize the restructured Cu species *in situ* under the standardized reaction conditions.

**Scheme 2:**
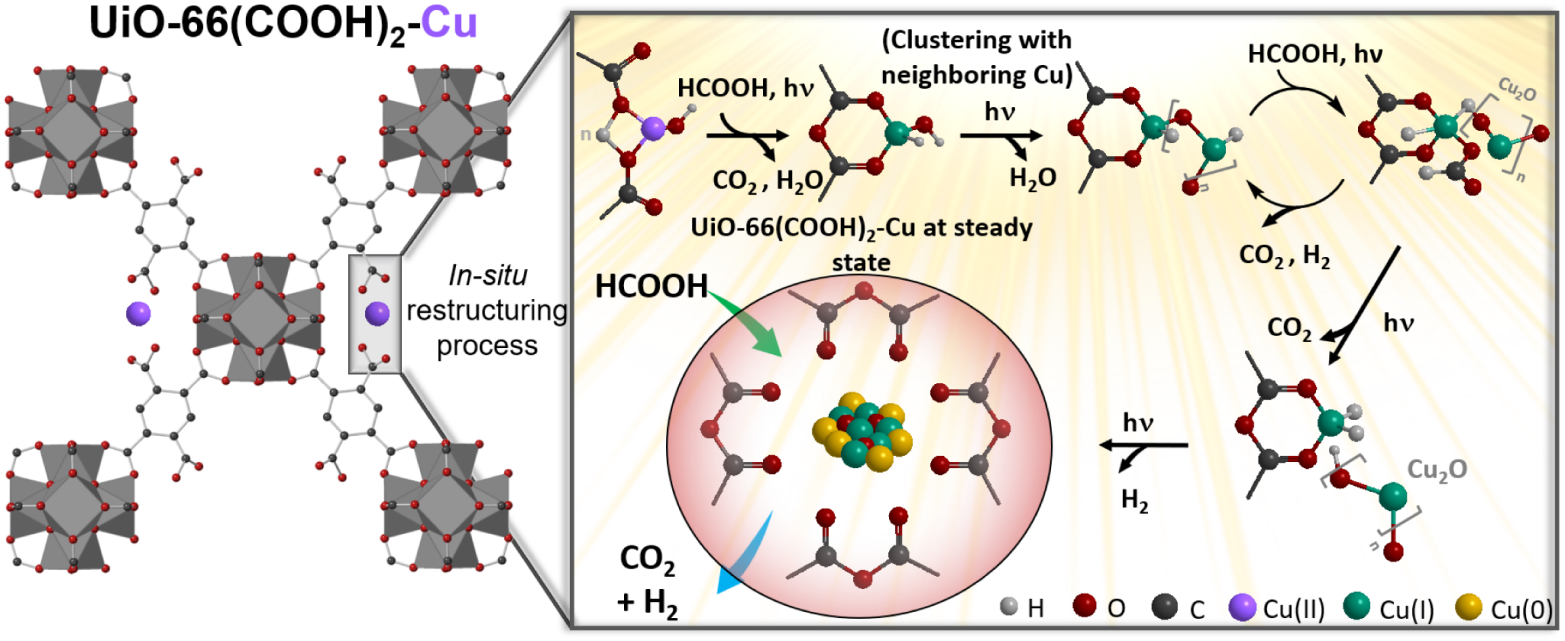
Illustration of the *In Situ* Restructuring Pathways of Cu Sites during Photocatalytic FAc Dehydrogenation over UiO-66­(COOH)_*x*_-Cu, Forming a Stabilized Photoactive Cu(0)/Cu­(I) Binary System

## Conclusion

The unique microporous cage-like structure of the robust UiO-66­(Zr) framework with its high capacity to be structurally tailored is regarded as a highly promising candidate for photocatalytic processes. In this study, a series of UiO-66­(COOH)_*x*_-Cu derivatives were synthesized via the MTV approach, yielding structures with varied BDC:BTCA ratios. This series not only improves the accessibility of the active sites but also offers a systematic way to demonstrate the effect of free carboxylate groups on the restructuring of Cu. Indeed, the photocatalytic performance proved to be proportional to the number of free functionalities, with the fully functionalized sample composed solely of BTCA recording the highest activity. This *in situ* restructuring process is now fully elucidated. *Operando* FTIR analysis showed the progressive formation of anhydride over the surface of the various structures, and a linear relation between the anhydride and the photocatalytic activity was established. *Operando* XAS clearly showed the reduction of Cu^(II)^ to Cu^(I)^ which is in turn reduced to Cu^0^ under solar-simulated irradiation in the presence of FAc, creating binary Cu_2_O/Cu^0^ active sites, being at the origin of the high photocatalytic activity. The charge carrier dynamics were also investigated using PL and TR-PL techniques, where the UiO-66­(COOH)_2_-Cu was proved to enhance the *in situ* restructuring process by promoting the charge transfer to the restructured Cu species. DFT calculations revealed that the (10:2) sample with (BDC:BTCA = 10:2) is expected to exhibit an optimal spatial distribution of the BTCA linkers facing each other in such a way as to favor the formation of a distorted octahedral Cu environment upon Cu^(II)^ incorporation. Conversely, in the case of the (9:3) sample with (BDC:BTCA = 9:3), the distribution of BTCA is not expected to substantially impact the structural stability of the MOF even upon Cu^(II)^ incorporation. These findings suggest that UiO-66­(COOH)_2_-Cu not only enhances the *in situ* restructuring process and promotes the charge transfer but also preserves the restructured Cu species through unique anhydride bridges, preventing their sintering. This feature overcomes aging, one of the main drawbacks of Cu_2_O-based materials, since it enables the *in situ* formation of an active Cu_2_O/Cu^0^ binary system that remains highly stable under the applied reaction conditions. In summary, this study highlights how the use of MOFs' structural flexibility can drive the photocatalytic dehydrogenation of FAc as a prominent source of H_2_ generation. Most importantly, it examines the *in situ* structural changes occurring within the Cu-metalated MOF structures, enabling the understanding of their mechanism of action, thus guiding the future design of potent catalysts for clean energy processes.

## Supplementary Material



## Notes

Schematic diagram of the *operando* photocatalytic reactor; ^1^H NMR analysis of linker incorporation in UiO-66­(COOH)_*x*_ derivatives; defect quantification from TGA with detailed calculations; pore-size distribution profiles; UV–vis spectrum of (0:12)–Cu; PXRD patterns and N_2_-sorption isotherms of Cu-metalated UiO-66­(COOH)_*x*_ derivatives; photocatalytic H_2_-evolution profiles during FAc dehydrogenation; operando FTIR surface analysis of UiO-66­(COOH)_*x*_-Cu; structural model of (12:0)-Cu showing Cu coordination; BTCA/BDC incorporation ratios and anhydride-band evolution; Raman analysis of (0:12)-Cu; methodology and calculation details for determining H_2_-production rate, apparent quantum efficiency, and TOF; post-reaction ATR-IR and HAADF-STEM/EDX analyses; operando Cu and Zr K-edge XANES and EXAFS datasets of the (0:12)-Cu along comparison with reference materials; multivariate MCR-ALS analysis of quick-XAS datasets; EXAFS fitting procedures and structural parameter outputs; time-resolved photophysical parameters; photoluminescence and decay analysis of Cu_2_O and Cu_2_O/Cu^(0)^; DFT calculations including minimum-energy pathways for FAc dehydrogenation and dehydration (PDF)
