# Supplementary material for: Mechanistic Insights into the In Situ Restructuring of Coordinated Copper in Postmetalated MOFs for Photocatalysis
Source: J Am Chem Soc. 2025 Dec 15;147(52):48331–51. doi: 10.1021/jacs.5c18407 (PMC12766680; doi:10.1021/jacs.5c18407)
Supplement: Supplementary file 1 [file ja5c18407_si_001.pdf]

Supporting Information

Mechanistic Insights into the *In-Situ* Restructuring of Coordinated Copper in Post-Metalated MOFs for Photocatalysis

Zahraa Abou Khalil [‡a], Karen Hannouche [‡b], Akashdeep Nath [a], Nisrine Assaad [c], Leen Farhat [b], Georges Mouchaham [c], Dong Fan [d], Oleg Lebedev [e], Anthony Beauvois [f], Ali Youssef [a], Valerie Briois [f], Guillaume Clet [a], Guillaume Maurin [d], Christian Serre [c], Marco Daturi [a], Mohamad Hmadeh [b]*, Mohamad El-Roz[a]*

[a] Université de Caen Normandie, ENSICAEN, UNICAEN, CNRS, Laboratoire Catalyse et Spectrochimie, 14000 Caen, France

[b] Department of Chemistry, American University of Beirut, Beirut; Lebanon

[c] Institut des Matériaux Poreux de Paris, Ecole Normale Supérieure, ESPCI Paris, CNRS, PSL University, Paris 75005, France

[d] ICGM, Univ. Montpellier, CNRS, ENSCM, Montpellier, France

[e] Université de Caen Normandie, ENSICAEN, UNICAEN, CNRS, Laboratoire CRISMAT, 14050 Caen, France

[f] Synchrotron SOLEIL, L'Orme des Merisiers, Départementale 128, 91190 Saint-Aubin, France

[‡] Equal contribution
*Email: mohamad.elroz@ensicaen.fr
*Email: mh210@aub.edu.lb

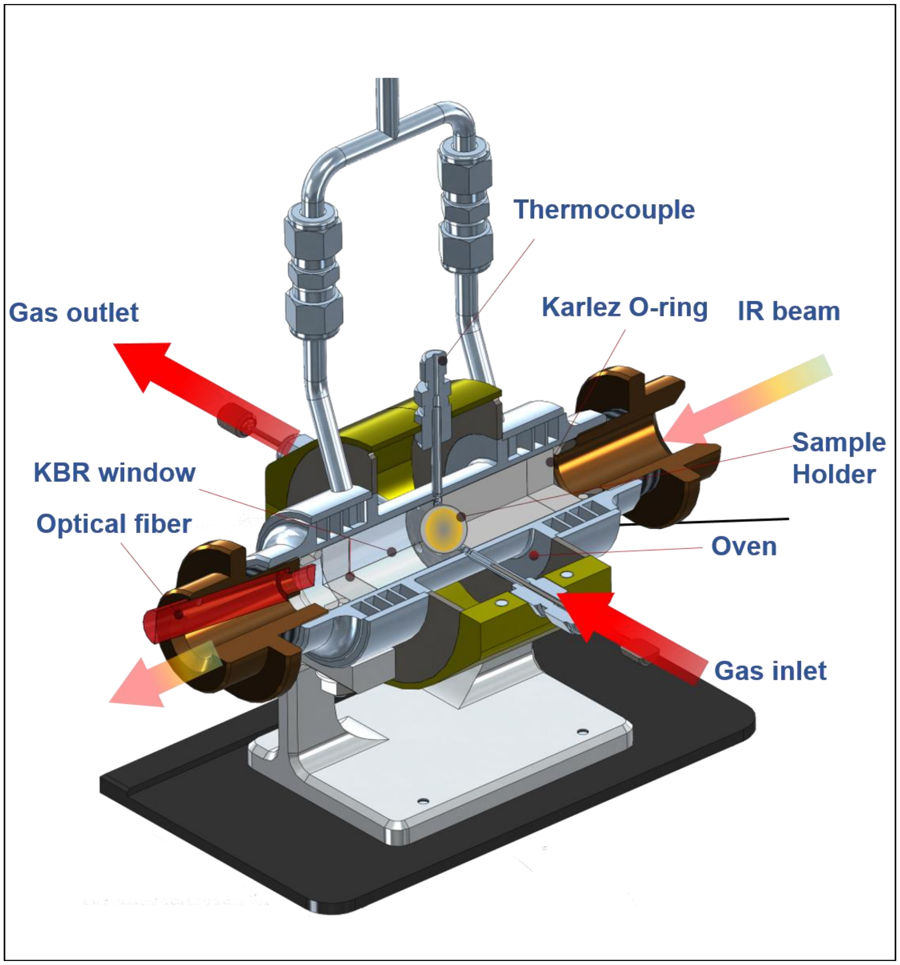

Scheme S1. Schematic diagram of IR sandwich cell reactor for the *operando* FTIR photocatalytic dehydrogenation of FAc[1].

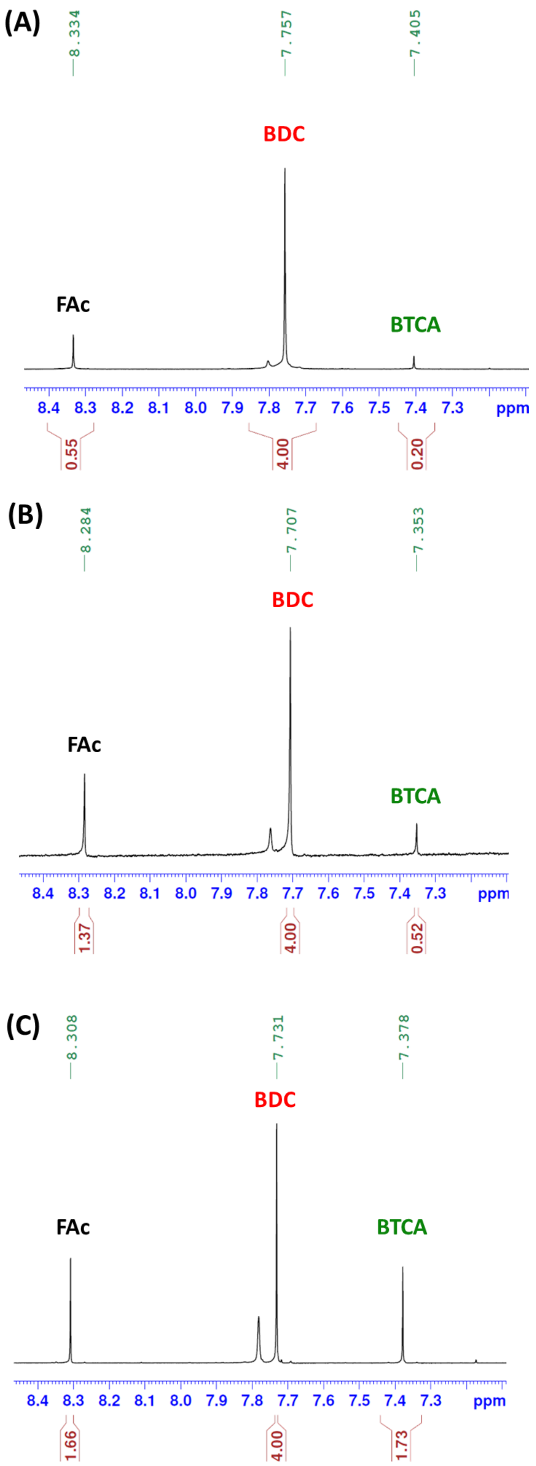

Figure S1. ^{1}H-NMR spectra of (A) (10:2), (B) (9:3) and (C) (6:6) MOFs to calculate the actual incorporation of benzenedicarboxylic acid (BDC) and 1,2,4,5-benzenetetracarboxylic acid (BTCA).

Defect number calculation

The defect number in each structure was estimated by normalizing each TGA curve such as the final weight (**WL_{final}**) is set to be 100%, and identifying major weight losses from the corresponding DSC curves. Each MOF, having a general formula of **$Zr_6O_4(OH)_4(linker)_6$** is thought to thermally decompose under air to give **6 ZrO_2** molecules. Considering the actual ratio of BDC:BTCA calculated from ^{1}H-NMR when calculating each MOF's molecular weight (**MW_{MOF}**), the theoretical weight loss **WLP_{th}** can be calculated as follows:

$$WLP_{th} = \frac{MW_{MOF}}{MW_{6ZrO_2}} \times WL_{final}$$

Considering a perfective UiO-66 structure, the ideal number of linkers (**NL_{th}**) would be equal to 6, and the weight loss per linker (denoted as **WL_{link}**) can be calculated by:

$$WL_{link} = \frac{WLP_{th} - WL_{final}}{NL_{th}}$$

In the actual defective UiO-66(COOH)$_x$ structures, **NL_{exp}** < **NL_{th}**, and **NL_{exp}** can be estimated from the temperature beyond which no more solvent or modulator loss occur (**T_{link}**), in other terms before the main exothermic DSC peak attributed to the organic linkers' oxidation. Ideally, the MOF structure should have a formula of $Zr_6O_6(linker)_6$ at this stage, and **WLP_{exp}** is the normalized weight corresponding to that temperature. **NL_{exp}** can then be calculated giving the number of missing linker defects (**NL_{mis}**) as follows:

$$NL_{exp} = \frac{WLP_{exp} - WL_{final}}{WL_{link}}$$

$$NL_{mis} = NL_{th} - NL_{exp}$$

The resulting defect numbers are tabulated in Table 1.

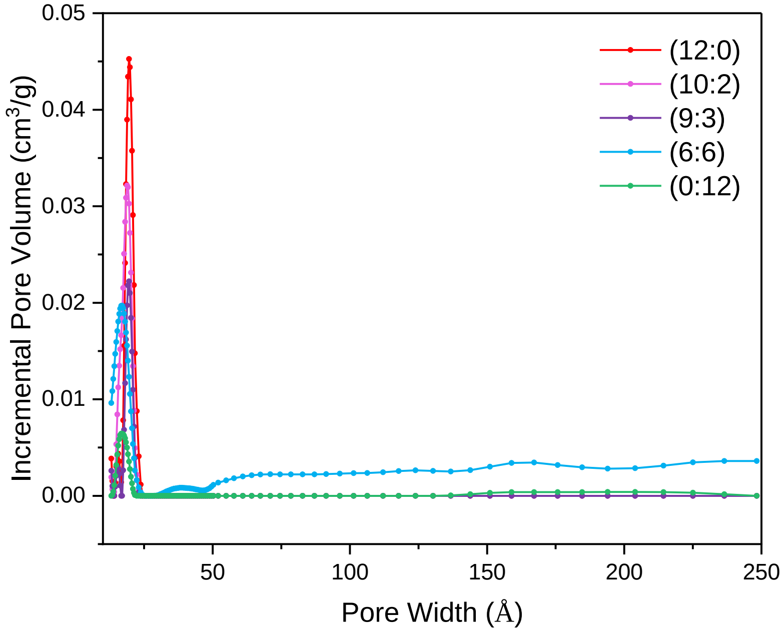

Figure S2. The pore size distribution profiles of the as-synthesized UiO-66(COOH)$_x$ samples.

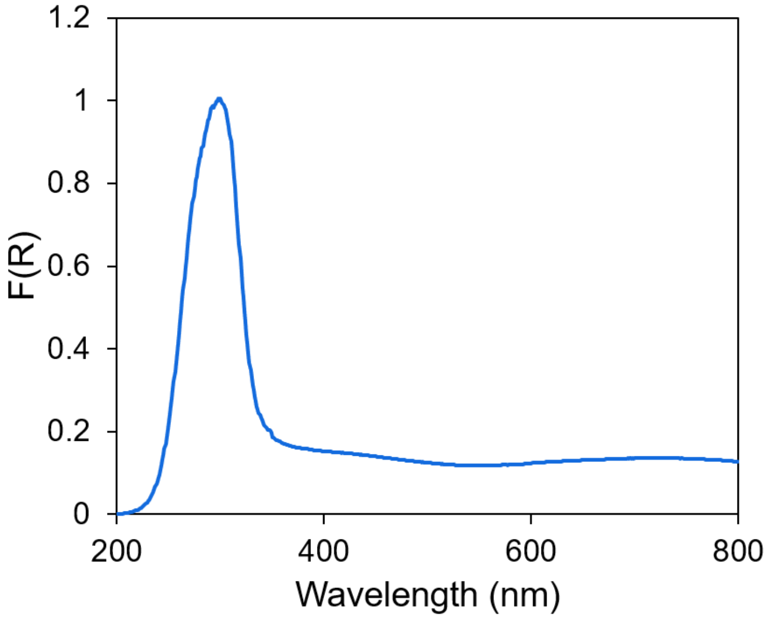

Figure S3. The UV-vis spectrum of the (0:12) sample after metalation with Cu showing an absorption band between 200 and 400 nm attributed to the LMCT of Cu^{2+}.

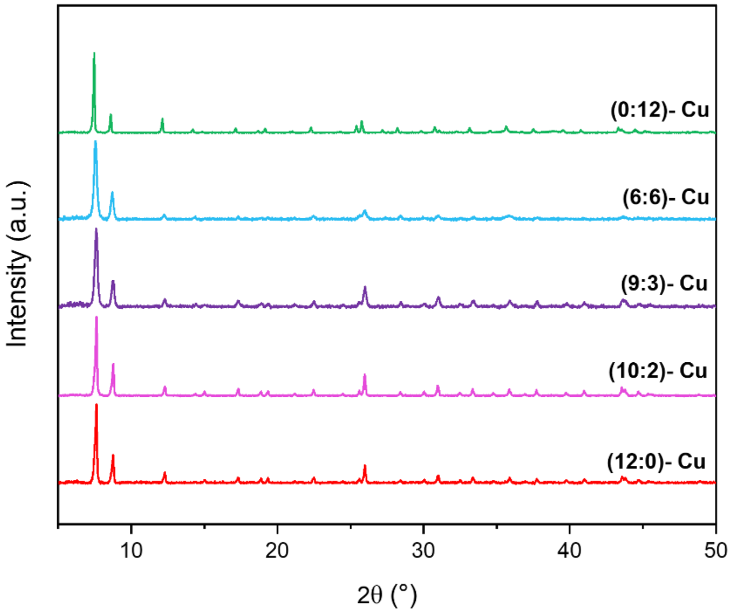

Figure S4. PXRD patterns of all UiO-66$(COOH)_x$ derivatives after Cu (II) metalation.

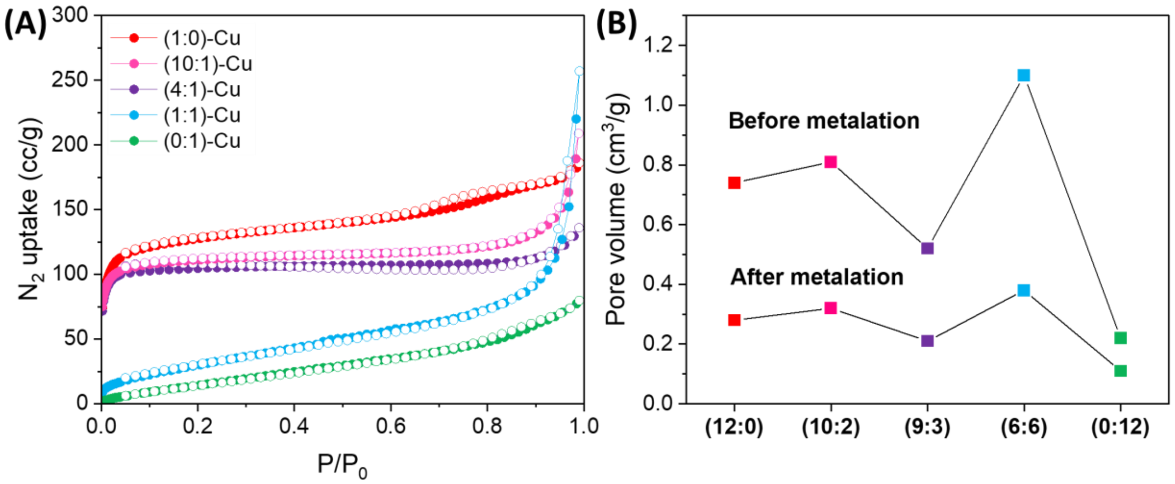

Figure S5. (A) N_2 sorption isotherms of all UiO-66$(COOH)_x$ derivatives after Cu (II) metalation. (B) Comparison between pore volumes of as-synthesized and Cu-metalated MOFs.

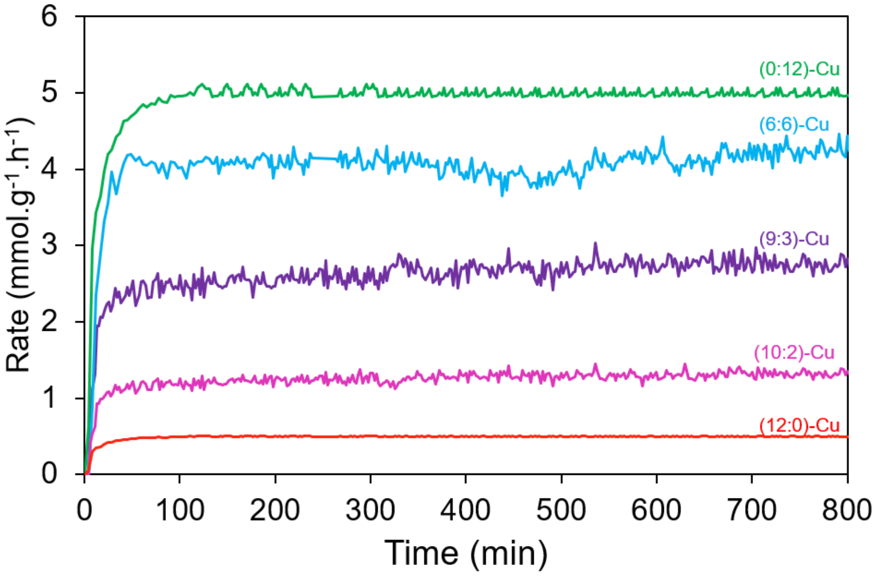

Figure S6. The evolution of the H_2 production rate of the various UiO-66(COOH)$_x$ samples during photocatalytic FAc dehydrogenation.

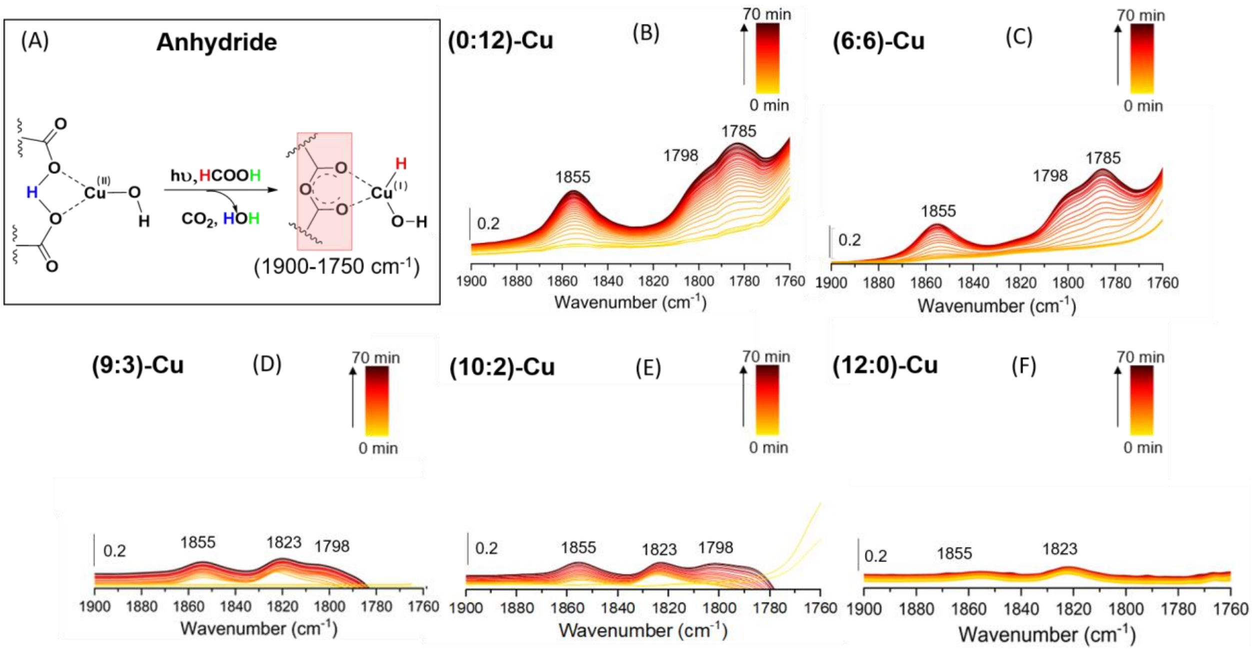

Figure S7. *Operando* FTIR surface analysis of the different UiO-66(COOH)$_x$ samples. (A) The possible reaction of anhydride formation and (B-F) evolution of the FTIR surface spectra in the

1900 – 1700 cm^{-1} corresponding to the anhydride region of the UiO-66(COOH)$_x$-Cu samples during the FAc dehydrogenation.

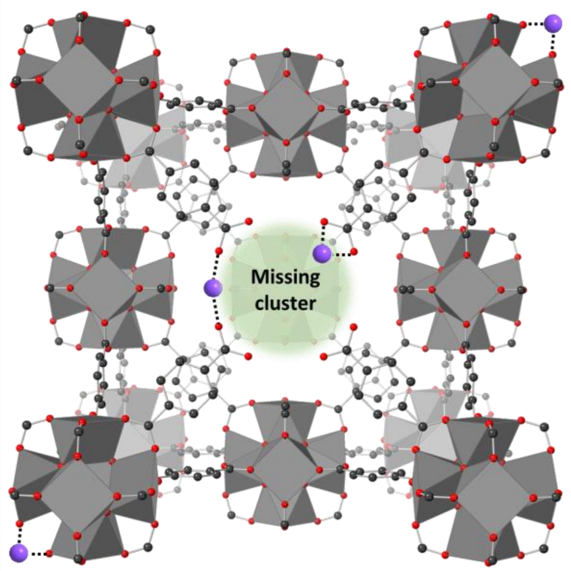

Figure S8. Crystal structure of (12:0)-Cu sample showing possible Cu (II) coordination within the non-functionalized (12:0) sample having missing linkers and missing clusters defects. *Zr in light grey, C in dark grey, O in red and Cu(II) in purple.*

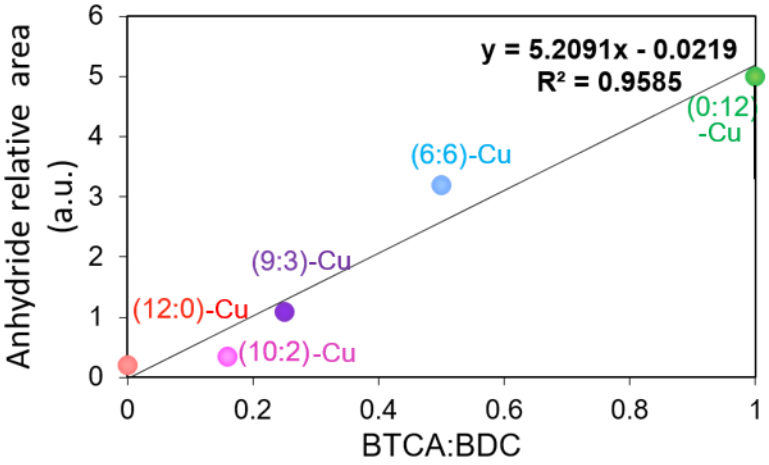

Figure S9. The variation of the anhydride band area versus the ratio of BDC:BTCA in the various UiO-66(COOH)$_x$-Cu samples.

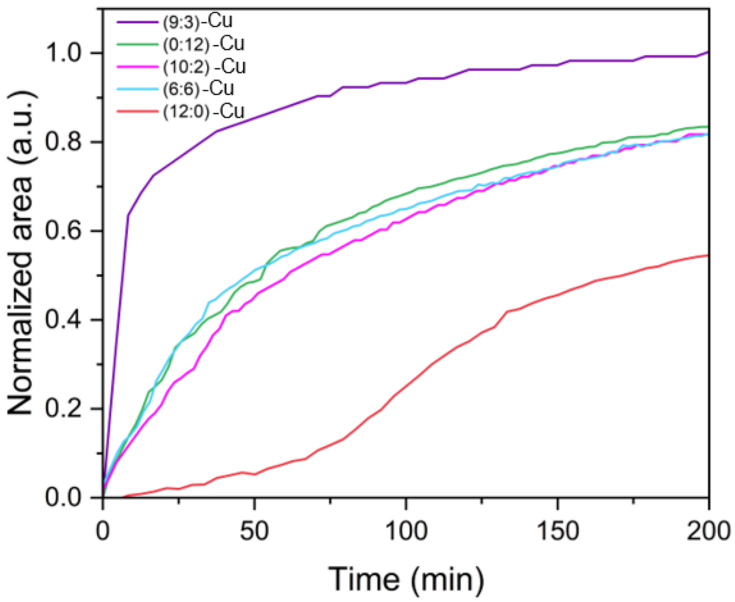

Figure S10. The evolution of the normalized anhydride band area in the various UiO-66(COOH)x-Cu samples versus time during the first 200 minutes of FAc photocatalytic dehydrogenation reaction.

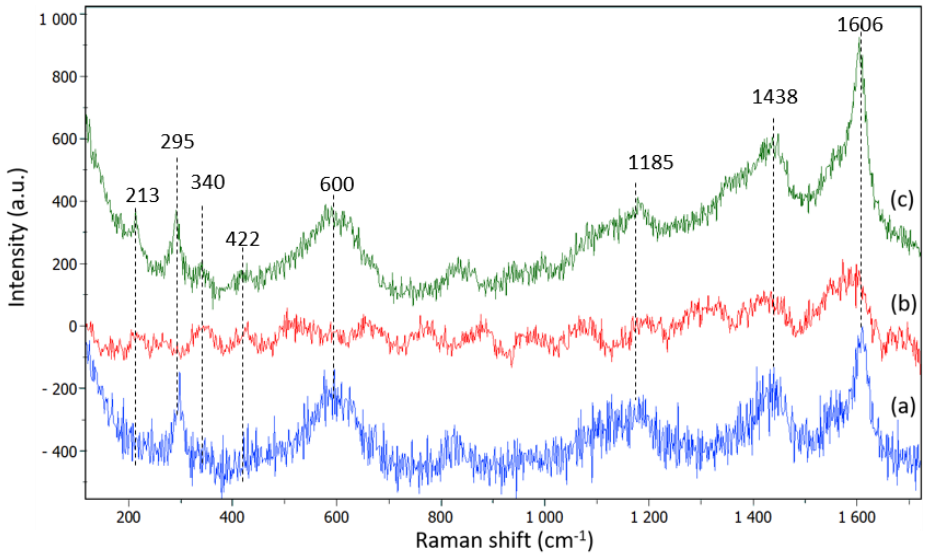

Figure S11. Raman analysis of (0:12)-Cu sample. Raman spectrum of (0:12)-Cu sample in (a) its state, (b) during reaction, (c) after reaction and removal from the *operando* cell.

Calculation of Hydrogen production rate, apparent quantum yield of H_2 production and the TOF values:

Under conditions of complete (100%) selectivity, the rate of hydrogen evolution is equivalent to the rate of formic acid (FAc) conversion, as determined by the equations presented below. The extent of formic acid conversion, expressed either as a percentage or normalized to mmol/g of photocatalyst per irradiated surface area, and the selectivity expressed as a percentage, were quantified at steady state using equations (5–7), based on calibration curves established for the detected reaction products. The apparent quantum yield was evaluated employing the actinometric method. The irradiated surface area of the photocatalyst pellet was approximately 1.6 cm². The formic acid conversion and the corresponding conversion rate were calculated as follows:

$$\text{FA conversion (\%)} = \frac{[\text{FA}]_0\ (\text{ppm}) - [\text{FA}]_t\ (\text{ppm})}{[\text{FA}]_0\ (\text{ppm})} \text{ x } 100 \qquad (1)$$

Where, $[FA]_0$ is the initial FAc concentration sent to the cell and $[FA]_t$ is the FAc concentration at a certain time (t) during the reaction.

$$\text{FAc conversion rate } (\text{mmol.g}^{-1}.\ \text{cm}^{-2}) =$$

$$\frac{\text{Total flow}\left(\text{L.min}^{-1}\right) \text{ x } [\text{FA}]0\ \ (\text{ppm}) \text{ x } 10^{-6} \text{x } \frac{\text{conversion \%}}{100} \text{x t}\left(\text{min}\right) \text{x } 1000}{\text{M}_{\text{FAc}}(\text{g.mol}^{-1}) \text{ x} \frac{1}{\text{d}_{\text{FAc}}}(\text{L.g}^{-1}) \text{ x m}_{\text{catalyst}}(\text{g}) \text{ x S}_{\text{irradiated}}(\text{cm}^2)} \qquad (2)$$

$$\text{Selectivity (\%)} = \frac{\text{Concentration of target product (ppm)}}{\text{Concentration of FA converted (ppm)}} \text{ x } 100 \qquad (3)$$

The apparent quantum yield for H_2 production was determined using the equation (4):

$$\mathbf{\Phi H2} = \frac{\mathbf{2\times molecules\ of\ hydrogen\ evolved\ (molecules/s)}}{\mathbf{number\ of\ incident\ photons}} \text{ x } f \qquad (4)$$

$$\text{With } f = \frac{\text{S(irradiated pellet)}}{\text{S(reactor used for actinometry)}} = \frac{1.6\ cm2}{18.1\ cm2} = 0.0884$$

The produced H_2 amount was quantified by FTIR and the incident photons were measured using $K_3Fe(C_2O_4)_3$ as a chemical actinometer. Thus, 15mL (V_1) of an aqueous solution containing the

iron actinometer (0.15 M) and H_2SO_4 (0.05 M) was prepared and added to the photocatalytic reactor. Before the irradiation, an aliquot of 0.180 mL (V_2) was taken and 2 mL of a buffered solution of phenanthroline (0.015 M / 0.5 M H_2SO_4) was added together with distilled H_2O to give a final volume of 25 mL (V_3) which maintained in dark 90 minutes to ensure total formation of complexes of Fe-Phenanthroline on dark conditions (baseline). Following, different irradiations times, between 30 and 510 s, with the 150 W Xe lamp used in the photocatalytic tests was made. The absorbance of the Fe-phenanthroline solutions were recorded (**FigureS12**) and the absorbance value at λ_{max} = 500 nm was used to calculate the number of Fe^{2+} ions (nFe^{2+}) generated during the irradiation process through equation (5):

$$nFe^{2+} = \frac{V_1\ x\ V_3(A - A_0)}{V_2\ x\ \varepsilon_0\ x\ 1000} \qquad (5)$$

V_1 = volume of actinometer solution irradiated (mL); V_2 = volume of aliquot taken for analysis (mL); V_3 = final volume to which the aliquot V_2 is diluted (mL); A = measured optical density at 510 nm; A_0 = measured optical density at 510 nm of a non-irradiated sample; ε_0 = experimental value of the molar extinction coefficient of the Fe^{2+} complex (11100 L mol^{-1} cm^{-1}).

$$nFe^{2+} = V_1 \times V_3(A-A_0)/\ V_2 \times \varepsilon_0 \times 1000 \qquad (6)$$

The number of incident photons per second (#photons) is calculated using the equation (7):

$$\text{Incident Photons } (s^{-1}) = \frac{6.022.10^{23}\ x\ nFe^{2+}}{\Phi_t\ x\ t} \quad (7)$$

Φt = quantum yield of Fe^{2+} formation (average 1.1) and t = time of irradiation (s).

The calculated incident photons per second per cm^2 in the studied time of $K_3Fe(C_2O_4)_3$ solution irradiation were found to be $3.178 x 10^{16}$ s^{-1}.

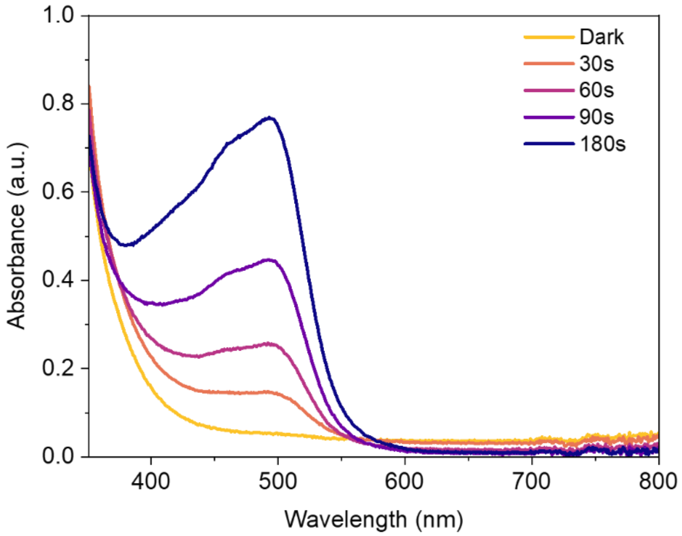

Figure S12. The absorbance of the Fe-phenanthroline solutions at different irradiation times.

The TOF values are calculated according to equation:

$$\text{TOF} = \frac{H2\ evolution\ rate\ \ (mol.s-1)}{moles\ of\ \ Cu\ active\ sites} \qquad (8)$$

Where number of moles of Cu is calculated from the Cu weight percentage.

Table S1: Results of FAc dehydrogenation of varied UiO-66(COOH)$_x$-Cu at steady state.

**Sample**	**H_2 evolution rate [a]**	**Dehydrogenation Rate [b]**	**Dehydrogenation rate [c]**	**Dehydrogenation rate [d]**	**% Cu**	**m (mg) of Cu**	**Cu (mol)**	**TOF(s^{-1})**
(0:12)-Cu	5	0.1	0.063	2.78E-05	6.3	1.26	0.019	1.401
(6:6)-Cu	4.4	0.088	0.055	2.44E-05	7.5	1.5	0.024	1.036
(9:3)-Cu	2.8	0.056	0.035	1.55E-05	6.4	1.28	0.020	0.772
(10:2)-Cu	1.21	0.0242	0.015	6.72E-06	7.3	1.46	0.023	0.293
(12:0)-Cu	0.6	0.012	0.008	3.33E-06	6.2	1.24	0.019	0.171

[a] mmol. $g^{-1}.h^{-1}$, [b] mmol. h^{-1}, [c] mmol. h^{-1}. cm^{-2}, [d] mmol. s^{-1}.

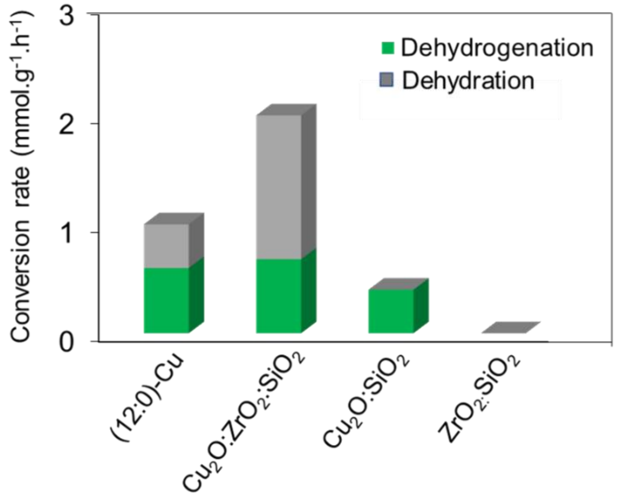

Figure S13. Performance of (12:0)-Cu relative to reference oxide samples. The performance of (12:0)-Cu, $Cu_2O:ZrO_2:SiO_2$, $Cu_2O:SiO_2$ and $ZrO_2:SiO_2$ in the photocatalytic dehydrogenation of FAc at steady state.

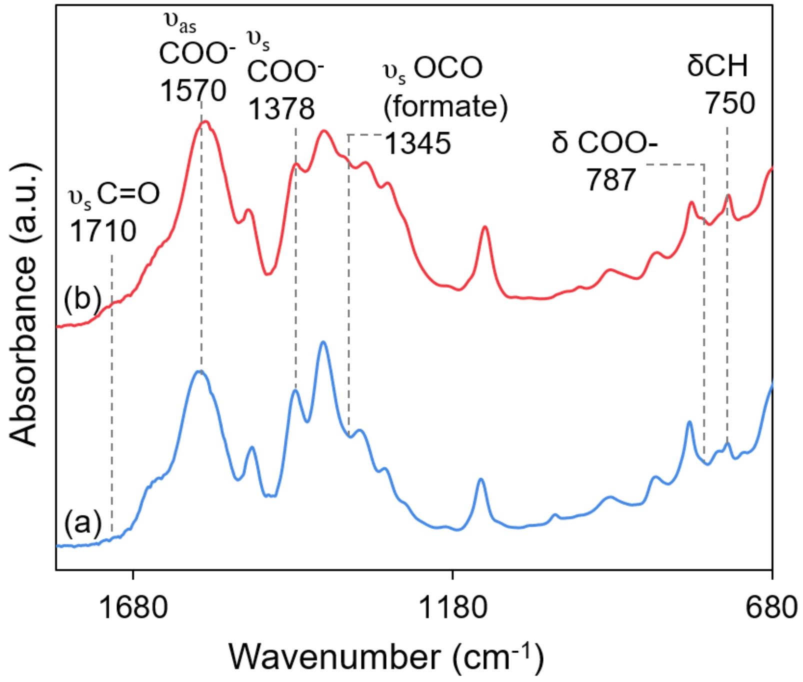

Figure S14. ATR-IR spectra of the (6:6)-Cu sample before (a) and after (b) reaction.

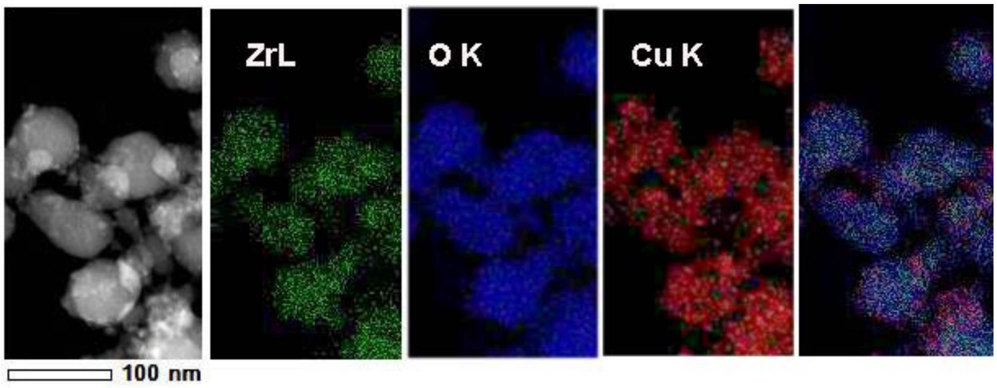

Figure S15. Low magnification HAADF-STEM image of (6:6)-Cu sample and simultaneously acquired EDX-STEM elemental maps for Zr L, O K, Cu K and overlaid color image

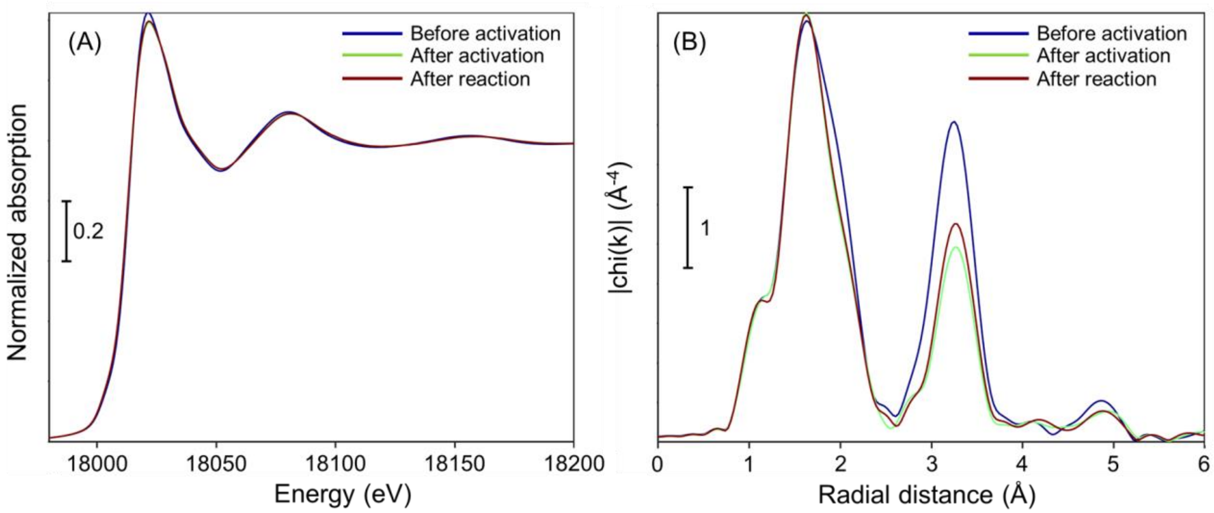

Figure S16. (A) The normalized Zr K-edge XANES spectra and (B) Fourier Transform of Zr K-edge EXAFS spectra of UiO-66-$(COOH)_2$ at different catalytic process at steady state.

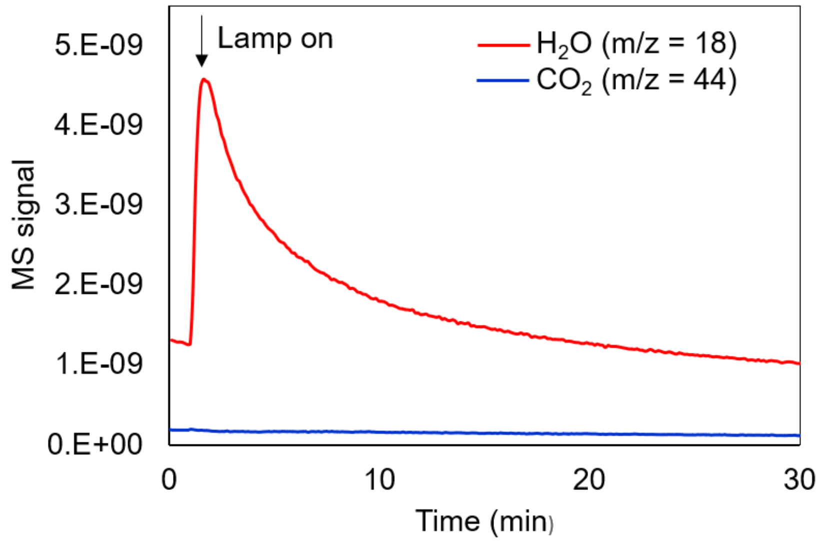

Figure S17. The evolution of the MS signals of H_2O and CO_2 during the activation process of the (0:12)-Cu sample.

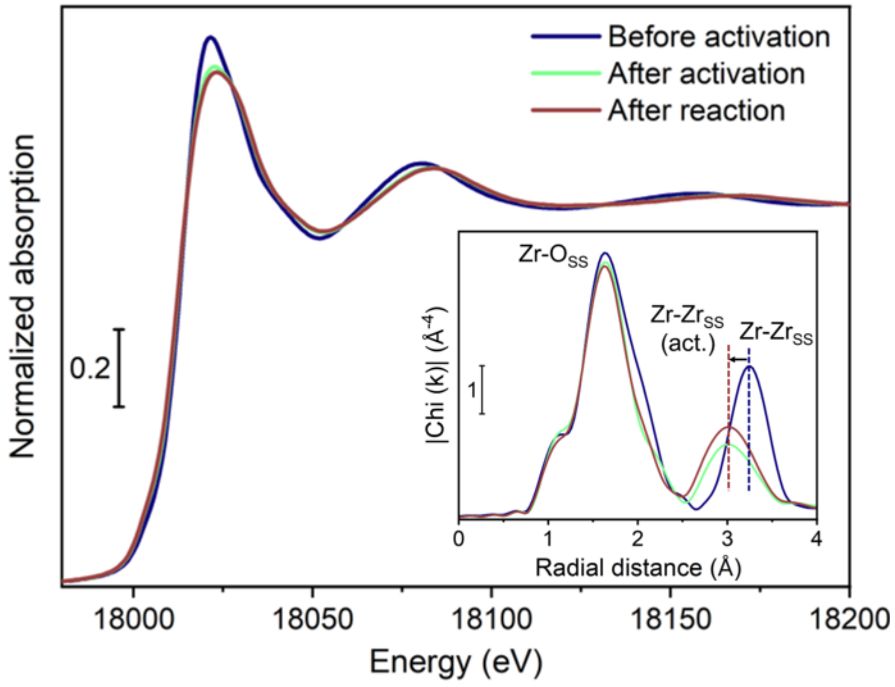

Figure S18. The normalized Zr K- edge XANES spectra at steady state in different steps (inset: corresponding Zr K- edge EXAFS spectra) of the (0:12)-Cu

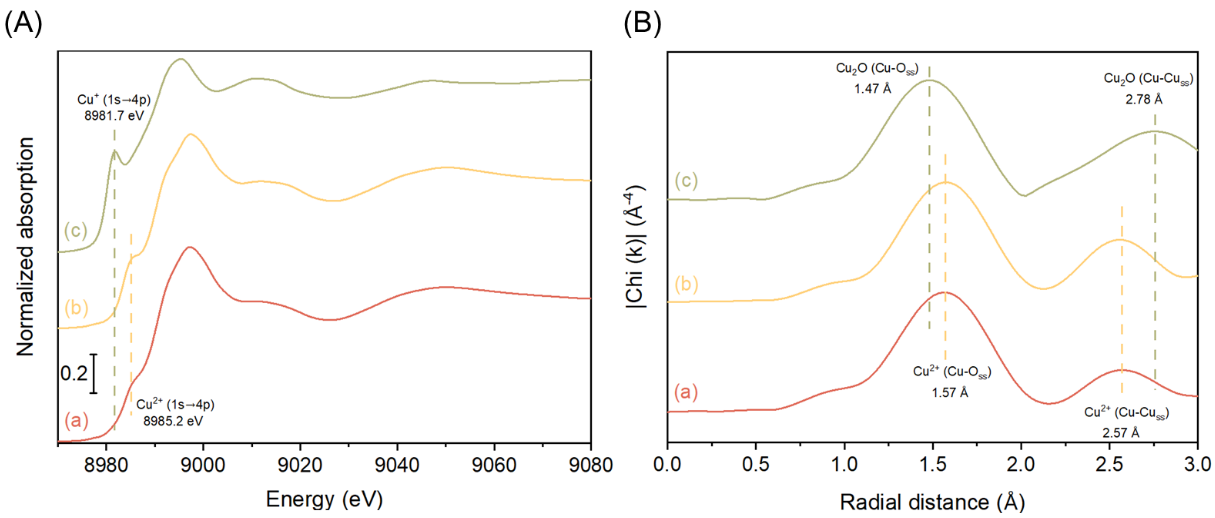

Figure S19. The XAS spectra of (a) pristine (0:12)-Cu, compared with (b) CuO and (c) Cu_2O, as references, (A) Cu-K edge XANES spectra, (b) FT-EXAFS spectra.

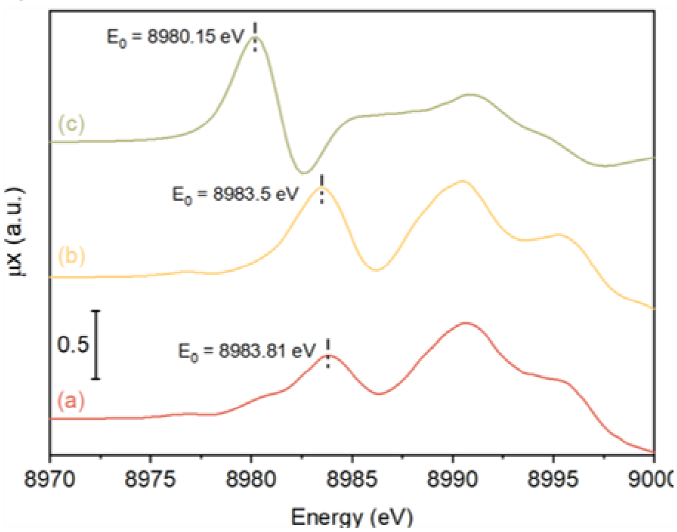

Figure S20. The 1st derivative of XANES spectra of operando XAS spectra of (a) pristine (0:12)-Cu, compared with (b) CuO and (c) Cu_2O, as references.

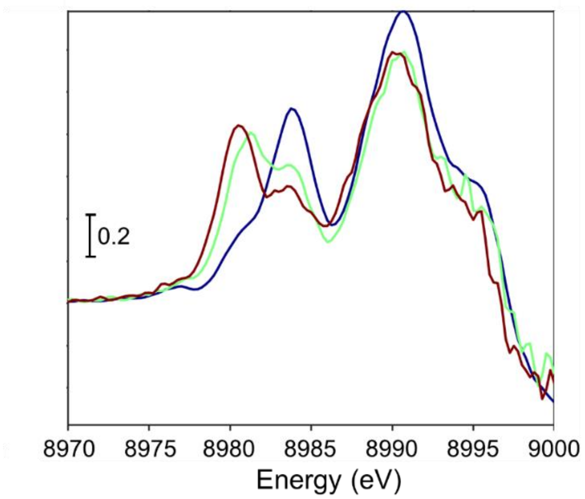

Figure S21: The first derivative of the Cu-K edge XANES spectra for (0:12)-Cu in its pristine state (blue), after light activation (≥ 390 nm) (green), and following FAc incorporation (red) on the MOF surface.

Methodology for MCR-ALS minimization

Three UiO-66(COOH)$_x$-Cu MOFs, (9:3)-Cu, (6:6)-Cu and (0:12)-Cu were monitored *operando* by quick-XAS at the Cu K-edge. Principle component analysis (PCA) was performed on each dataset indicates that three components were needed to explain the variance observed in each matrix of data. Multivariate curve resolution with alternating least square (MCR-ALS) was then applied simultaneously to the three datasets to resolve the pure spectra with independent concentrations profile corresponding to each experiment. The obtained spectra are represented in **Figure S24** together with the corresponding concentration profile (Cp1-Cp3). The Cp1 could be considered as small Cu (II) cluster in pristine Cu-metalated MOF. Cp2 is identified as Cu (I) signals based on the position of the rising edge and Cu-O distance, whereas, Cp3 is identified as Cu (0).

In first step, the MCR-ALS was performed independently on each data matrix. For each experiment, three components were required to explain the observed variance in the system.

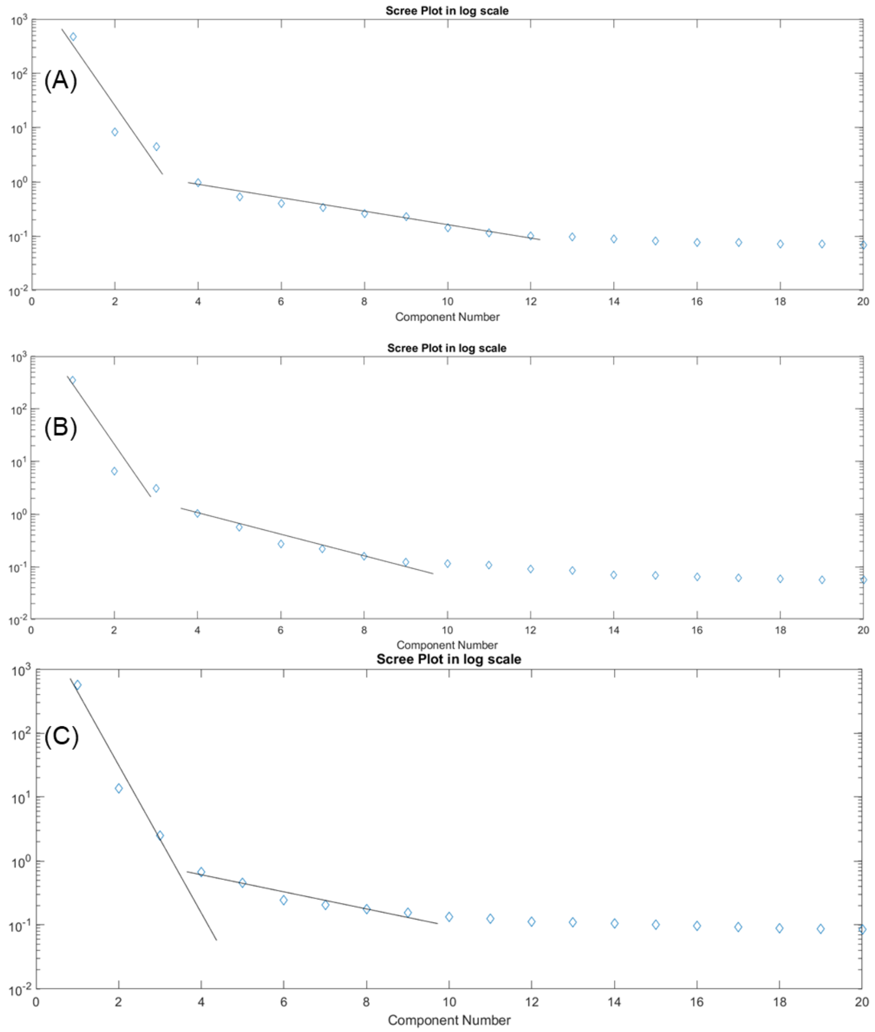

Figure S22. Screen plot (in log scale) obtained from PCA analysis for (a) (9:3)-Cu-, (b) (6:6)-Cu- and (c) (0:12)-Cu MOFs.

For (9:3)-Cu and (6:6)-Cu MOFs, the three components were similar: the component 1 (Cp1) (pristine Cu (II)) disappears to the benefit of component 2 (Cp2) (identified as Cu(I)), which in turns change for component 3 (Cp3) (identified as Cu (0)). However, the minimization performed on (0:12)-Cu MOF did not allow to extract pure spectra: it was possible to reproduce the obtained third component as a linear combination fitting of around 50% of Cp2 and 50% of Cp3 from (9:3)-Cu and (6:6)-Cu MOFs.

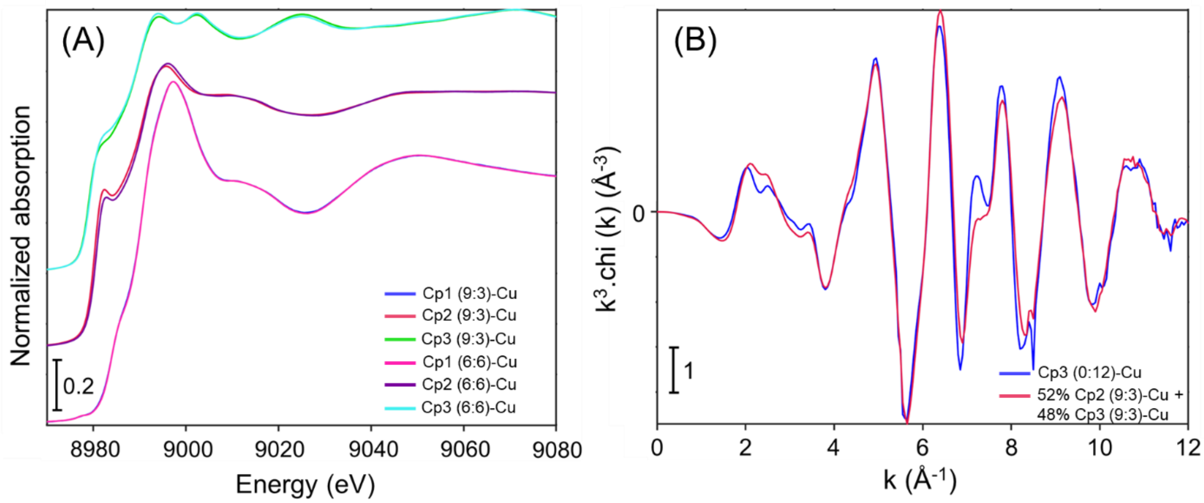

Figure S23. (A) Comparison of the XANES spectra obtained from MCR-ALS performed on (9:3)-Cu and (6:6)-Cu MOFs, and (B) comparison of the EXAFS signals for Cp3 obtained on (0:12)-Cu MOF with the linear combination of 50% of Cp2 and 48% of Cp3 from (9:3)-Cu-MOF.

As the three components from (9:3)-Cu and (6:6)-Cu were similar and that components from (0:12)-Cu can be explained as a linear combination of Cp2 and Cp3 from Manip11, the MCR-ALS was performed simultaneously on the three data matrices. With this approach, it is possible to resolve the three common pure components to all data matrices with independent concentration profiles for each dataset. To help the minimization, the first spectrum was imposed to be the spectrum of the pristine Cu-MOF, non-negativity of the spectra and the concentration profiles was imposed and the closure relation was applied for the concentration profiles.

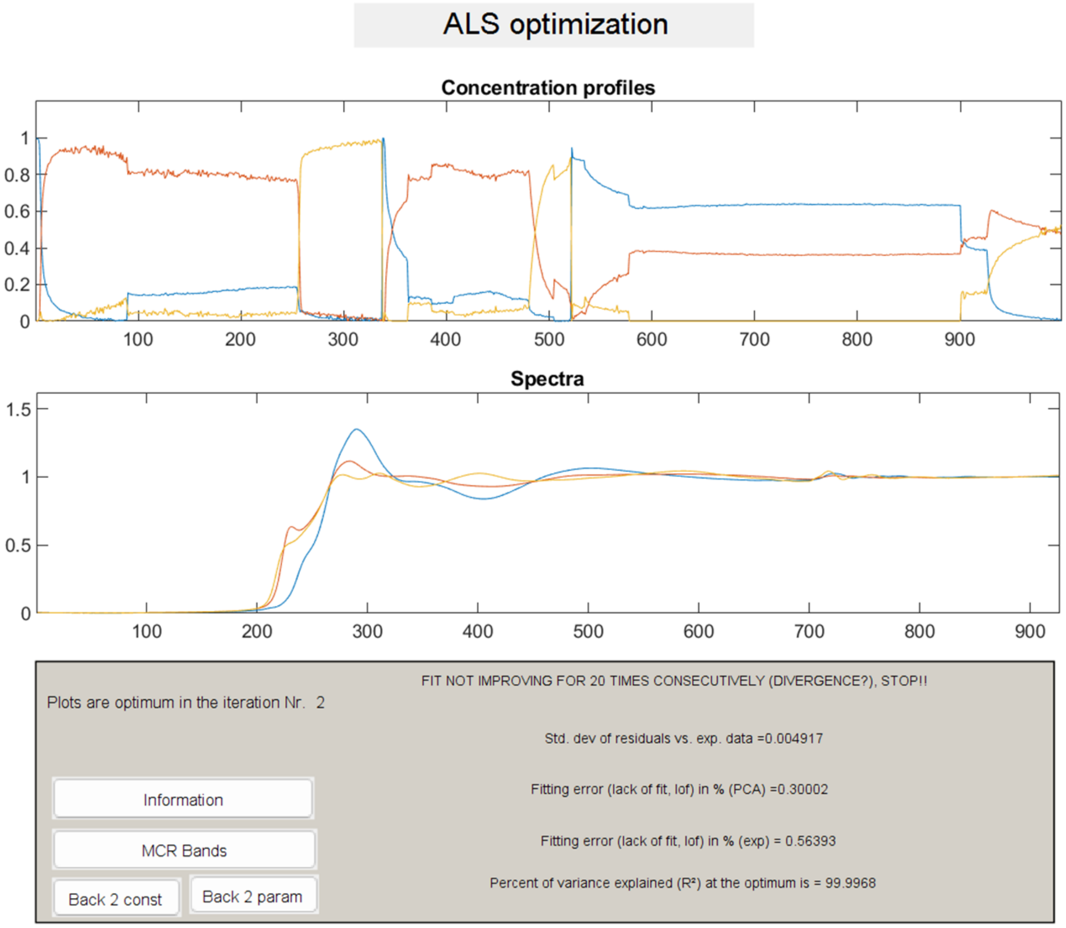

Figure S24. The output of the final MCR-ALS minimization performed on the three datasets corresponding to each experiment carried out on the three different MOFs, (9:3)-Cu, (6:6)-Cu, and (0:12)-Cu MOFs.

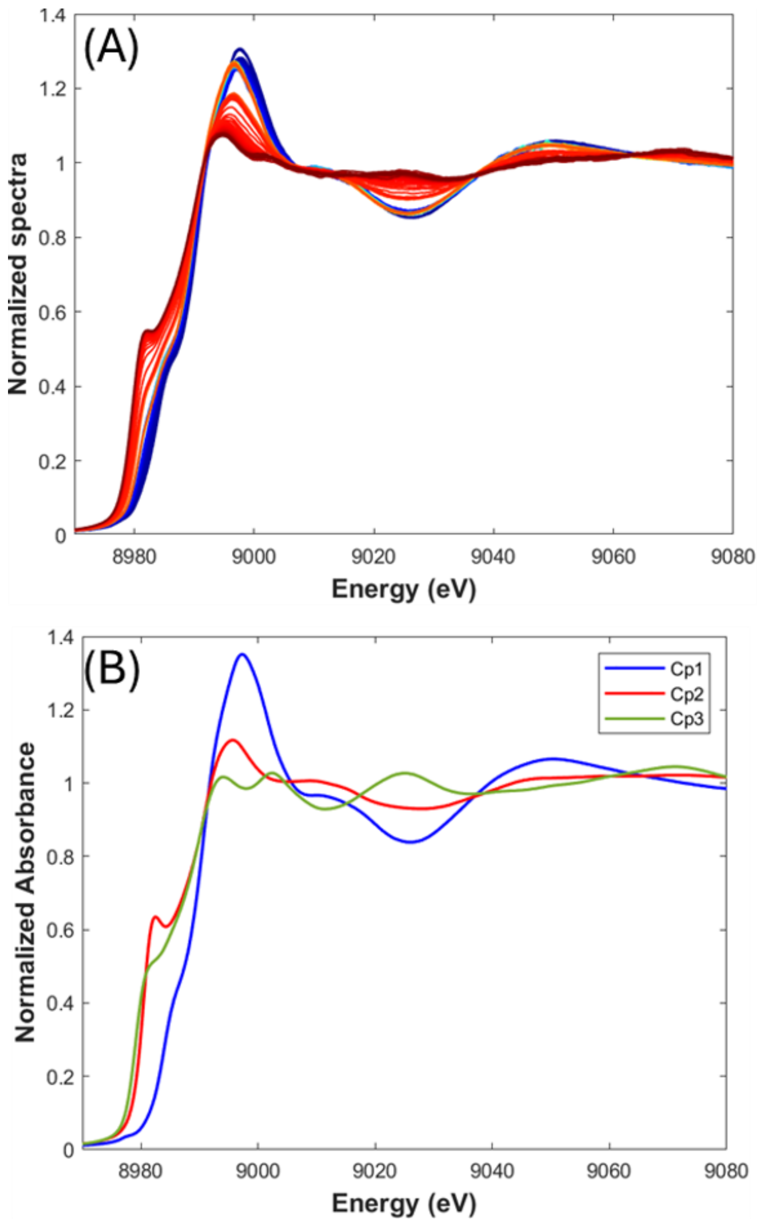

Figure S25. (A) Quick-XAS data obtained at the Cu K-edge for (0:12)-Cu sample during the three steps (activation of the MOF, FAc adsorption and photocatalytic reaction), (B) the XANES spectra obtained from MCR-ALS

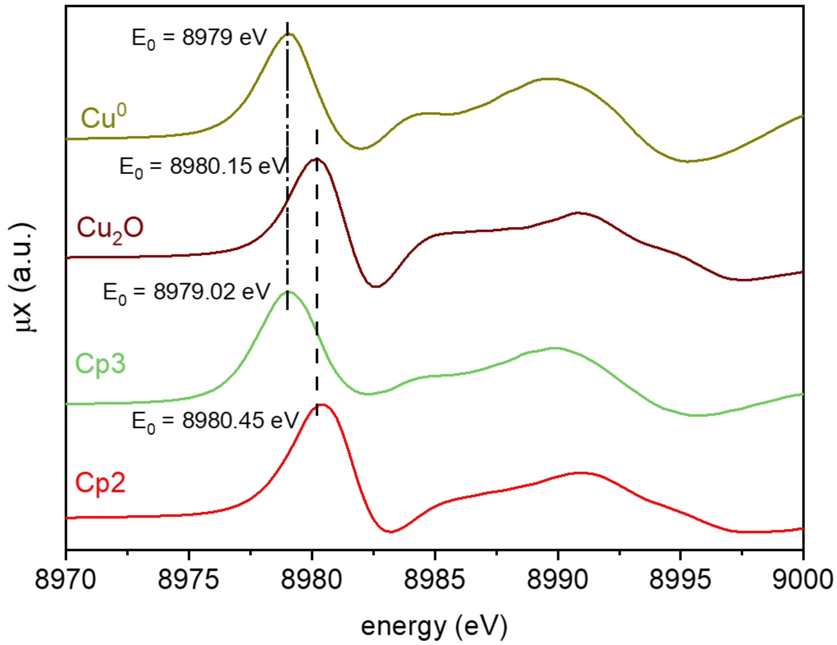

Figure S26. The 1st derivative of XANES spectra of operando XAS spectra of Cp2 and Cp3, compared with Cu_2O and metallic Cu, as references.

The calculations of the EXAFS fitting

In a first step, the EXAFS signal of CuO and a metallic Cu foil were fitted fixing their coordination number to fix the amplitude reduction factor S_0^2 *(the obtained value was 0.7).*

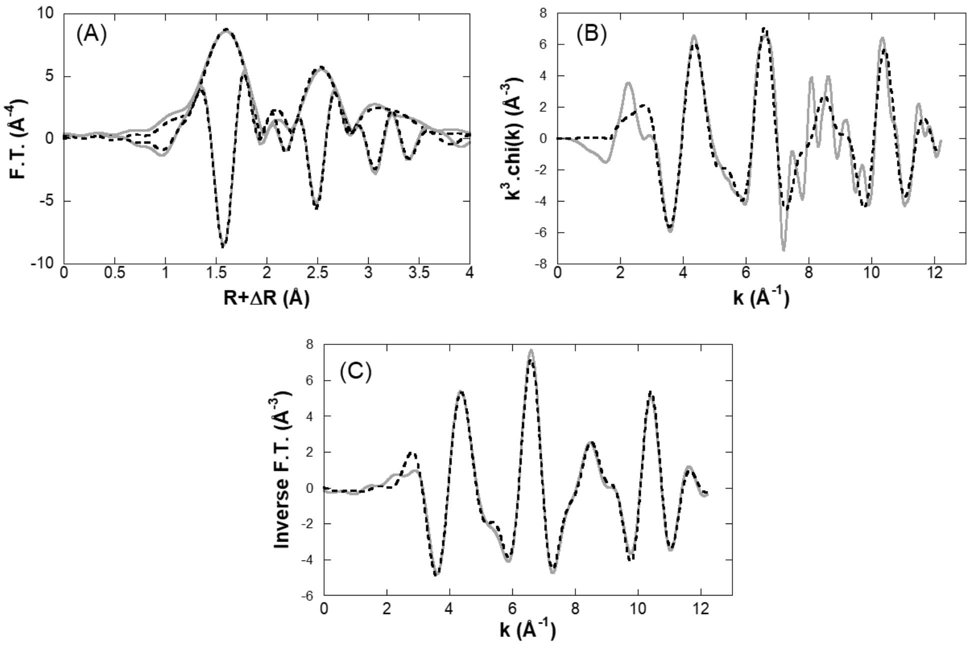

Figure S27. (A) Magnitude and imaginary part of the Fourier Transform, (B) EXAFS oscillations and (C) inverse Fourier Transform on the 1-3.8 Å range for the CuO reference. The grey solid lines are experimental data and the black dotted lines are fit results.

Table S2. Cu K-edge fit results for the CuO reference. Fixed parameters are denoted *. ** represents parameters for which the value was constrained to be the same. The reduction amplitude factor was S_0^2=0.7 and the energy shift is E_0 = 8979 + 11.2 eV. Fitting range=1-3.5 Å.

Path	CN	R (Å)	σ^2 (Å^2)
Cu-O	4*	1.95	0.003
Cu-O	2*	2.71	0.004
Cu-Cu	4*	2.89	0.003**
Cu-Cu	4*	3.04	0.003**
Cu-Cu	2*	3.18	0.003**
Cu-Cu	2*	3.45	0.011
Cu-Cu	2*	3.83	0.012

CN is the coordination number, R is the distance between the absorbing and scattering atom and σ^2 is the Debye-Weller factor.

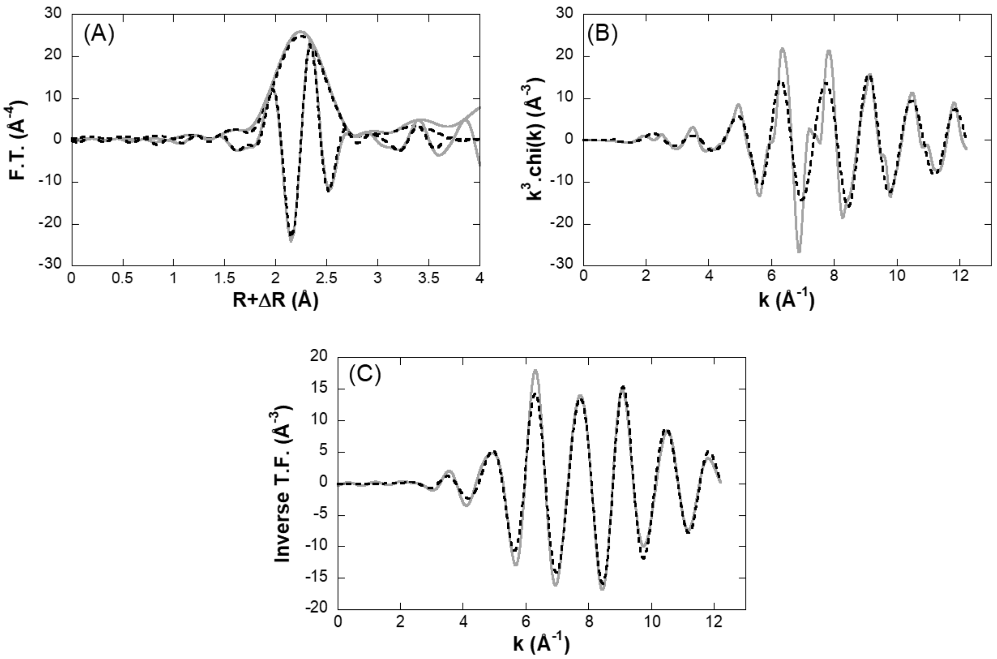

Figure S28. (A) Magnitude and imaginary part of the Fourier Transform, (B) EXAFS oscillations and (C) inverse Fourier Transform on the 1-3.5 Å range for the metallic copper foil. The grey solid lines are experimental data and the black dotted lines are fit results.

Table S3. Cu K-edge fit results for the metallic copper foil. Fixed parameters are denoted *. The reduction amplitude factor was $S_0^2=0.7$ and the energy shift is E_0 = 8979 + 4.3 eV. Fitting range=1-3.5 Å.

Path	CN	R (Å)	σ^2 (Å^2)
Cu-Cu	12*	2.54	0.007
Cu-Cu	6*	3.59	0.011

CN is the coordination number, R is the distance between the absorbing and scattering atom and σ^2 is the Debye-Weller factor.

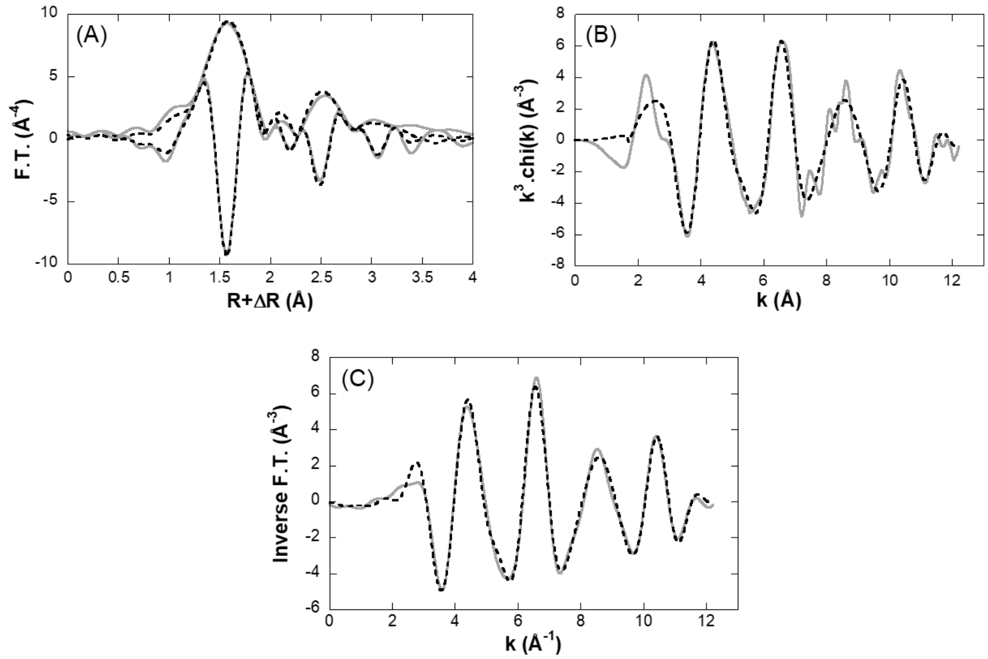

Figure S29. (A) Magnitude and imaginary part of the Fourier Transform, (B) EXAFS oscillations and (C) inverse Fourier Transform on the 1-3.5 Å range for the component 1 (Cp1). The grey solid lines are experimental data and the black dotted lines are fit results.

Table S4. Cu K-edge fit results for the component 1 (Cp1). Fixed parameters are denoted *. ** represents parameters for which the value was constrained to be the same. *** The CN of Cu-O paths was set to 6. The reduction amplitude factor was S_0^2=0.7 and the energy shift is E_0 = 8979 + 10.8 eV. Fitting range=1-3.5 Å.

Path	CN	R (Å)	σ^2 (Å^2)
Cu-O	5***	1.95	0.005**
Cu-O	1***	2.71	0.005**
Cu-Cu	4.3	2.9	0.008**
Cu-Cu	3.6	3.09	0.008**

CN is the coordination number, R is the distance between the absorbing and scattering atom and σ^2 is the Debye-Weller factor.

The EXAFS fitting revealed the first coordination shell of pristine Cu in the UiO-66$(COO)_2$-Cu is connected with oxygen atoms in a distorted octahedral fashion.

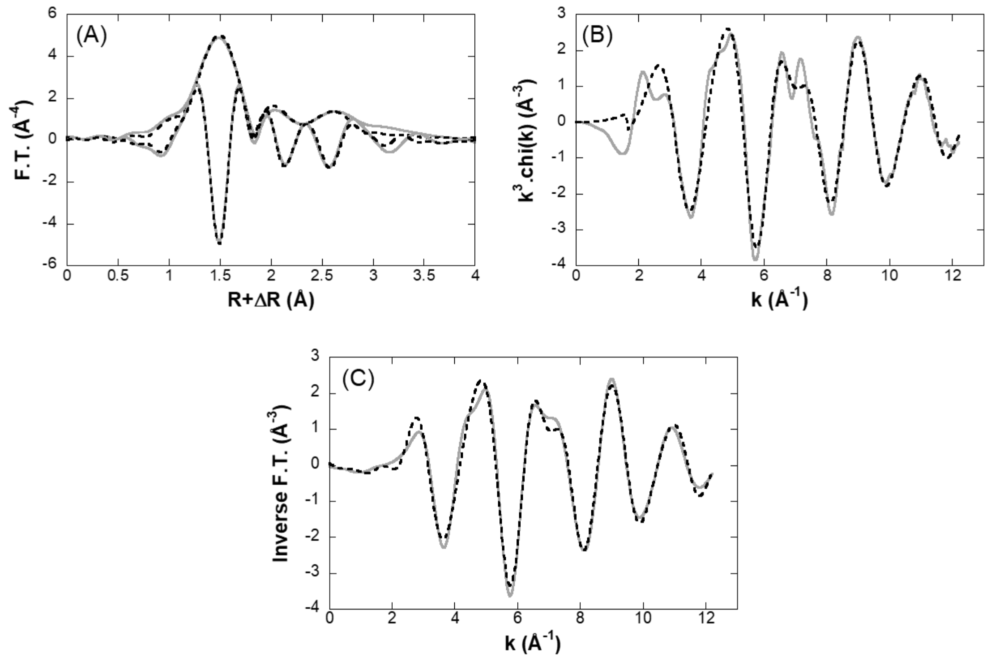

Figure S30. (A) Magnitude and imaginary part of the Fourier Transform, (B) EXAFS oscillations and (C) inverse Fourier Transform on the 1-3.5 Å range for the component 2 (Cp2). The grey solid lines are experimental data and the black dotted lines are fit results.

Table S5. Cu K-edge fit results for the component 2 (Cp2). ** represents parameters for which the value was constrained to be the same. The reduction amplitude factor was S_0^2=0.7 and the energy shift is E_0 = 8979 + 10.8 eV. Fitting range=1-3.5 Å.

Path	CN	R (Å)	σ^2 (Å^2)
Cu-O	2	1.86	0.003
Cu-Cu	0.5	2.55	0.005**
Cu-Cu	1.0	3.00	0.005**
Cu-Cu	0.7	3.17	0.005**

CN is the coordination number, R is the distance between the absorbing and scattering atom and σ^2 is the Debye-Weller factor.

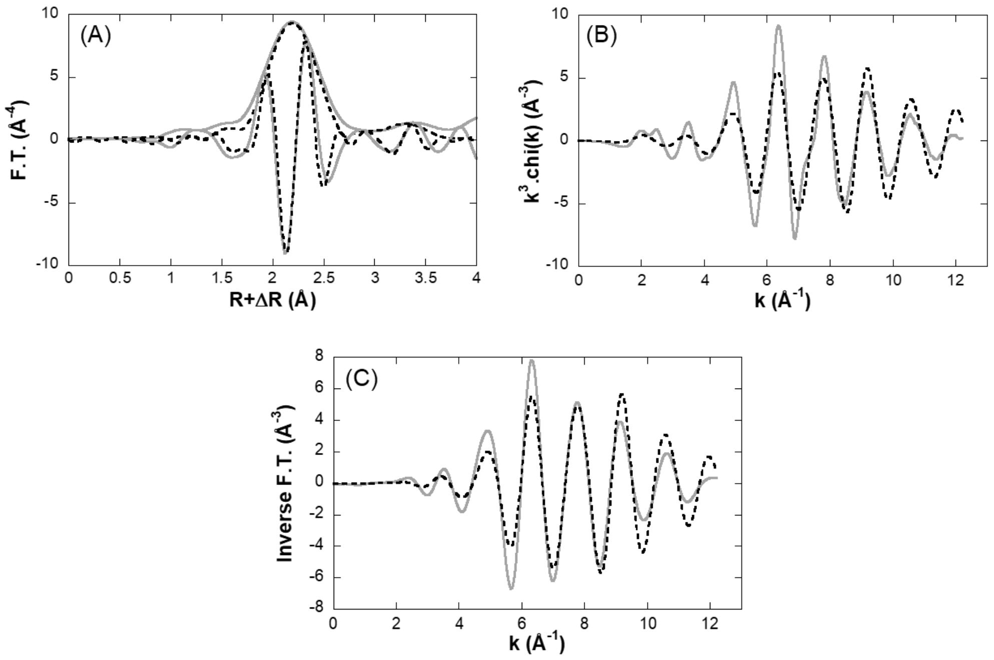

Figure S31. (A) Magnitude and imaginary part of the Fourier Transform, (B) EXAFS oscillations and (C) inverse Fourier Transform on the 1-3.5 Å range for the component 3 (Cp3). The grey solid lines are experimental data and the black dotted lines are fit results.

Table S6. Cu K-edge fit results for the component 3 (Cp3). Fixed parameters are denoted *. The reduction amplitude factor was S_0^2=0.7 and the energy shift is E_0 = 8979 + 0.57 eV. Fitting range=1-3.5 Å.

Path	CN	R (Å)	σ^2 ($Å^2$)
Cu-Cu	4.4	2.51	0.007*
Cu-Cu	2.1	3.53	0.011*

CN is the coordination number, R is the distance between the absorbing and scattering atom and σ^2 is the Debye-Weller factor.

The restructured Cu^0 was found to be in *fcc* geometry. The coordination number was found to be lower (4.4 and 2.1 in 1st and 2nd coordination shell) than the typical metallic Cu (12 and 6 in 1st and 2nd coordination shell), suggesting nano-sized metallic Cu cluster.

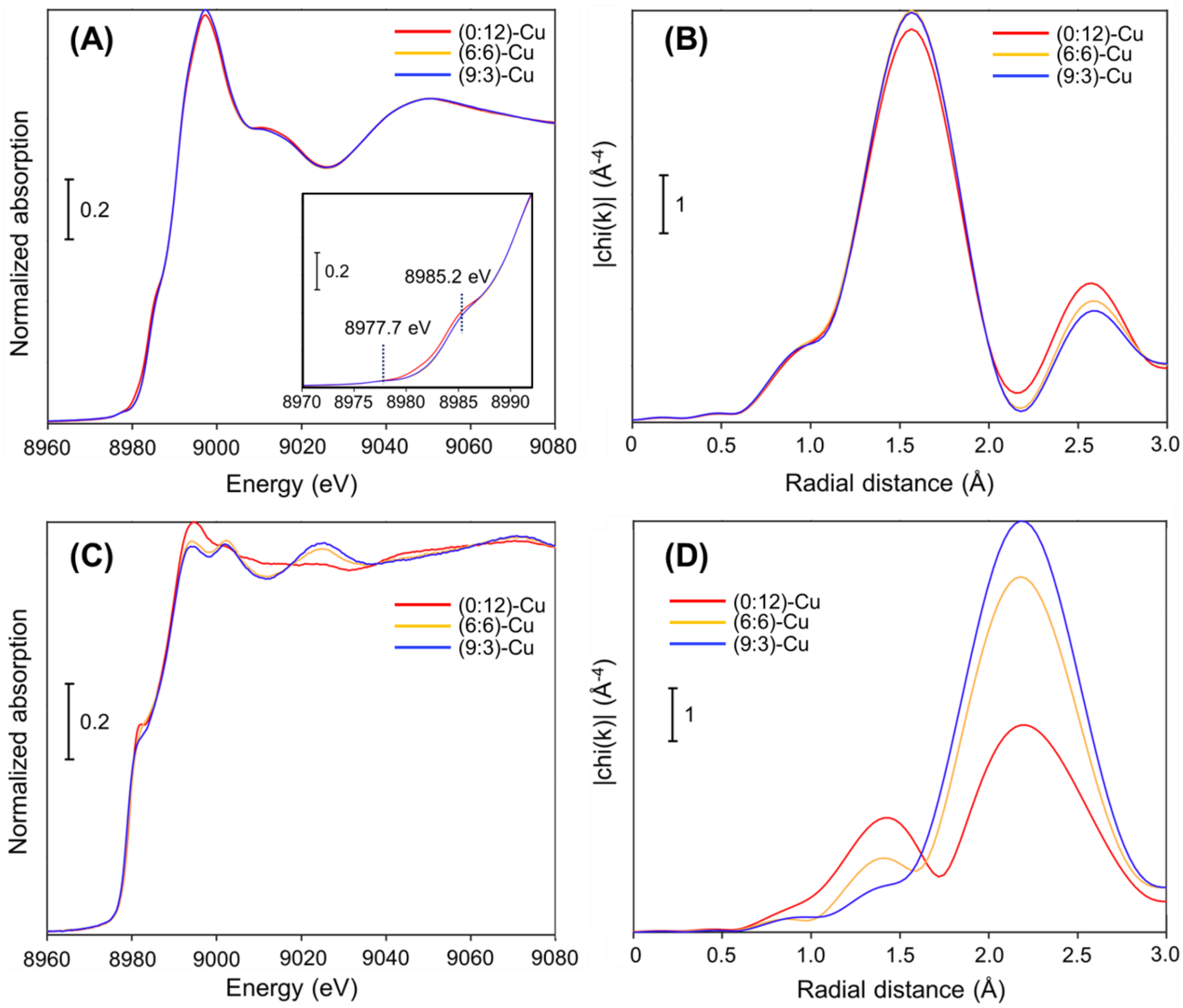

Figure S32. The normalized Cu K-edge XANES spectra of different UiO-66(COOH)$_x$ frameworks at (A) pristine state and (C) under catalytic reaction, and corresponding Cu K-edge EXAFS spectra at (B) pristine and (D) under reaction condition.

Table S7. The life time and the percentage of the excited species present in (0:12)-Cu before and after reaction at 430 nm emission wavelength.

MOF	Species	Lifetime (ns)	Percentage (%)
(0:12)-Cu-before reaction	1<br>2<br>3	$\tau_1 = 0.889$<br>$\tau_2 = 5.21$<br>$\tau_3 = 44.39$	32.23<br>26.02<br>41.05
(0:12)-Cu-after reaction	1<br>2<br>3	$\tau_1 = 0.48$<br>$\tau_2 = 3.3$<br>$\tau_3 = 40.3$	19<br>26<br>47

Table S8. The life time and the percentage of the excited species present in Cu_2O, Cu_2O/Cu^0 and (0:12)-Cu after reaction at 580 nm emission wavelength.

**Sample**	**Species**	**Lifetime (ns)**	**Percentage (%)**
Cu_2O	1	τ_1 = 1.625	11
	2	τ_2 = 13.05	24
	3	τ_3 = 60.44	65
(0:12)-Cu- after reaction	1	τ_1 = 0.52	34
	2	τ_2 = 2.9	51
	3	τ_3 = 16.9	15
Cu_2O/Cu^0	1	τ_1 = 0.5	26
	2	τ_2 = 3.4	55
	3	τ_3 = 25.6	19

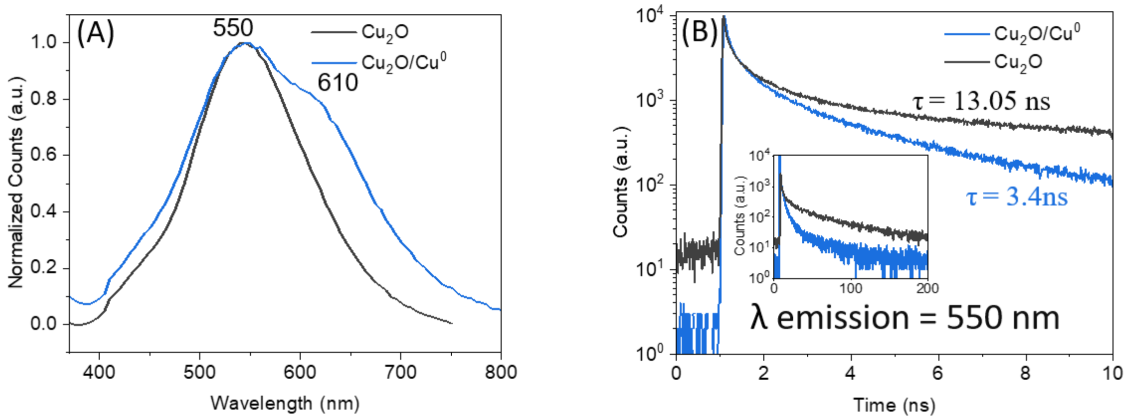

Figure S33. (A) The normalized PL spectra of Cu_2O and Cu_2O/Cu^0 upon excitation at 320 nm (inset: their corresponding normalized intensities) and (B) their corresponding decay life time at 550 nm upon 343 nm excitation, inset: zoomed-in view of the 200 ns time scale.

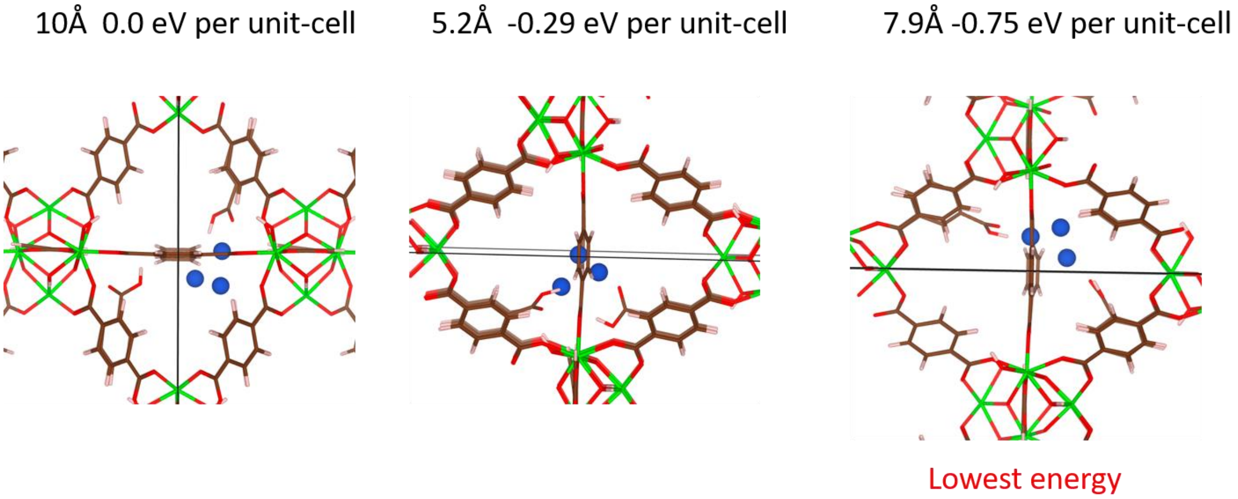

Figure S34. Schematic illustration of the possible configurations of the (0:12)-Cu MOF framework after Cu incorporation, showing three possible distance separating two free carboxylate groups. Color codes: C, brown; Cu, blue; O, red; H, white; Zr, green

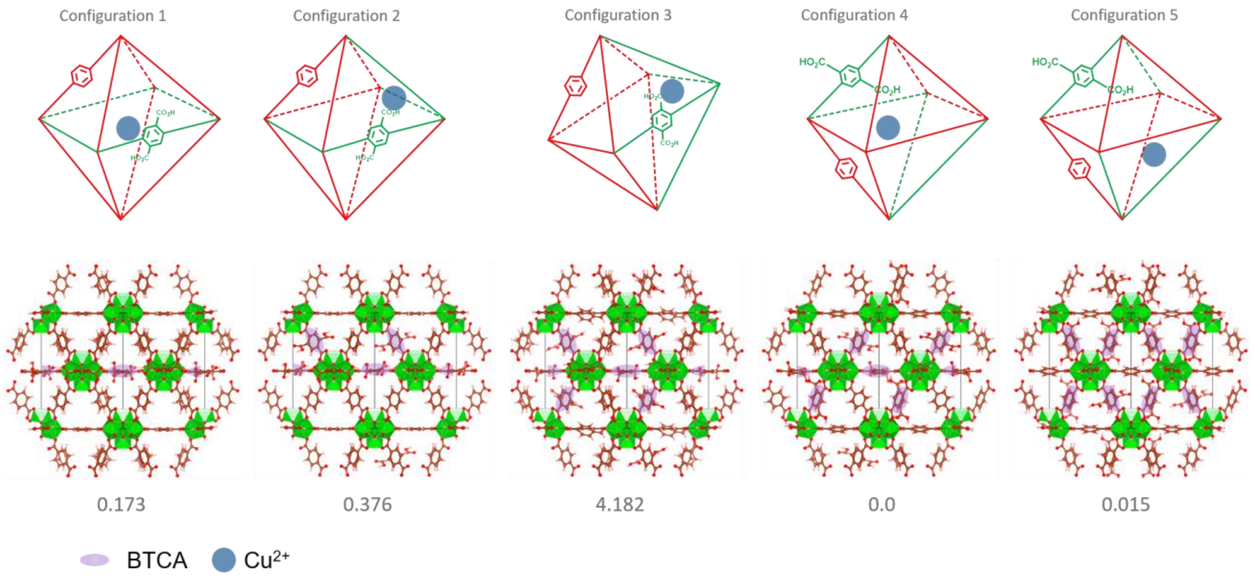

Figure S35. (Top) Schematic illustration of the possible configurations of the (8:4) sample (five typical BDC/BTCA combinations considered) and (Bottom) their corresponding DFT-optimized configurations. The relative energy value of the different configurations is also shown below with the energy unit eV/unit-cell. Color codes: C, brown; Cu, blue; O, red; H, white; Zr, green.

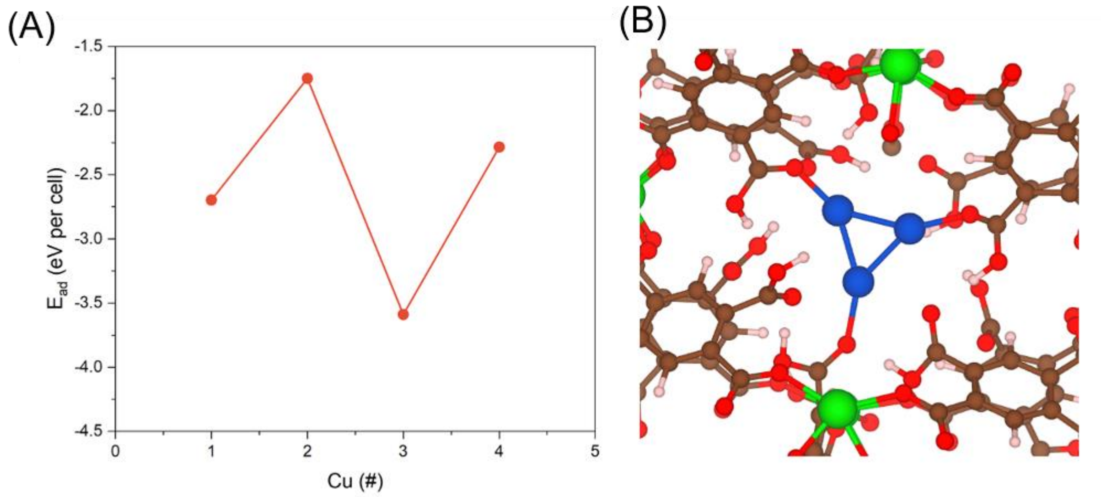

Figure S36. (A) The Cu adsorption energy under different Cu loading conditions. (B) Local atomic structure of the Cu3@ UiO-66(COOH)$_2$ structure.

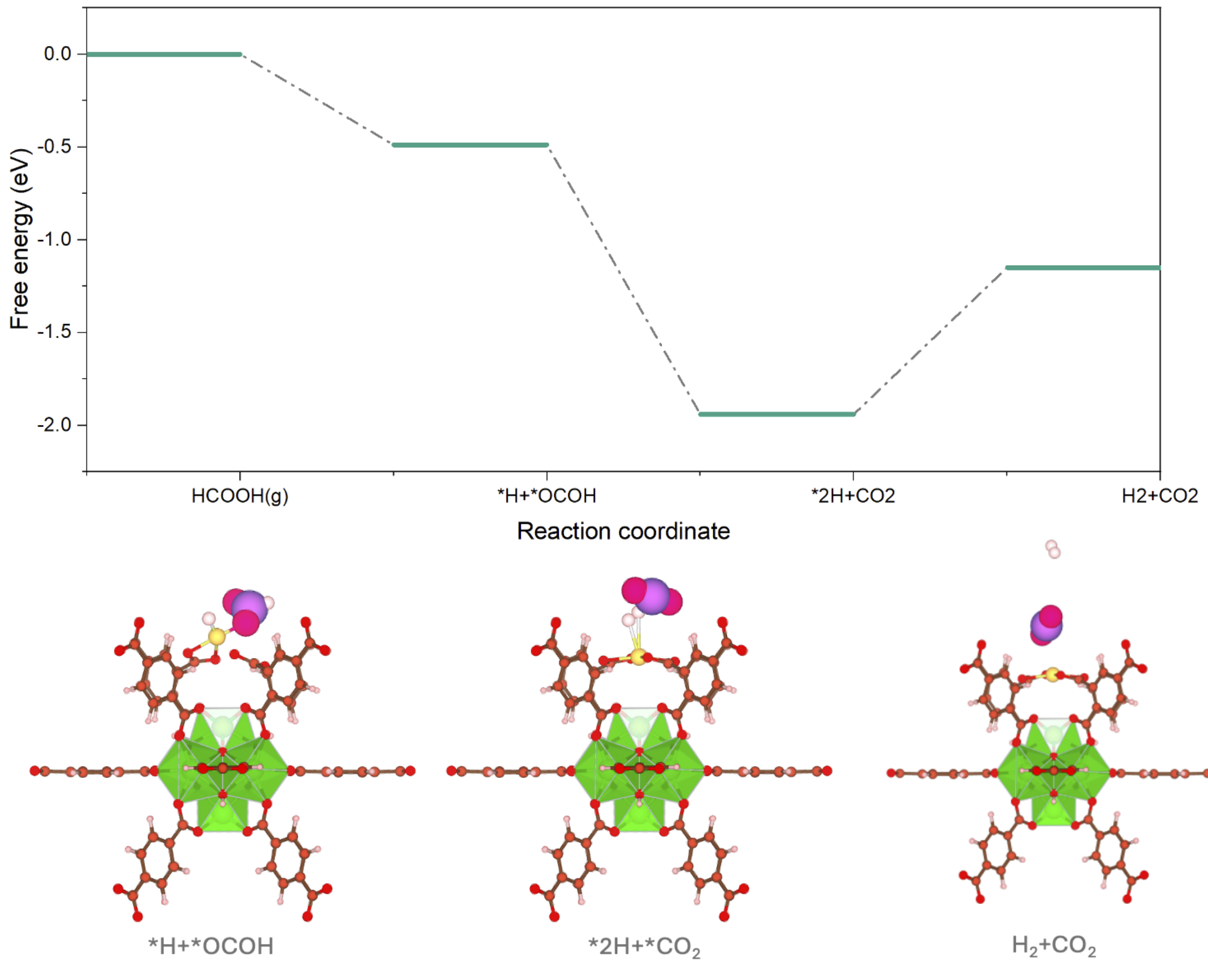

Figure S37. (Top) DFT-calculated minimum-energy pathway for the dehydrogenation of FAc by UiO-66$(COOH)_2$-Cu alongside the corresponding (Bottom) illustrative snapshots of the different intermediate species.

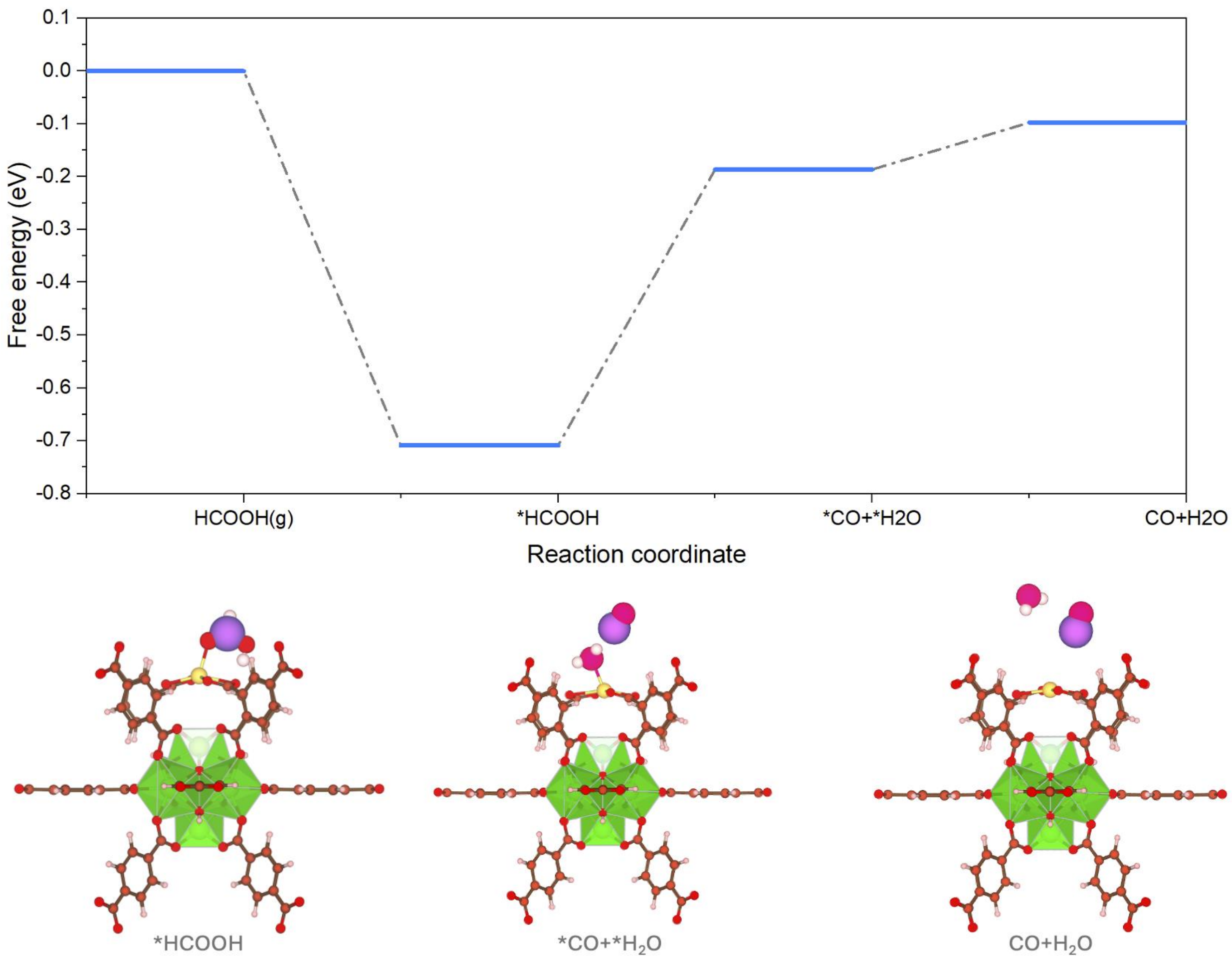

Figure S38. (Top) DFT-calculated minimum-energy pathway for the dehydration of FAc by UiO-66$(COOH)_2$-Cu alongside the corresponding (Bottom) illustrative snapshots of the different intermediate species.

References

1. Issa Hamoud, H. *et al.* Selective Photocatalytic Dehydrogenation of Formic Acid by an In Situ-Restructured Copper-Postmetalated Metal–Organic Framework under Visible Light. *J. Am. Chem. Soc.* **144**, 16433–16446 (2022).
